# Guidelines for diagnosis and treatment in neurology – Lyme neuroborreliosis

**DOI:** 10.3205/000279

**Published:** 2020-02-27

**Authors:** Sebastian Rauer, Stephan Kastenbauer, Heidelore Hofmann, Volker Fingerle, Hans-Iko Huppertz, Klaus-Peter Hunfeld, Andreas Krause, Bernhard Ruf, Rick Dersch, Consensus group

**Affiliations:** 1 German Society of Neurology (DGN), Berlin, Germany; 2 German Dermatology Society (DDG), Berlin, Germany; 3 German Society for Hygiene and Microbiology (DGHM), Münster, Germany; 4 German Society of Paediatrics and Adolescent Medicine (DGKJ), Berlin, Germany; 5 German Society of Paediatric Infectology (DGPI), Berlin, Germany; 6 The German United Society of Clinical Chemistry and Laboratory Medicine (DGKL), Bonn, Germany; 7 INSTAND e.V., Düsseldorf, Germany; 8 German Society of Rheumatology (DGRh), Berlin, Germany; 9 German Society of Infectious Diseases (DGI), Berlin, Germany; 10 Cochrane Germany, Faculty of Medicine, University of Freiburg, Germany

**Keywords:** Borrelia burgdorferi infection, Lyme borreliosis, Lyme disease, Bannwarth’s syndrome, lymphocytic meningoradiculitis, facial palsy, polyradiculitis, meningitis, encephalomyelitis, polyneuropathy, ixodid tick-borne borreliosis

## Abstract

Lyme borreliosis is the most common tick-borne infectious disease in Europe. A neurological manifestation occurs in 3–15% of infections and can manifest as polyradiculitis, meningitis and (rarely) encephalomyelitis.

This S3 guideline is directed at physicians in private practices and clinics who treat Lyme neuroborreliosis in children and adults.

Twenty AWMF member societies, the Robert Koch Institute, the German Borreliosis Society and three patient organisations participated in its development. A systematic review and assessment of the literature was conducted by the German Cochrane Centre, Freiburg (Cochrane Germany).

The main objectives of this guideline are to define the disease and to give recommendations for the confirmation of a clinically suspected diagnosis by laboratory testing, antibiotic therapy, differential diagnostic testing and prevention.

## Zusammenfassung

Die Lyme-Borreliose ist die häufigste durch Zecken übertragene Infektionskrankheit in Europa. Eine neurologische Manifestation kommt bei 3–15% der Infektionen vor und kann sich als Polyradikulitis, Meningitis sowie selten als Enzephalomyelitis manifestieren. Die Erkrankung ist durch Antibiotika behandelbar.

Diese S3-Leitlinie richtet sich an Ärzte in Praxis und Klinik, die mit der Behandlung der Neuroborreliose bei Kindern und Erwachsenen befasst sind.

An der Entwicklung waren 20 AWMF-Mitgliedsgesellschaften, das Robert Koch-Institut, die Deutsche Borreliose-Gesellschaft und 3 Patientenorganisationen beteiligt. Die systematische Literaturrecherche und Literaturbewertung wurden durch das Deutsche Cochrane Zentrum Freiburg (Cochrane Deutschland) durchgeführt.

Hauptziele der Leitlinie sind es, die Krankheit zu definieren und Empfehlungen abzugeben zur Bestätigung der Verdachtsdiagnose mittels Laboruntersuchungen, Antibiotikabehandlung, Differenzialdiagnostik und Prävention.

## Preamble

This guideline pertains to the diagnosis and treatment of neurological manifestations of Lyme borreliosis in children and adults. In the future it will be integrated as module 2 of the planned interdisciplinary S3 guideline **“Lyme Borreliosis – Diagnosis and Treatment, AWMF Register No. 013-080”**.

Twenty AWMF member societies, the Robert Koch Institute, the German Borreliosis Society and three patient organisations participated in its development. A systematic review and assessment of the literature was conducted by the German Cochrane Centre, Freiburg (Cochrane Germany) with significant input from Dr. Rick Dersch.

The interdisciplinary guideline group is currently developing the S2k guideline “Cutaneous Lyme Borreliosis” (AWMF Register No. 013-044) [1] into an S3 guideline with the aim of modularly integrating it into the interdisciplinary guideline. Part 3 “Lyme Arthritis, Lyme Carditis and Other Rare Manifestations” will then be developed as a further module of the interdisciplinary S3 guideline “Lyme Borreliosis – Diagnosis and Treatment”. The guideline was formally adopted by the boards of the participating organisations with the exception of the DBG and the patient organisations BFBD, BZK and OnLyme-Aktion.org. The DBG and the patient organisations BFBD, BZK and OnLyme-Aktion.org have issued statements of dissent, which are published in an appendix (Attachment 1 ) to the guideline report (Attachment 2).

### What’s new?

- The previous S1 guideline on Lyme neuroborreliosis (AWMF Register No. 030-071) [2] has been developed into an S3 guideline on Lyme neuroborreliosis according to the methodological guidelines of the Association of Scientific Medical Societies (AWMF).
- The validity of the guideline has been extended to include the diagnosis and treatment of Lyme neuroborreliosis in children based on a systematic review [3].
- A systematic review of antibiotic treatment of Lyme neuroborreliosis in adults [4] found the following:
  - There is no scientific basis for deviating from the previously recommended treatment duration of 14 days for early and 14–21 days for late Lyme neuroborreliosis.
  - In the case of early Lyme neuroborreliosis, doxycycline and beta-lactam antibiotics (penicillin G, ceftriaxone and cefotaxime) are equally effective in terms of the regression of neurological symptoms and are tolerated equally.
  - There are no reliable, analysable study data on the efficacy of combination antibiotic therapy.
  - There are no study data available on the efficacy of chloroquine, carbapenems and metronidazole.
- A systematic review has found that the high prevalence of persisting non-specific and/or atypical symptoms following Lyme neuroborreliosis, as reported in many studies, can largely be traced to study artefacts resulting from imprecise case definitions [5].

### Key recommendations

- A suspected clinical diagnosis of neuroborreliosis (cranial nerve deficits, meningitis/meningoradiculitis, encephalomyelitis) can be confirmed by the detection of inflammatory changes in cerebrospinal fluid linked to Borrelia-specific intrathecal antibody synthesis.
- Serological testing should only be conducted if there is sufficient clinical suspicion. ↑↑ (consensus 10/13)
- The following antibiotics should be used to treat early and late Lyme neuroborreliosis: doxycycline, ceftriaxone, cefotaxime, penicillin G. ↑↑ (consensus 9/13)
- Antibiotic treatment should last 14 days (early Lyme borreliosis) or 14–21 days (late Lyme borreliosis). ↑↑ (strong consensus 13/13)
- Estimation of treatment success should be based on the clinical symptoms. ↑↑ (strong consensus 12/12)

### Preface

Lyme borreliosis is the most common tick-borne infectious disease in Europe. A neurological manifestation occurs in 3–15% of infections and can manifest as polyradiculitis, meningitis and (rarely) encephalomyelitis. The disease can be treated with antibiotics.

### Target group

This guideline is directed at physicians in private practices and clinics who treat Lyme neuroborreliosis in children and adults.

### Objectives of this guideline (recommendations)

- Definition of the disease
- Confirmation of a clinical diagnosis
- Differentiation of non-specific complaints
- Antibody testing in serum
- Cerebrospinal fluid (CSF) testing including antibody detection in CSF
- Meaningful use of molecular-diagnostic testing and culture tests
- Therapy
- Differential diagnostic testing
- Prevention
- Observation of the skin area around the tick bite; information sheet for patients
- Diseases caused by relapsing fever Borrelia (e.g. Borrelia recurrentis) are not covered in this guideline
- Questions relating to co-infections linked to diseases transmitted by ticks are not covered in this guideline

## 1 Epidemiology, transmission, manifestations, prophylaxis

### 1.1 Epidemiology

#### 1.1.1 Definition

Lyme borreliosis is a multisystem inflammatory disease that is caused by an infection with the spirochete *Borrelia burgdorferi* sensu lato and transmitted through the bite of the Ixodes ricinus tick.

#### 1.1.2 Distribution and species

It is the most prevalent vector-borne disease in the temperate climate zones of the northern hemisphere and is endemically widespread. In North America, Lyme borreliosis is only caused by the Borrelia species *Borrelia burgdorferi *sensu stricto, while in Europe *B. afzelii*, *B. bavariensis* and *B. garinii* have also been identified as human pathogens. The newly identified species *Borrelia spielmanii* also has the potential of being pathogenic to humans. It was detected in 4 of 160 skin isolates (all from erythema migrans) but has yet to be linked to Lyme neuroborreliosis (72 CSF isolates) in Germany [6]. The pathogenic potential of the various *Borrelia burgdorferi* species varies [7]. After *B. garinii* OspA-type 4 was reclassified as the new species *Borrelia bavariensis* [8], a re-evaluation of 242 human isolates from Germany [6] found that the 72 CSF isolates comprised 21% *B. afzelii*, 22% *B. bavariensis* and 29% *B. garinii*, and the 160 skin isolates comprised 67% *B. afzelii*, 12% *B. bavariensis* and 12% *B. garinii*. In other words, only the skin isolates showed a clear prevalence of one species, namely *B. afzelii*.

Currently no reliable figures are available on the rate of occurrence of Lyme borreliosis in individual European countries. An evaluation of population registers from six eastern German states found a strongly fluctuating rate of 34.9 cases per 100,000 inhabitants in 2009, and 19.54 cases per 100,000 inhabitants in 2012 [9]. Secondary data analyses of health insurance data based on ICD 10 code A 69.2 (G) found significantly higher case numbers, although the authors cannot rule out an overestimation of case numbers due to clinical misdiagnosis or miscoding [10].

In summary, the available epidemiological data are inconclusive. Data published in Germany to date suggest an incidence of Lyme borreliosis ranging from 60,000 to >200,000 cases per year.

#### 1.1.3 Incidence of various manifestations

Acute Lyme neuroborreliosis (3.3%) was the second most frequent clinical manifestation after Erythema migrans (95.4%) [9]. In a prospective, population-based study conducted in the Würzburg area, 313 cases of Lyme borreliosis were identified over a period of 12 months. This corresponds to a significantly higher incidence rate of 111 per 100,000 inhabitants and results in the following frequencies of manifestations [11]:

##### Early manifestations

- 89% erythema migrans (erythema migrans related to another organ manifestation in a further 3%)
- 3% Lyme neuroborreliosis (stage II)
- 2% Borrelia lymphocytoma
- <1% carditis

##### Late manifestations

- 5% Lyme arthritis
- 1% acrodermatitis chronica atrophicans
- Late Lyme neuroborreliosis (stage III) was not identified.

According to one study, children have a higher risk of developing Lyme neuroborreliosis after a tick bite than adults, most likely because they are more frequently bit on the head [12].

#### 1.1.4 Seroprevalence of Borrelia-specific antibodies

Borrelia-specific antibodies are found in 5–20% of healthy individuals in Germany and Austria depending on endemic region and age group [13], [14], [15]. A seroprevalence of 20% was found in 964 (asymptomatic) Swiss orienteers; in asymptomatic blood donors it was 8% [16]. A cross-sectional German study of children and adolescents aged 1–17 years found an average seroprevalence of 4.8%. The relative probability of a positive result for antibodies depended on age and increased for every year of life by 6% for girls and 11% for boys [17]. An elevated level of Borrelia-specific IgG antibodies was found in 20% of men >60 [15].

#### 1.1.5 Infection rates of ticks

Studies of ticks in southern Germany showed average infection rates of about 1% for larvae, 10% for nymphs and 20% for adults [18]. In addition to regional differences in tick-borne infection rates (18–37% for adults and 5–12% for nymphs), there were also significant differences in the regional distribution of the Borrelia species [6]. Infection rates in Switzerland were 5–7% depending on the area [19]. The density of infected ticks also varies greatly from region to region, ranging from 2 to 58 per 100 m^2^ in Switzerland. In addition to Lyme borreliosis, other infectious diseases such as TBE, human granulocytic anaplasmosis, rickettsiosis, ehrlichiosis etc. can be transmitted by ticks.

#### Summary

- Lyme borreliosis is a multisystem disease that is transmitted through the bite of the *Ixodes ricinus* tick. It primarily affects the skin, nervous system or joints.
- Five Borrelia species pathogenic to humans have so far been identified in Europe.
- There are no reliable figures on the rate of occurrence (incidence from different surveys in Germany 60,000 to >200,0000 cases/year).
- Seroprevalence of Borrelia-specific antibodies is 5–20%. It varies regionally and is age-dependent.
- Infection rates of ticks are area-dependent: 18–37% for adults, 5–12% for nymphs, 1% for larva.

### 1.2 Route of infection

Borrelia are transmitted through the bite of hard-bodied ticks (in Europe through the “castor bean tick” *Ixodes ricinus*). According to data from animal experiments, the risk of infection increases with the duration of the blood meal. It is not possible to reliably deduce the earliest point in time that an infection can be expected, especially since the probability of transmission even appears to vary from species to species [20]. The transmission mechanism of the Borrelia that survived in the tick’s intestine before the blood meal is very complex [21]. According to German studies, a seroconversion is expected to occur after a tick bite in 2.6–5.6% of those affected, and disease will manifest in 0.3–1.4% [22], [23], [24]. A study conducted in western Switzerland found the risk of being infected with Borrelia from a tick bite was just under 5% [25].

### 1.3 Prophylaxis

(Cited from DDG S2k Guideline “Cutaneous Lyme Borreliosis”; AWMF Register No. 013-044 [1].)

#### 1.3.1 Prevention of Lyme borreliosis

It is very important to **remove ticks early** before they become engorged. The risk of transmission of Borrelia increases with the length of time the tick sucks [26]. Transmission within the first 12 hours has rarely been observed in laboratory animals. The body should be checked in the evening for ticks after spending time in a garden, park, field, forest or meadow, where contact with a tick may have occurred.

Ticks should be removed immediately with a tick tweezer or a tick card in order to prevent the transmission of the Borrelia. If parts of the suction organ remain in the skin, they can be removed later with a needle or a curettage [27]. If the head or the suction organ remains in the skin, the risk of a Borrelia transfer does not increase. The bodies of nymphs and adult ticks should not be squeezed when they are engorged with blood in order to prevent a possible transfer of the Borrelia. Examination of the skin-removed tick for Borrelia is not useful, as detection of Borrelia in the tick does not provide sufficient predictive value for Borrelia transmission to the host nor for disease development. After the removal of a tick, the patient should be informed about the **necessary follow-up of the tick bite site** in the following 6 weeks (Appendix 6: “Patient information after a tick bite” in Attachment 3 ).

#### 1.3.2 Prophylactic treatment after a tick bite

According to an American study, the risk of infection after a tick bite can be reduced through a one-time prophylactic administration of 200 mg of doxycycline (87% effectiveness) [28], [29]. The results, however, should be interpreted with caution since only one follow-up was conducted after 6 weeks. Thus, no statement can currently be made as to whether this is sufficient with respect to a late infection.

In view of the low risk of disease, a large number of unnecessary doxycycline treatments would have to be accepted in order to prevent one potential infection. According to projections of infection risk in endemic areas, 40–125 prophylaxes would have to be administered in order to prevent 1 disease [30]. Impacts on the intestinal flora and a possible development of resistance through frequent prophylaxis is conceivable. Therefore, oral doxycycline prophylaxis is not recommended in Europe. The prophylactic application of an antibiotic cream is also controversial. Animal studies with azithromycin cream reveal a good prophylactic efficacy [31], [32]. A placebo-controlled study on its effectiveness in humans identified no prophylactic effect [33]. Therefore, this treatment is not recommended.

##### Recommendations for preventing infection

(Taken from the S2k guideline “Cutaneous Lyme Borreliosis” [1]).

- Clothing that covers the body should be worn to prevent tick bites. ↑
- Using tick repellents can be recommended with some reservations. ↔
- Skin should be inspected in the evening for ticks after being in an outdoor area where there is the possibility of contact with ticks. ↑↑
- Ticks should be removed early in order to prevent Lyme borreliosis. ↑↑
- The site of the bite should be observed for up to six weeks. ↑↑

##### Not recommended

- Testing the removed tick for Borrelia is not recommended. ↓
- No local or systemic prophylactic antibiotic treatment should be given after a tick bite. ↓

#### 1.3.3 Vaccines

No vaccine is currently approved for use in humans.

A vaccination with recombinant lipidated Osp A has been evaluated in the USA as part of a major study and has shown to be effective [34], [35]. The vaccine has been approved in the USA since 1999; however, it was taken off the market by its manufacturer. The reason for the withdrawal were economical. Reports on undesired vaccine reactions in individuals with a genetic predisposition were refuted by multiple qualified studies [36], [37], [38]. This monovalent vaccine is not suitable for Europe as it only protects against an infection with *B. burgdorferi *sensu stricto, and not against the genospecies *B. afzelii* and *B. garinii* that are frequently found in Europe.

A polyvalent OspA vaccine is currently being developed for Europe [39], however approval is not expected in the foreseeable future.

## 2 Symptoms

### 2.1 Possible stages

Early localised stage: An early Borrelia infection manifests in 80–90% of patients as local erythema migrans (early localised stage) [9], [11]. General symptoms such as feeling ill, arthralgia, myalgia, subfebrile temperatures or night sweats may occur a few days to weeks after a Borrelia infection [40].

**Early disseminated stage:** A disseminated infection can occur weeks to months after a tick bite (erythema migrans is only reported in around 25–50% of the acute cases of Lyme neuroborreliosis [41], [42], [43]), which predominantly affects the nervous system, joints and heart (early disseminated stage) [40].

**Late manifestations:** In rare cases, a late or chronic manifestation with involvement of the skin, the nervous system and the joints (late manifestations) can occur after months or years [40], [44], [45], [46].

Information about a tick bite does not help determine the time of infection, since unnoticed tick bites lead to infection in about two thirds of cases [41], [47], [48]. For the classification of neuroborreliosis, therefore, the disease duration is increasingly used in addition to the clinical picture [49].

### 2.2 Neurological manifestations in adults

**Garin-Bujadoux-Bannwarth syndrome (meningoradiculoneuritis)** is the most common manifestation of acute Lyme borreliosis in adults in Europe after erythema migrans [41], [47], [50].

In Europe, isolated **meningitis** (without radicular symptoms) is mainly observed in children [12], [41], [43], [51], [52] .

The symptoms of **radiculitis** develop on average 4–6 weeks (maximum 1–18) after the tick bite or after the erythema migrans [41], [53]. Segmental pain occurs first, which intensifies at night and whose localisation can change. Often the pain is initially localised in the extremity where the tick bite or erythema migrans was first observed [41], [54]. The patient experiences pain that is burning, nagging, stabbing or tearing in nature and responds only slightly to conventional analgesics. It often peaks within a few hours or days. Three-quarters of patients develop neurological deficits after 1–4 weeks, and pareses are more frequent than sensory disorders [41], [53].

About 60% of patients with Bannwarth’s syndrome have **cranial nerve deficits**.

- All cranial nerves may be involved with the exception of the olfactory nerve.
- The facial nerve is affected in over 80% of cases where there is cranial nerve involvement [41], [55]. There is frequently a bilateral manifestation (approximately 1/3 of the cases) [41], [47], [56]. The sense of taste may not be affected. In unilateral cases, it can be difficult to differentiate from idiopathic facial nerve paresis; however, sometimes symptoms or anamnestic data (e.g. erythema migrans, radicular pain) can help to indicate Lyme neuroborreliosis. CSF testing can bring clarity here. In most cases, a complete regression is observed within 1–2 months regardless of the severity of the facial paralysis. Residual symptoms or partial recovery with facial synkinesia (pathological movement) are observed in about 5–10% of patients [56], [57], [58].
- Furthermore, Lyme neuroborreliosis may affect the abducens nerve and very rarely the vestibulocochlear nerve, the optic nerve (optic neuritis, papilloedema), the oculomotor system (NN III, IV), the trigeminal nerve and the caudal cranial nerves (NN IX–XII) [41], [47], [53], [59]. It is questionable whether isolated damage to the vestibulocochlear nerve occurs in the context of an acute Borrelia infection.

**Polyneuropathy/polyneuritis** is linked to a Borrelia infection in European patients only in association with acrodermatitis chronica atrophicans (ACA) in 48–64% of the cases [60], [61]. Isolated polyneuropathies/polyneuritis without other clear symptoms of Lyme borreliosis have been identified in 39–52% of American patients with Lyme borreliosis [62], [63]. However, in 284 US-American patients with etiologically unexplained polyneuropathy, Lyme borreliosis was identified as the cause of the polyneuropathy in only one case (0.3%) after diagnostic re-evaluation [64]. In contrast, few instances of distally symmetrical polyneuropathies or polyneuritis not associated with ACA have been identified in Europe. A causal link between neurological symptoms and a Borrelia infection cannot easily be made for patients with polyneuropathy/polyneuritis whose blood tests positive for Borrelia [65] since Borrelia-specific antibodies are found in approximately 5–20% of healthy individuals depending on the endemic region and age group [13], [15], [66]. Occupationally exposed risk groups, such as forestry workers, even have seroprevalences of over 50% [67]. In these cases, the probability of a causal link depends on whether further clinical symptoms of Lyme borreliosis are present and whether other common causes of polyneuritis have been identified.

**Involvement of the central nervous system** is rare and occurs in only around 4% of Lyme neuroborreliosis cases [41], [47]. Its onset is gradual and it is frequently chronic. The most common manifestation is myelitis with spastic atactic gait disturbance and bladder dysfunction [41], [50]. Symptoms can develop over days or several months. Some patients suffer from severe tetra- or paraparesis. Approximately 60% of patients with myelitis have additional signs of encephalitis and around 40% have cranial nerve involvement. Encephalitis has no clinical properties specific to the pathogen.

Encephalitis can lead to **psychiatric diseases** or **organic brain syndromes**. Cases of acute psychosis [47], [68], [69], [70], [71], [72] or Tourette’s syndrome [73] have been reported.

In very rare cases cerebral symptoms (e.g. ischemic stroke) can be caused by Borrelia-induced **vasculitis** [74], [75]. According to a non-systematic review, only 62 cases had been reported by 2015 [74]. Another very rare manifestation of Lyme borreliosis is **myositis**, for which only individual case reports exist [76], [77]. Clinical symptoms include focal pain and paresis.

### 2.3 Neurological manifestations in children 

In Europe, **Lymphocytic meningitis** (approximately 30%) and **facial paresis** (approximately 55%) are the most frequent manifestations of Lyme neuroborreliosis in children [12], [41], [51], [52], [78]. The symptoms of meningitis are often very discrete and can be overlooked in the absence of cranial nerve involvement [79]. The facial nerve and the nerves of the outer eye muscles are most frequently affected. In principle, all cranial nerves can be affected with the exception of the olfactory nerve. Radicular symptoms in the spinal nerves are rare. However, there are reported cases of early Lyme neuroborreliosis with myelitis [80], acute hemiparesis [81], opsoclonus-myoclonus syndrome [82] and ataxia [83]. **Late Lyme neuroborreliosis** is very rare in children. Clinical pictures include seizures, neurological deficits with paralysis and excretory disorders. Cognitive impairment and mood disorders can also occur [81].

### 2.4 Clinical course

**Early Lyme neuroborreliosis:** symptoms last for weeks to months [41], [47], [50].

- Presumably over 98% of cases [11], [43]
- Neurological symptoms appear several weeks to several months after a tick bite
- Typical manifestations: painful meningopolyradiculitis of the spinal nerves linked to a unilateral or bilateral facial paresis (Bannwarth’s syndrome); also meningitis in children
- Frequently: radicular pain

**Late Lyme neuroborreliosis** (also termed chronic **Lyme neuroborreliosis**): symptoms last for months to years [41], [47], [50].

- Presumably less than 2% of cases [11], [43]
- Neurological symptoms develop slowly over months to years
- Typical manifestations: encephalomyelitis with spastic atactic gait disturbance and bladder dysfunction
- Isolated meningitis is very rare
- Rarely any pain

Erythema migrans (EM) is indicated in the medical histories of 34–46% of patients with Lyme neuroborreliosis [41], [47], [50].

### 2.5 Symptoms that should lead to clarification of Lyme neuroborreliosis

(Hansen & Lebech 1992 [41]; Kaiser 1994 [50]; Oschmann et al. 1998 [47]) (Appendix 7 in Attachment 3 )

- Radiculitis of the spinal nerves (typical for early stages) (frequency 70–75%): initially severe, nocturnal, radicular or segmentally distributed pain, persisting without treatment for weeks, later development of paresis > paraesthesia
- Radiculitis of the cranial nerves II–XII (frequency 47–56%): facial nerve paresis most frequent (83–92%), bilateral in about one third; ocular muscle paresis (abducens nerve) (frequency 4–9%). Very rare (individual case reports): paresis of the oculomotor and trochlear nerves, optic neuritis, papilloedema, hearing loss, dizziness (vestibulocochlear nerve), paresis of the hypoglossal nerve
- Meningitis (in children [frequency of around 30%] more frequent than in adults [frequency 4–5%]): headache, meningism, photophobia, nausea, vomiting, fatigue, emotional instability; rarely chronic
- Neuritis of the peripheral nerves (extremely rare), probably only in the context of acrodermatitis chronica atrophicans/axonal polyneuropathy with predominantly sensory symptoms
- Encephalitis (mostly late Lyme neuroborreliosis) (older case series indicate a frequency of 4–5% for encephalomyelitis) [41], [47]: paresis, speech and language disorders, coordination disorders, occasional epileptic seizures; rarely organic brain syndrome with lack of concentration, loss of consciousness and hallucinations
- Myelitis (mostly late Lyme neuroborreliosis) (frequency similar to encephalitis, see above): transverse sensory dysfunction, central and peripheral paresis, voiding disorders; often in association with encephalitis
- Borrelia-induced cerebral vasculitis: rare, mainly ischemic events in different areas of the bloodstream with corresponding neurological symptoms [74]
- Borrelia-induced myositis: extremely rare [76], [77]

## 3 Diagnostic testing

### 3.1 Overview

**Figure 1:**
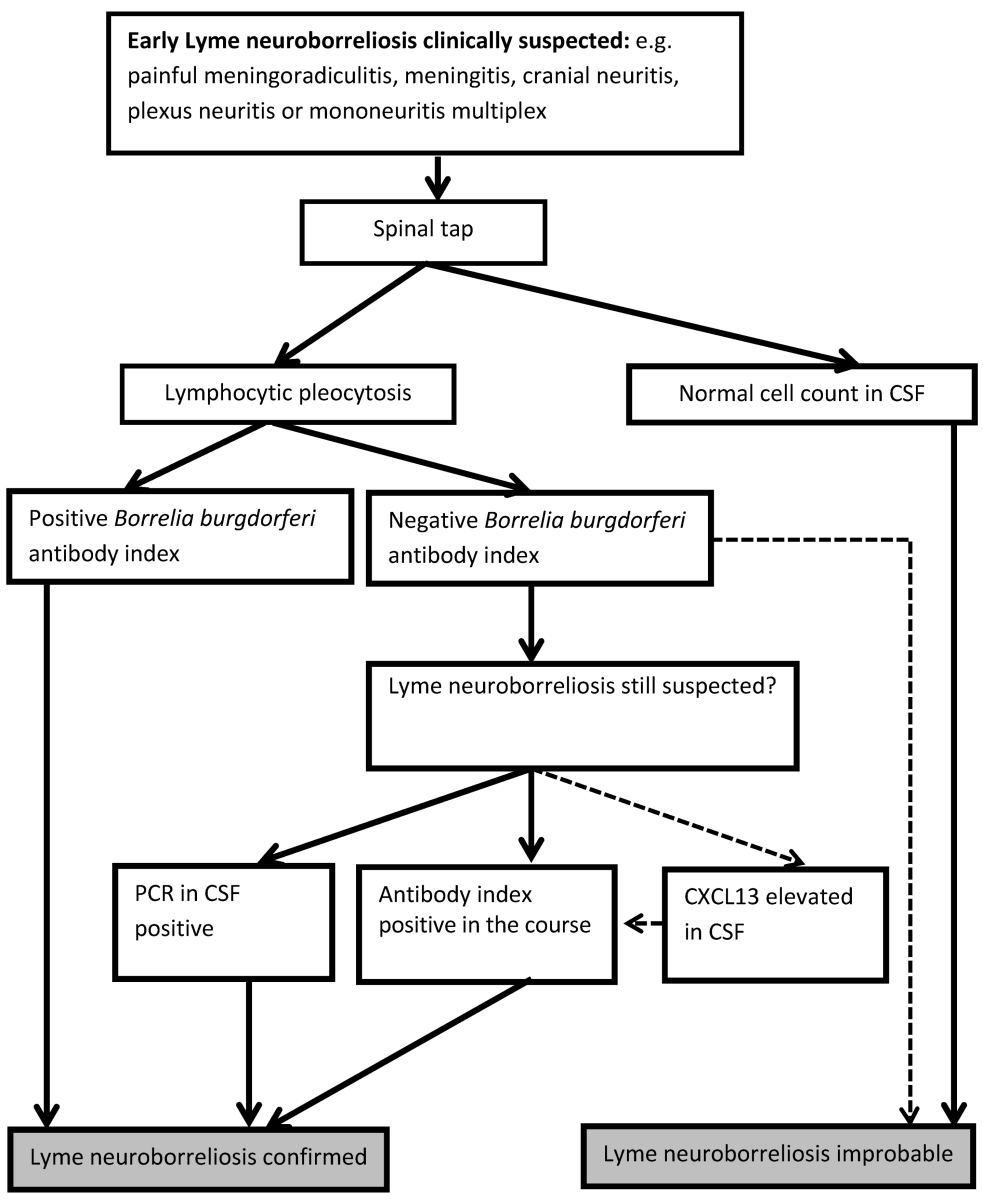
Diagnostic algorithm for early Lyme neuroborreliosis; modified according to [43]

**Figure 2:**
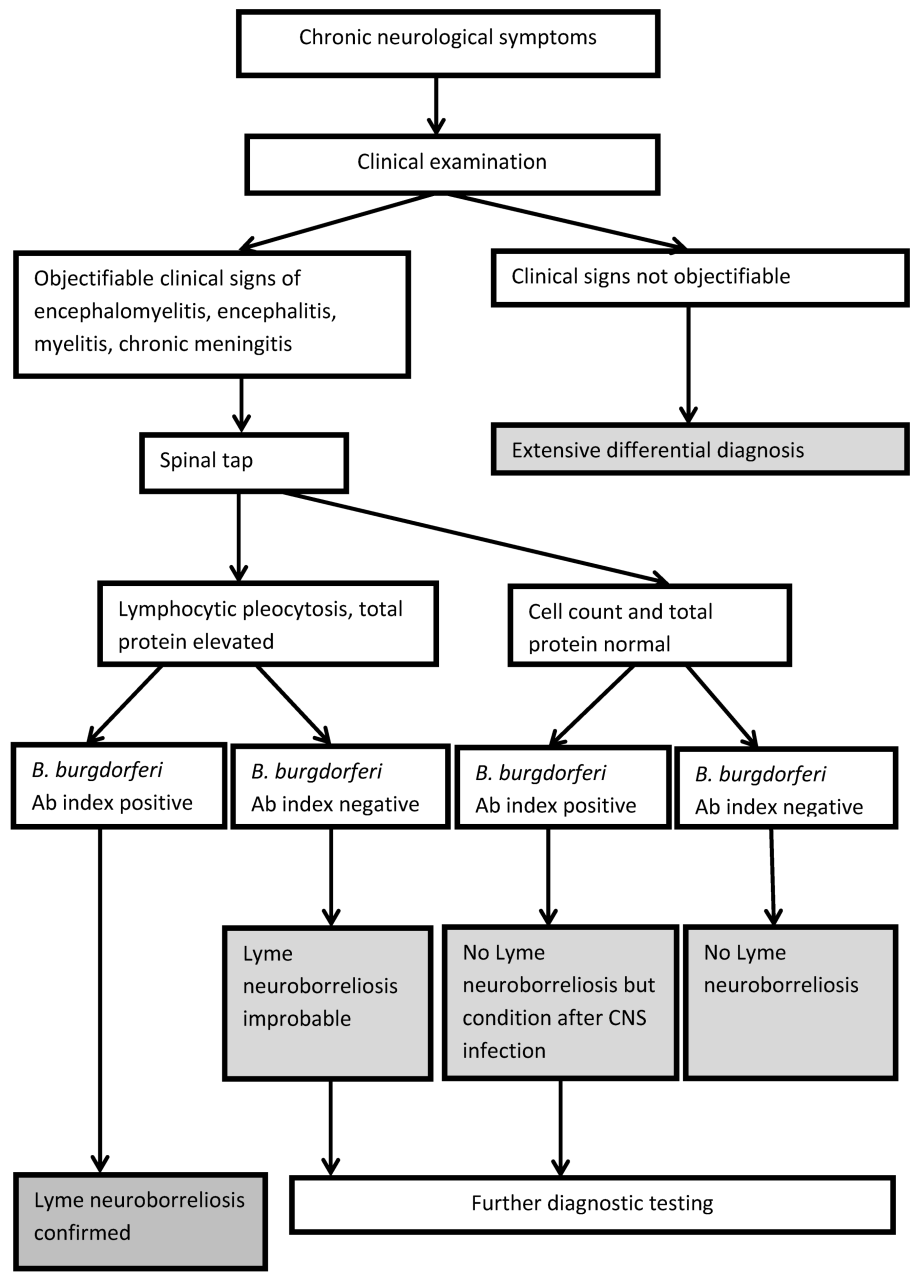
Diagnostic algorithm for late Lyme neuroborreliosis; modified according to [43]

Typical clinical symptoms are an indication of Lyme neuroborreliosis which must be underpinned by subsequent laboratory tests (serum and cerebrospinal fluid tests) [84], [85]. The diagnostic algorithm is illustrated in Figure 1 (Fig. 1) and Figure 2 (Fig. 2).

### 3.2 Inflammatory CSF changes

Inflammatory cerebrospinal fluid (CSF) changes (pleocytosis, blood-CSF barrier dysfunction and intrathecal immunoglobulin synthesis) can be expected for every Lyme neuroborreliosis (possible exceptions: very early stage of the disease or distal symmetric polyneuropathy).

The CSF typically exhibits **lymphocytic pleocytosis** with plasma cells, activated lymphocytes and a significant **increase in the total protein** or albumin ratio (blood brain barrier disorder) [50], [55] (Table 1 (Tab. 1)). The average cell count is between 170 and 220/µl with a range from 6 cells/µl [50] up to 1,100 cells/µl [47]. In addition, **intrathecal IgM synthesis** occurs in 80–100% of early manifestations and **IgG synthesis** in about 60% of patients [50], [86]. If intrathecal IgG synthesis is determined qualitatively by isoelectric focusing (detection of oligoclonal IgG bands), results will be positive in 70–80% of patients [50], [55]. Late Lyme neuroborreliosis has more frequent and higher **intrathecal IgG and IgA synthesis rates** than early Lyme neuroborreliosis (Table 1 (Tab. 1)).

**Lactate levels in the cerebrospinal fluid** are only slightly elevated in patients with Lyme neuroborreliosis. Only 5 out of 118 patients with early Lyme neuroborreliosis showed significantly elevated CSF-lactate levels (=3.5 mmol/l) and the mean CSF lactate concentration for the entire cohort was not elevated (2.1±0.6 mmol/l) (Table 1 (Tab. 1)) [55].

#### Recommendations

- If Lyme neuroborreliosis is clinically suspected, CSF and serum testing (simultaneous collection) should be performed. ↑↑
- The CSF analysis should include cytological, protein chemical and serological testing (AI calculation, see below). ↑↑ (both recommendations have a strong consensus 13/13)

### 3.3 Indirect pathogen detection in serum

#### 3.3.1 Serodiagnosis, antibody detection

In the case of early Lyme borreliosis, Borrelia-specific IgM antibodies can be detected starting week 3 p.i. and IgG antibodies starting week 6 p.i. [85]. However, the use of VlsE or C6 peptide as a test antigen means that IgG antibodies can now often be detected as early as IgM antibodies [85]. High IgG antibody concentrations are usually found in late manifestations of Lyme borreliosis (Table 2 (Tab. 2)) [85], [87]. A detectable humoral immune response does not always follow the usual course known from other infectious diseases: the measurable antibody response may (still) be absent in an early, localised manifestation (erythema migrans) [85]. At the same time, there may be no measurable IgM response, for example in the case of reinfections [85], [88]. In the context of very early antibiotic treatment, a measurable humoral immune reaction may also fail to appear [89]. On the other hand, the positive detection of borrelia-specific IgM and/or IgG antibodies alone is not an indication of an illness from *Borrelia burgdorferi* since

1. Borrelia infections with asymptomatic seroconversion can occur [23] and
2. elevated IgG and IgM antibody titres (in serum and/or cerebrospinal fluid) are not uncommon in healthy individuals for years following sufficiently treated Lyme borreliosis [90], [91], [92].

Borrelia serology is not suitable for monitoring antibiotic treatment of Lyme borreliosis and follow-up testing is therefore not recommended [85], [93].

The **serodiagnosis** of a systemic Borrelia infection includes a 2-step process: first a screening test (enzyme immunoassay) followed by a confirmation test (immunoblot) [85], [93].

Improvements in serodiagnosis include screening tests (ELISAs) that contain the preferably in vivo-expressed protein VIsE or the conserved immunodominant C6 region of this protein [85], [94]. In confirmation tests (immunoblot) for diagnosing acute Lyme neuroborreliosis, the recombinant line immunoblot was reported to have a significantly higher sensitivity than the conventional immunoblot combined with an equally high specificity (95%) [85], [95]. This was partly due to the new line immunoblot technique and partly to the widening of the antigen spectrum to include proteins only expressed by the Borrelia *in vivo* (in the host and not in the culture).

#### 3.3.2 Diagnostically relevant Borrelia antigens

*Borrelia burgdorferi* has a large number of immunologically relevant antigens which, depending on the stage, can be detected with varying degrees of sensitivity and which sometimes have different levels of specificity. Knowing these proteins is important when interpreting serological test results (detailed description in MiQ Lyme borreliosis [85]).

**Early immune response** (primarily IgM)

[95], [96], [97], [98]

- Flagellar protein (Flagellin, p41)
- OspC (associated with outer membrane)
- VlsE

**Late immune response** (primarily IgG)

[96], [99], [100]

- p83/100, p58, p43, p39, p30, p21, DbpA (Osp17) and p14 (generally reactive with around 80% of the sera [99])
- VlsE (detectable in more than 90% of the sera) [95]

**Non-specific antigens**

- Flagellin
- Heat shock proteins

##### Summary

- Positive antibody detection is not evidence for a clinical case of Lyme borreliosis.
- Negative antibody detection largely rules out Lyme borreliosis in immune-healthy patients with prolonged disease.
- An isolated positive IgM detection does not support a late manifestation of Lyme borreliosis.

##### Recommendations

- Serological testing should only be requested if there is sufficient clinical suspicion. ↑↑
- Testing should be done in stages (screening test and confirmation test) ↑↑ (consensus of both recommendations 10/13)

### 3.4 Intrathecal antibody synthesis – Borrelia-specific antibody index (AI)

#### 3.4.1 Overview

In most patients with Lyme neuroborreliosis the suspected clinical diagnosis can be confirmed by detecting Borrelia-specific intrathecal antibody synthesis related to inflammatory changes in their cerebrospinal fluid [85], [101], [102], [103]. The production of specific intrathecal antibodies is detected by determining the Borrelia-specific CSF/serum antibody index (Borrelia-specific AI) [41], [104], [105].

#### 3.4.2 Determination method

Methods used to **determine the AI** should take into account the blood/CSF barrier function, as otherwise false negative results may be produced [85]. The determination of the antibody index according to Reiber is a proven method that is recommended [85], [101], [106], [107]. The following formula is used to calculate the Borrelia-specific AI (the formula is illustrated with IgG. It can also be used to calculate IgM and IgA): Antibody index =


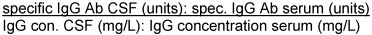


If intrathecal immunoglobulin synthesis is present in the Reiber diagram (i.e. the total IgG ratio relative to the albumin ratio is above the norm), the total IgG ratio must be replaced by the Q-Lim ratio (empirical limit value for the maximum IgG fraction derived from the serum as a function of the albumin ratio). In this case: Antibody index = 


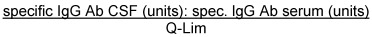


A value of =1.5 is recommended as the **cut-off** for a positive AI, unless otherwise evaluated [85], [86], [106], [108]; previously recommended higher limit values of 2.0 [109] are considered less sensitive when a reliable test performance can be ensured [86]. Quantitative measuring methods are usually used to determine the AI and are implemented in commercial, EDP-supported systems [85].

It is important to note that there can be considerable fluctuations in the determination of AI (both interrater-dependent for the same method and when comparing different methods) [86]. Hence antibody testing and AI determination should be conducted in accredited microbiology laboratories.

#### 3.4.3 AI throughout the course of the disease

Intrathecal *Borrelia burgdorferi*-specific antibody production develops in untreated patients from around week 2 and is detectable in over 99% of patients after 6–8 weeks [41], [101], [102], [103], [110]. During the course of the disease (short duration of disease), elevated CSF-Borrelia antibodies can sometimes be detected despite negative Borrelia antibodies in serum [55], [110], [111]. Conversely, Borrelia-specific AI can remain inconspicuous when the duration of the disease is short or in children with facial paresis [101], [110], [111]. Furthermore, very early antibiotic treatment can prevent the development of a measurable humoral immune response and cause the Borrelia-specific AI to remain negative [112].

After the Lyme neuroborreliosis has resolved, the Borrelia-specific AI can remain positive for months or years in symptom-free patients [50], [113], [114]. Borrelia-specific AI is not suitable for monitoring treatment success and should be interpreted in relation to clinical symptoms and inflammatory changes in cerebrospinal fluid (pleocytosis, blood CSF barrier disorder).

##### Summary

- A clinically suspected diagnosis of Lyme neuroborreliosis can be confirmed by the detection of intrathecal Borrelia-specific antibody synthesis (positive Borrelia-specific antibody index [AI]) in connection with inflammatory changes in cerebrospinal fluid.
- Intrathecal Borrelia-specific antibody synthesis starts in about the second week of the disease and is detectable after 6–8 weeks in over 99% of patients.
- A Borrelia-specific AI without accompanying inflammatory changes in CSF may remain positive for years after Lyme neuroborreliosis has resolved.

##### Recommendations

- The Borrelia-specific AI should be determined if Lyme neuroborreliosis is suspected. ↑↑
- The Borrelia-specific AI should not be used to monitor treatment success. ↑↑ (strong consensus for both recommendations 13/13)

### 3.5 Chemokine CXCL13

In recent years, the chemokine CXCL13 has been shown to increase significantly in the CSF of almost all patients with acute Lyme neuroborreliosis – even before a specific antibody response is generated. Once antibiotics are administered, chemokine levels immediately drop very quickly, long before CSF pleocytosis regresses [115], [116], [117]. A prospective study of 179 patients with suspected Lyme neuroborreliosis showed a sensitivity/specificity of 100%/99% and positive and negative predictive values of 88% and 100% respectively [118]. Hence, the parameter can be helpful in ambiguous cases of very early Lyme neuroborreliosis [43], [85]. It should be noted that CXCL13 is not specific to Lyme neuroborreliosis; increased CSF values have also been found with neurosyphilis, tubercular meningitis and CNS lymphomas [116], [119], [120], [121], [122]. Furthermore, determination – including cut-off – has yet to be generally standardised.

#### Summary

- CXCL13 levels in the cerebrospinal fluid correlate with the “disease activity” (indication of existing infection) of Lyme neuroborreliosis and can be diagnostically helpful in individual cases.
- CXCL13 determination has yet to be generally standardised.
- Elevated CXCL13 values in CSF also occur in conjunction with other diseases.

#### Recommendation

- CXCL13 can be determined in CSF when early Lyme neuroborreliosis is clinically suspected and the CSF cell count and/or Borrelia-specific AI are (still) inconspicuous. ↔ (strong consensus 12/13)

### 3.6 Direct detection of the pathogen using molecular biological detection methods and culture

In exceptional cases (e.g. immunosuppressed patients (e.g. insufficient antibody production due to primary immunodeficiency or B-cell depletion)), a Borrelia infection can be underpinned by pathogen detection in CSF [85], [123]. However, for acute Lyme neuroborreliosis, the sensitivity of the pathogen detection in CSF through culture or a PCR test is only 10–30% [85], [87]. Pathogen detection is expected to have a higher sensitivity when the duration of the disease is short (where patients may still be seronegative) than in prolonged cases. For example, 50% of patients with acute Lyme neuroborreliosis tested positive with PCR compared to only 13% of patients with a prolonged course of the disease [124]. Detection in the cerebrospinal fluid using a PCR test is generally preferred because results can be provided faster than for cultures. If the results are positive, a species diagnosis should be made by analysing the PCR products. Detection of the pathogen in blood is not recommended because this method is even less sensitive [85]. The specificity of the PCR test depends to a large extent on the quality of the laboratory performing it. Therefore, the investigation should be explicitly limited to special, designated reference laboratories, especially as further molecular biological confirmation tests are required when the results are positive [85]. In every case, the PCR result must be interpreted in relation to the symptoms and the serology results. For example, positive PCR test results for patients with a prolonged disease and negative serology are very likely to be false positive [85].

#### Recommendations for direct detection using molecular biological methods and culture

- Molecular biological detection and direct detection in culture using cerebrospinal fluid should only be employed for the differential diagnosis of ambiguous cases (e.g. insufficient antibody production when there is a primary immunodeficiency or B-cell depletion). ↑ (consensus 11/13)
- Molecular biological detection and the cultivation of *Borrelia burgdorferi* sensu lato should be restricted to specialist laboratories. ↑ (strong consensus 13/13)
- Positive culture results should be confirmed using suitable molecular biological methods. ↑↑ (strong consensus 13/13)
- Molecular biological detection or direct detection in culture should not be used as a screening test if Lyme borreliosis is suspected. ↑↑ (strong consensus 13/13)
- Lyme neuroborreliosis should not be ruled out if the results of the molecular biological test or culture are negative for the pathogen. ↓↓ (strong consensus 13/13)
- Positive results for molecular biological detection or detection in culture should be confirmed by further molecular biological testing methods and the detected genospecies should be reported in the findings. ↑↑ (strong consensus 13/13)
- No additional treatment should be carried out if detection results are positive following antibiotic treatment conducted in accordance with the guidelines and without typical clinical manifestation. ↓↓ (strong consensus 13/13)

### 3.7 Routine laboratory parameters in blood

In the routine laboratory, patients with Lyme neuroborreliosis have normal or slightly elevated values for ESR, CRP, leukocytes and transaminases that indicate a systemic infection (Table 3 (Tab. 3)). When diagnosing Lyme neuroborreliosis, the routine laboratory results only play a role in differential diagnosis.

### 3.8 Diagnostic imaging – MRI

Magnetic resonance imaging (MRI), including MR angiography, is indispensable in diagnosing Borrelia-induced vasculitis; MR tomography can detect both cerebral ischemia and intracranial vascular stenosis [47], [74], [75]. Inflammatory lesions that show gadolinium enhancement in MRI have been detected in individual cases of encephalomyelitic manifestations [47], [43]. However, there are no controlled studies on the diagnostic value of MRI in Lyme neuroborreliosis. In most cases of early Lyme neuroborreliosis, inconspicuous findings are expected due to the very rare involvement of the brain and spinal cord; here, MRI is primarily used for differential diagnosis.

### 3.9 Testing

The following tests should be conducted if Lyme neuroborreliosis is clinically suspected (for symptoms see Section 2.4):

- Targeted anamnesis with questions related to tick bites, visits to endemic areas, early symptoms (erythema migrans, multiple erythema migrantia, Borrelia lymphocytoma [lymphadenosis cutis benigna], general symptoms), psychosocial anamnesis if necessary
- Neurological state, inspection of the skin (erythema migrans may still be detectable at the time of neurological symptoms)
- Basic lab tests with inflammation parameters
- CSF analysis: cell count, differential cell count, total protein, immunoglobulins, lactate
- Borrelia serology including Borrelia-specific CSF/serum antibody index (AI)

### 3.10 Diagnostic criteria for Lyme neuroborreliosis

Depending on the constellation of the clinical findings and laboratory data, the diagnosis of Lyme neuroborreliosis can be classified as possible, probable and definite (see below) [84], [125].

#### Possible neuroborreliosis

- Typical clinical picture (cranial nerve deficits, meningitis/meningoradiculitis, focal neurological deficits; cf. Section 2.4)
- Borrelia-specific IgG and/or IgM antibodies in serum (*The serology may [still] be negative in very early stages of the disease*)
- CSF findings not available/spinal tap not performed
- Differentiation from other causes

##### Probable Lyme neuroborreliosis

As with “possible Lyme neuroborreliosis”, however additionally

- Inflammatory cerebrospinal fluid syndrome with lymphocytic pleocytosis, blood-CSF barrier dysfunction and intrathecal immunoglobulin synthesis

##### Definite Lyme neuroborreliosis

As with “probable Lyme neuroborreliosis”, however additionally

- Intrathecal synthesis of Borrelia-specific antibodies (positive IgG and/or IgM antibody index) in CSF or
- Positive culture or nucleic acid detection (PCR) in cerebrospinal fluid

### 3.11 Testing methods not suitable for diagnosing Lyme neuroborreliosis

There are no prospective controlled studies available for the following methods that would prove useful for the diagnosis of Lyme neuroborreliosis.

Therefore, these methods should not be used in diagnosing Lyme neuroborreliosis ↓↓ (consensus 10/12) [85]:

- Antigen detection in bodily fluids
- PCR in serum and urine
- Lymphocyte transformation tests (LTT) [126], [127], [128], [129]
- Enzyme-linked immunospot assay (ELISPOT) [130]
- “Xenodiagnosis” – hard tick larvae suck blood from suspected Lyme borreliosis patients; the larvae are subsequently tested for Borrelia [131], [132]
- Visual contrast sensitivity test (VCS test or grey scale test): By measuring the detection of shades of gray, a lipophilic neurotoxin from Borrelia is to be detected indirectly [133]
- Detection of so-called L forms or spheroplasts [134]
- Detection of immunocomplexes as markers of disease activity
- CD57 positive/CD3 negative lymphocyte subpopulation [135]
- Commercially available serological rapid tests (insufficient sensitivity (18–32%) [136]

**Note:** The DBG and the patient organisations BFBD, BZK and OnLyme-Aktion.org have issued dissenting opinions on this topic, which are published in an appendix (Attachment 1) to the guideline report (Attachment 2).

## 4 Chronic and atypical symptoms linked to Lyme neuroborreliosis

### 4.1 Introduction

In addition to the confirmed early and late manifestations of Lyme neuroborreliosis (such as radiculitis, meningitis or encephalomyelitis and/or their clinical residuals), there is a broad range of persisting symptoms in which a causal link to Lyme neuroborreliosis is suspected without an inflammatory-infectious process being detectable on the basis of generally accepted criteria [43], [137], [138], [139], [140], [141], [142]. The terms used for these chronic symptoms include “post-treatment Lyme disease syndrome” (PTLDS), “(post-)Lyme encephalopathy” or simply “chronic Lyme (neuro)borreliosis” and are often used with no clear delineation between them. Characteristic for all three illnesses is that general symptoms predominate. It is questionable whether it makes sense to administer repeated doses of antibiotics as no studies have provided reliable evidence for this [142], [143].

The frequency and range of persistent symptoms following antibiotic treatment in patients with Lyme neuroborreliosis have been systematically investigated [5]. Forty-four studies published between 1986 and 2014 were identified (8 RCTs, 17 cohort studies, 2 case-control studies and 17 case series), of which 38 studies (n=1,469 patients) reported patients with residual symptoms. A total of 28% of patients (95% CI 23–34%, n=34 studies) had persistent or residual symptoms. In studies in which the inclusion criteria (case definition) were a “probable or definite” case of Lyme neuroborreliosis (inflammatory changes in CSF), the prevalence of persistent symptoms was 24% (95% CI 0.16–0.33; n=547) – significantly lower (p=0.0048) than with patients whose inclusion criterion was only a “possible” case of Lyme neuroborreliosis (CSF findings inconspicuous or unavailable) (31% [95% CI 0.25–0.37]; n=922). Furthermore, the type of persistent symptoms also differed between the two patient groups. The non-specific complaints typically reported for PTLDS (see Section 4.3) were statistically more prevalent in patients with “possible” Lyme neuroborreliosis than in patients with “probable/definitive” Lyme neuroborreliosis: fatigue (5.13% vs. 0%), cognitive disorders (16.67% vs. 1.6%), general pain (18.75% vs. 2.77%), headaches (8.33% vs. 1.75%) (Table 4 (Tab. 4)). Even though a study bias or the presence of different disease stages in the cohorts studied cannot be definitively ruled out, the authors conclude that the significant prevalence of persistent atypical symptoms for Lyme neuroborreliosis, as reported in the studies, is largely due to study artefacts as a result of blurred case definitions.

### 4.2 Presumptive chronic Lyme neuroborreliosis

#### 4.2.1 Introduction

The terms “chronic Lyme borreliosis” or “chronic Lyme neuroborreliosis” are confusingly used in an overlapping sense with very different meanings and correspondingly different therapeutic consequences. They mostly refer to non-specific symptoms such as fatigue, musculoskeletal pain, cognitive disorders and depression [140], [141], [142], [143], [144], [145], [146], [147], [148]. In terms of the pathophysiology of presumptive “chronic Lyme disease” or “chronic Lyme neuroborreliosis”, current systematic reviews have not found a scientific basis for the assumption of a persistent latent infection caused by *Borrelia burgdorferi* [140] or its morphological variants [134]. Likewise, no evidence has been found for chronic co-infections transmitted by tick bites in patients with non-specific symptoms [149]. Feder et al. have described 4 clinical categories to which patients with presumptive “chronic Lyme borreliosis” can be assigned (for complete criteria according to Feder see Appendix 1 in Attachment 3 ) [142].

- **Category 1** includes patients with symptoms of an unknown cause without evidence of an infection with *Borrelia burgdorferi*.
- **Category 2** includes patients with symptoms of a known, well-defined illness without evidence of an infection with *Borrelia burgdorferi*. Here the original diagnosis is presumed to be false.
- **Category 3** describes patients with symptoms of an unknown cause when the Borrelia serology tests positive but there is no objective clinical finding of Lyme borreliosis.
- **Category 4** refers to patients with PTLDS-like symptoms (PTLDS see Section 4.3 and Appendix 2 in Attachment 3 ).

#### 4.2.2 Present study situation

Older studies, in which patients with presumptive “chronic Lyme borreliosis” were re-evaluated at specialised academic centres, primarily featured category 1 and 2 illnesses according to Feder [150], [151], [152]. Later studies on this topic examined 240 US-American patients [153], 29 Norwegian patients [154], 95 German patients [155] and 200 Dutch patients [156]. In summary, Lyme borreliosis was confirmed in a smaller percentage of patients (13–24%). PTLDS was presumed in 6–20% of patients, with no proven causal link to Lyme borreliosis and no indication for antibiotic treatment (see above). A diagnosis remained undetermined in 18–52% of cases. All in all, these studies suggest that if “chronic Lyme borreliosis” is suspected, it is imperative that an intensive differential diagnosis of both organic and psychosocial disease factors be conducted [156], [157]. Furthermore, in light of the very broad distribution of the study results cited here, further research is regarded as necessary.

#### 4.2.3 Practical approach

There is no rationale behind administering antibiotics to categories 1 and 2 according to Feder. Based on current data (see Section 4.3), antibiotic treatment of category 4 is also not indicated. In patients with category 3 symptoms according to Feder [142] probatory (oral) antibiotic treatment may be considered. However, these patients should be advised that the diagnosis of Lyme borreliosis is very uncertain in their situation, as the predictive value of Borrelia serology is very low when symptoms are non-specific [158], [159] and temporary “treatment effects” may be caused by both the placebo effect [160] and by the anti-inflammatory side effects of antibiotics [161], [162], [163].

#### Summary

- None of the 4 categories according to Feder [142] correspond to a disease entity.

#### Recommendations

- As with Lyme neuroborreliosis, patients in categories 1, 2 and 4 according to Feder [142] should not be treated with antibiotics. Instead a differential diagnosis should be performed based on the symptoms and treatment should be prescribed based on the primary symptoms. ↑↑ (consensus 9/11)
- In exceptional cases, a single round of antibiotics lasting 14–21 days may be considered for category 3 patients following a detailed differential diagnosis and taking into account the fact that it is an unconfirmed diagnosis. ↔ (10/14 majority consensus)

### 4.3 Symptoms following treatment: “Post-Treatment Lyme Disease Syndrome” (PTLDS)

#### 4.3.1 Diagnostic criteria

PTLDS is a syndrome that has not yet been scientifically defined and therefore not uniformly accepted. It is to be diagnostically differentiated from confirmed late manifestations of Lyme borreliosis, symptoms caused by the persistence of reproducing pathogens, and symptoms caused by partial recovery.

In the case of Lyme neuroborreliosis, objective neurological deficits and inflammatory changes in the cerebrospinal fluid usually respond well to antibiotic treatment [41], [43], [137], [138], [139], [140]. However, some patients are reported to have developed non-specific symptoms of fatigue, paraesthesia, muscle and joint pain as well as concentration and memory issues despite antibiotic treatment [164], [165], [166], [167]. If the non-specific symptoms last more than 6 months, some authors refer to this as post-treatment Lyme disease syndrome (PTLDS) [27], [142]. Predictors for the development of fatigue 30 months after treatment have been described as the delayed onset of antibiotic treatment, severe neurological symptoms before treatment, and incomplete regression of neurological symptoms 4 months after treatment [166].

In 2006, the Infectious Diseases Society of America (IDSA) proposed the following diagnostic criteria for PTLDS [27]. The main criteria for this definition are: a previous, confirmed case of Lyme borreliosis which has improved or stabilised under a generally accepted antibiotic treatment regimen, and the occurrence of subjective symptoms within 6 months after diagnosis of Lyme borreliosis without any indication of another aetiology despite thorough differential diagnosis, which persist for at least 6 months after completion of the antibiotic treatment (see Appendix 2 in Attachment 3 for the full definition). So far, these criteria have seldom been used in clinical studies. This would require the establishment of practical and reliable tools to assess these subjective symptoms and their influence on the patient’s quality of life and professional and general performance [139], [168].

#### 4.3.2 Frequency

In a non-systematic review, it was reported that 0–20% of patients being treated for Lyme borreliosis with antibiotics had symptoms of so-called PTLDS; after treatment of Lyme neuroborreliosis the percentage was between 5 and 54% [43].

#### 4.3.3 Subjective symptoms in case-control studies

The frequency of subjective symptoms was investigated in case-control studies comparing cohorts of patients that previously had Lyme borreliosis and persons that did not have Lyme borreliosis. Since PTLDS-like symptoms are non-specific and also common among the general population [169], [170], classifying them as Lyme neuroborreliosis in the sense of a causal secondary disease is very problematic. This problem is also reflected in very heterogeneous data: compared to control subjects, German adults as well as Swedish and US-American children did not exhibit an increased frequency of non-specific symptoms at long-term follow up after the treatment of Lyme neuroborreliosis [171], [172], [173], [174]. The same applied to European patients following the treatment of erythema migrans [175] and American patients after various manifestations of Lyme borreliosis [176], [177]. Other case-control studies found a significant accumulation of non-specific symptoms in children and adults after the treatment of Lyme neuroborreliosis [148], [178], [179] or after any manifestation of Lyme borreliosis [180], [181], [182]. A meta-analysis examined five of the studies cited above [173], [177], [179], [180], [181] and concluded that there is an overriding link between the chronic symptoms of PTLDS and a previous case of Lyme borreliosis [183]. This meta-analysis is countered by the fact that it includes various retrospective studies whose diagnostic criteria and antibiotic treatment no longer meet current standards [184].

According to another study, fatigue and depression lead to physical and psychological impairment in patients with PTLDS-like symptoms [185], which is why the authors recommend targeted symptomatic treatment of these primary symptoms.

#### 4.3.4 Neuropsychological symptoms in case-control studies

The current study situation is contradictory in terms of the frequency of neuropsychological symptoms. In addition to subjective symptoms, objective neuropsychological impairments (verbal and visual memory, attention, executive functions) ≥30 months after treatment of Lyme neuroborreliosis are described as possible consequences of the disease [186], [187]. However, these reports could not be confirmed by another study [174] nor in children who had previously had Lyme neuroborreliosis (facial paresis) [179]. In addition, further studies – at least in subgroups – revealed limited memory performance, mainly in verbal tasks, compared to healthy controls or patients who had fully recovered [180], [188], [189], [190], [191], [192], [193]. However, there are also results that contradict these studies [176], [181], [194], [195].

#### 4.3.5 Studies on antibiotic treatment

Three randomised, placebo-controlled studies have examined the therapeutic benefit of antibiotic treatment over 28 to 70 days in patients with PTLDS [193], [195], [196], [197]. None of the studies identified a sustained improvement in neuropsychological performance. 

The most extensive of the three studies (N=129), in which antibiotics were administered for the longest period of time (2 g/d of ceftriaxone for 30 days followed by 200 mg/d of doxycycline for 60 days), was negative for all endpoints (especially health-related quality of life and cognitive functions) [195], [196].

A study by Fallon et al. [193] (N=37, 2 g/d of ceftriaxone over 70 days) found a temporary improvement in cognitive performance after 12 weeks, but this was not confirmed after 24 weeks. There was no significant difference between the fatigue scales of the placebo and verum groups.

A study by Krupp et al. (N=55, 2 g/d of ceftriaxone over 28 days) showed a slight but significant improvement in the fatigue score of the verum group compared to the placebo group after 6 months [197]. Critics state that

1. the effect is very marginal (score improvement in FSS-11: 22% versus 9% verum/placebo [p<0.01]);
2. patients in the verum group still had very severe fatigue (mean FSS-11=4.4) even after treatment, so that they continued to meet the inclusion criteria of the study;
3. the results of a second fatigue scale (Fatigue-VAS) were insignificant and
4. the improvement was not perceived by the patients themselves on a scale of health-related quality of life (first question of SF-36) [198].

In light of the very low effects and based on the fact that there was a critically high number of protocol drop-outs (33% of placebo patients) in the study [199], the validity of this study is questionable from a methodological point of view [198]. In addition, the study results are contradicted by two negative studies [193], [195], [196].

In all three studies, side effects – some of which are life-threatening – are reported at a rate of 25%–43%. Based on a risk-benefit analysis, none of the three author groups recommends treating so-called PTLDS with any of the investigated antibiotic regimens [193], [195], [196], [197].

#### 4.3.6 Pathophysiology

The pathophysiology of so-called PTLDS is unclear. An autoimmune process has not been proven [142], [200], [201]. In light of the negative or marginal effects of repeated antibiotic treatments (see Section 5), a chronic infection is unlikely. This assumption is further supported by the following arguments [142]: no accompanying, objective clinical signs of the disease and/or inflammation with progression [196], [202], persistence of symptoms irrespective of a positive Borrelia serology [196], [202], [203], no pathogen detection by culture and/or PCR [196], [204], no proven resistance of *Borrelia burgdorferi *sensu lato to the commonly used antibiotics [138], [205].

#### Summary

- Due to inconsistent data, so-called PTLDS cannot be defined as a disease entity.
- There are no controlled studies on the frequency of so-called PTLDS.
- The data refute the assumption of a chronic infection with *Borrelia burgdorferi* or an autoimmune process in patients with symptoms of so-called PTLDS (strong consensus for all 3 statements of 13/13).

#### Recommendations

- In the case of PTLDS-like symptoms, symptom-based differential diagnosis and treatment should be carried out. ↑↑ (consensus 11/13)
- If a so-called PTLDS is assumed, antibiotic treatment should not be prescribed. ↓↓ (consensus 11/12)

#### Further guidelines exist for PTLDS-like symptoms

- DEGAM S3 guideline on fatigue, AWMF Register No. 053-002 [206]
- DIVS S3 guideline on fibromyalgia syndrome, AWMF Register No. 041-004 [207]
- DEGAM S1 guideline on chronic pain, AWMF Register No. 053-036 [208]
- National Disease Management Guideline (S3) “Unipolar Depression”, AWMF Register No. nvl-005 [209]
- DGN guideline (S2e) on the diagnosis and treatment of memory disorders, AWMF Register No. 030-124 [210]
- DGPM S3 Guideline “Management of Patients with Non-specific, Functional and Somatoform Physical Complaints”, AWMF Register No. 051-001 [211]

### 4.4 Lyme encephalopathy

The term “Lyme encephalopathy” was originally coined in the 1980s when some clinical manifestations of Lyme disease were first described. At the time, patients frequently suffered from an undiagnosed, detectably active Borrelia infection (e.g. arthritis or ACA) for months or even years and reported cognitive complaints including memory disorders, which usually regressed after antibiotic treatment [189], [212], [213], [214]. In these case series, encephalitis was identified in only a small subset of patients who exhibited focal neurological deficits, abnormalities in cerebrospinal fluid or in imaging [213]. The majority of these patients suffered from “toxic-metabolic” encephalopathy as described in systemic (non-neurological) infections or inflammatory diseases (sepsis, pneumonia, urinary tract infections, active rheumatoid arthritis, etc.) [201], [215], [216]. Since this is a non-specific reaction of the brain to a systemic inflammatory process, the term “Lyme encephalopathy” should only be used in connection with the historical publications cited above.

Other authors use the term Lyme encephalopathy in connection with cognitive complaints in PTLDS patients [185], [193]. Since it is not possible to differentiate the term “Lyme encephalopathy” from its more historical use in the 1980s as outlined above, this designation should currently not be used as a diagnosis or syndrome designation.

#### Recommendation

- The term Lyme encephalopathy should not be used due to its unclear definition and contradictory use in diagnoses. ↓↓ (strong consensus 13/13)

## 5 Treating Lyme neuroborreliosis

### 5.1 Introduction

A current systematic review [4], [217] found that there is limited evidence concerning drug treatment for Lyme neuroborreliosis. Eight randomised controlled trials (RCT) and eight non-randomised studies (NRS) were selected for the evaluation after screening 5,779 reports from three databases. The authors state that the conclusions for medical practice must be weighed against the low number of studies – some of which had small cohorts – and the relevant risk for diverse study biases (Appendix 8 in Attachment 3) [4].

Only three studies examined patients who did not undergo antibiotic treatment [218], [219], [220]. Two studies compared these with patients who received antibiotic treatment [219], [220]. The studies used heterogeneous methods and produced contradictory findings with a low degree of precision. Therefore, a meta-analysis of these data is not justified (Appendix 8 in Attachment 3) [4]. Nevertheless, when the benefits are weighed against the risks, antibiotic treatment is indicated beyond doubt, especially as this can accelerate the regression of symptoms and counteract the development of late manifestations [41], [42], [174], [221], [222].

### 5.2 Early Lyme neuroborreliosis

#### 5.2.1 Duration of treatment

Eight RCTs and eight prospective cohort studies predominantly examined patients with early Lyme neuroborreliosis. The duration of antibiotic treatment in the RCTs was 14–21 days (with one exception of 100 days [223]. Treatment duration in the NRSs varied from 10 to 30 days, if specified at all. No studies compared different treatment durations. The treatment effect on the primary endpoint (neurological residual symptoms) varied considerably in both the 8 RCTs (10–66%) and the 2 prospective cohort studies (7–44%) (Appendix 3 in Attachment 3). The main reasons for this broad range of results are non-standardised survey methods (neurological status, score system, patient self-assessment) as well as different assessment timeframes, including wide-ranging assessment times within the individual studies themselves (3 RCTs: 3–12 months; 3 RCTs 12 months; 2 RCTS >3 months) [4].

When comparing different treatment durations, there is, in fact, indirect evidence based on a prospective controlled study that examined 152 patients with disseminated Lyme disease (80% with predominantly early Lyme neuroborreliosis [43% confirmed, 37% possible]) [223]. Patients were initially treated with 2 g of ceftriaxone i.v. per day for 3 weeks. This was followed by further treatment carried out on a randomised basis for 100 days during which the patient received either 1 g of amoxicillin p.o. per day or a placebo. After 1 year, about 90% of the patients in both groups exhibited excellent or very good results. This study therefore is an indication that there is no benefit to extending treatment beyond 3 weeks (Class Ib). A lack of study evidence for longer treatment times and the existence of a controlled study with indirect evidence reveal that there is no scientific basis for deviating from the previously recommended treatment duration of 14 days [109], [224] for early Lyme neuroborreliosis.

#### 5.2.2 Choice of antibiotics and side effects

Due to their good CSF penetration, controlled clinical trials have evaluated beta-lactam antibiotics (penicillin G, ceftriaxone and cefotaxime) and doxycycline in treating Lyme neuroborreliosis. According to a meta-analysis, the orally administered doxycycline and the intravenously administered beta-lactam antibiotics show no statistically significant difference with regard to the regression of neurological symptoms after an investigation period of 4–12 months (RR 1.27, 95% confidence interval 0.98–1.63, P=0.07) and after more than 12 months (RR 0.98, 95% confidence interval 0.68–1.42, P=0.93) and are therefore of similar efficacy (Class Ia) [4]. These findings confirm an earlier meta-analysis conducted by American authors [225]. Secondary endpoints, such as quality of life and fatigue, were investigated in an RCT follow-up study [178], [226]. No significant difference was found after 30 months between patients treated with beta-lactam antibiotics and those receiving doxycycline (Class Ib). Two RCTs showed that these two antibiotic treatment regimens did not differ with regard to cerebrospinal fluid pleocytosis [4], [221], [226] (Class Ib). Based on two RCTs [223], [227] there was also no statistically significant difference (RR 0.82, 95% CI 0.54–1.25, P=0.35) (Class Ia) in terms of reported side effects. The following side effects were reported: diarrhoea, nausea, constipation, redness of the skin, dizziness, and thrombophlebitis. Severe side effects such as cholecystitis, stomatitis, allergic reactions and duodenal ulcers were not reported frequently enough in the studies to make valid comparisons (Appendices 4 and 5 in Attachment 3) [4].

There are currently no studies that investigate doxycycline doses higher than 200 mg/d, which is why no statement can be made in regard to higher dosages of doxycycline [4].

A comparison of cefotaxime and penicillin in two RCTs [228], [229] revealed cefotaxime had a significant advantage in terms of fewer neurological residual symptoms after 4–12 months (RR 1.81, 95% CI 1.10–2.97, P=0.02). In contrast, patients treated with penicillin had significantly fewer side effects (RR 0.54, 95% CI 0.35–0.83, P=0.005). Mild diarrhoea and Herxheimer-like reactions were found to be the most common side effects (41%) (Appendices 4 and 5 in Attachment 3). Since serious side effects such as colitis, shock and allergic reactions (3%) were not reported enough for comparative analysis [229] and both studies are also subject to a significant risk of bias (Appendix 8 in Attachment 3), no recommendation can be derived from these data with regard to a preference of either substance over the other [4].

There is currently no valid analysable study data on the efficacy of antibiotic combination treatments and no study data are available on the efficacy of chloroquine, carbapenems and metronidazole [4].

#### 5.2.3 Course following antibiotic treatment

Most studies report a significant improvement in neurological symptoms several weeks to a few months following antibiotic treatment lasting 10–14 days. In a prospective study of 77 patients with Bannwarth’s syndrome, 88% of patients had good results 12 months after antibiotic treatment (Class IIa) [230]. The reported frequency of residual neurological symptoms is consistent with previous cohort studies in which 78/86 (90.6%) of patients were symptom-free 3 months after antibiotic treatment [42], and 178/187 patients exhibited very good results after 4–72 (median 33) months [41]. Another cohort study found that the daily activities of 100/114 (88%) patients with predominantly early Lyme neuroborreliosis were not impaired after an observation period of 5 years [164]. A systematic review examined the residual symptoms in 687 patients with Lyme neuroborreliosis confirmed through the diagnostic testing of CSF (probable/definitie Lyme neuroborreliosis) [5]. The following rates of neurological residual symptoms were found after antibiotic treatment: sensory disorders 5.24%; cranial nerve paresis 3.6%; extremity paresis 2.33%, pain 2.77%; unsteady gait/dizziness/ataxia 2.62% (Appendix 6 in Attachment 3).

### 5.3 Late Lyme neuroborreliosis

There are no controlled studies that explicitly investigate antibiotic treatment of late manifestations of Lyme neuroborreliosis (myelitis, encephalitis, encephalomyelitis). In the 16 systematically analysed treatment studies (RCTs and cohort studies) [4] only 15 patients reportedly had late Lyme neuroborreliosis (Appendix 3 in Attachment 3 ). A separate evaluation of this form of manifestation is not possible due to a lack of data in the primary studies. However, neurological residual symptoms appear to occur more frequently than with early Lyme neuroborreliosis (Class III). In a case series examining 15 patients, only 3 patients (20%) were symptom-free 6 months after antibiotic treatment [42]. In another cohort study, 8/8 patients with encephalomyelitis caused by late Lyme neuroborreliosis had neurological residuals after 4–72 months (median 33), whereby 5/8 (62%) had severely disabling residual symptoms [41].

The controlled studies and cohort studies [4] as well as the larger case series [41], [42] have shown no evidence of treatment failure when beta-lactam antibiotics or doxycycline (Class III) are administered for 2–3 weeks. No further studies show any benefits of receiving antibiotic treatment for more than 3 weeks. Therefore, a risk-benefit analysis finds that there is no scientific basis for deviating from the previous recommendation of administering antibiotics for 2–3 weeks to patients with late manifestations.

Moreover, doxycycline has also been shown to be equally effective in reducing CSF pleocytosis in 26 patients with Lyme encephalitis and/or myelitis compared to 115 patients with radicular manifestation (Bannwarth’s syndrome) (Class Ib) [231]. Based on the data, doxycycline is assumed to be effective regardless of the severity of the symptoms – as the authors conclude – however this has not been proven.

Polyneuritis associated with ACA improves clinically – albeit slowly – after antibiotic treatment, while electrophysiological abnormalities do not change significantly after a mean follow-up period of 18.5 months (range 11–50 months) [232]. The authors regard this finding to be a partial recovery rather than an indication of a persistent infection.

### 5.4 Cerebral vasculitis resulting from Lyme borreliosis

There are no controlled studies on the treatment of – very rare – cerebral vasculitis resulting from Lyme borreliosis. Case reports, case series and narrative reviews have reported that early antibiotic treatment with ceftriaxone and/or doxycycline has very good results [74], [75], [231], [233], [234], [235], [236] (Class IV). Several authors administer steroids in addition to antibiotics [235], [237], [238], [239] (Class IV). Despite antibiotic and steroid administration, clinical stabilization was achieved in 2 casuistics only after a subsequent immunosuppressive cyclophosphamide treatment (Class IV); two cases involving the basilar artery were lethal [240], [241]. In summary, in the case of cerebral vasculitis due to Lyme borreliosis, the earliest possible antibiotic treatment is in the foreground; whether the addition of steroids and/or prophylactic platelet function inhibition with ASA 100mg, in analogy to the recommendations in autoimmune mediated cerebral vasculitis, results in an advantage is unclear (DGN S1 guideline on cerebral vasculitis, AWMF Register No. 030-085 [242]).

### 5.5 Treating Lyme neuroborreliosis in children

According to a systematic review [3], the current scientific data on the antibiotic treatment of Lyme neuroborreliosis in children is very limited and existing studies are of a low quality. Two RCTs and four NRSs (one prospective and three retrospective cohort studies) were identified as being analysable studies. These are all older studies, some of which are several decades old, and do not meet current standards for clinical trials. The treatment duration was 14 days in the RCTs and 10–30 days in the NRSs. Different treatment durations were not compared. Only one prospective cohort study required a positive CSF finding in the sense of a “probable” case of Lyme neuroborreliosis as an inclusion criterion; all other studies based their inclusion criteria on a “possible” case of Lyme neuroborreliosis, which does not require the detection of inflammatory changes in CSF for a diagnosis and thus carries the risk of recruiting false positive cases. Penicillin G was investigated most frequently (5 studies), followed by ceftriaxone (4 studies) and doxycycline (2 studies). No studies examined the antibiotics hydroxychloroquine, azithromycin, minocycline or carbapenem antibiotics. Three studies compared several beta-lactam antibiotics, one study compared beta-lactam antibiotics with doxycycline, and two studies investigated various treatment regimens. Apart from one cohort study, all studies showed a critical overall risk for bias. This pertained to the recruitment process, randomisation, blinding, confounding of baseline data and data evaluation and/or data report, so that the results can only be used to a very limited extent for treatment recommendations. When comparing beta-lactam antibiotics with doxycycline, none of the studies showed a statistically significant difference, although the wide confidence intervals place limitations on this statement. The same applies to the comparison of penicillin G with ceftriaxone. In one study no side effects of the penicillin G group were reported, however there was a moderate allergic skin reaction (n=1), increase in liver enzymes (n=2) and asymptomatic gallbladder concrements (n=6) in the ceftriaxone group. The gallbladder concrements were detected by an ultrasound screening conducted on the ceftriaxone group, but not on the penicillin comparison group. The side effects reported in the other studies could not be assigned to the respective interventions and could therefore not be evaluated. Differentiated recommendations for clinical use cannot be derived from the limited study data. However, the prognosis for Lyme neuroborreliosis in children appears to be favourable across all studies. Poor results or an inadequate treatment response were rarely reported regardless of the antibiotic used.

#### Recommendations for treating children and adults

- Antibiotic treatment should be carried out in the case of Lyme neuroborreliosis with inflammatory cerebrospinal fluid syndrome (probable or confirmed Lyme neuroborreliosis) (Section 3.4). ↑↑ (strong consensus 13/13)
- In the case of a “possible” Lyme neuroborreliosis (CSF not available or inconspicuous) (Section 3.4), antibiotic treatment may be considered after a thorough differential diagnosis and if there is no evidence of another disease. ↔ (13/13)
- Antibiotic treatment should take place over a period of 14 days (early Lyme neuroborreliosis) or 14–21 days (late Lyme neuroborreliosis). ↑↑
- Reference is made to the S2k Guideline “Cutaneous Lyme Borreliosis” (AWMF Register No. 013-044) [1] for the treatment of polyneuropathy associated with acrodermatitis chronica atrophicans (ACA). (consensus 12/13)
- If a distally symmetrical polyneuropathy is suspected as a manifestation of Lyme neuroborreliosis without accompanying ACA (rare in Europe), the same procedure recommended for “possible” Lyme neuroborreliosis can be used. ↔ (consensus 10/13)
- Cerebral vasculitis resulting from Lyme borreliosis should be treated with antibiotics in accordance with the recommendations for “late Lyme neuroborreliosis”. ↑↑ (strong consensus 12/12)
- Analogous to the recommendations for cerebral vasculitis of another aetiology (DGN S1 guideline on cerebral vasculitis, AWMF Register No. 030-085 [242]), the additional administration of steroids and/or 100 mg/d of ASS can be considered for cerebral vasculitis resulting from Lyme borreliosis. ↔ (consensus 10/12)
- In addition to antibiotic treatment, symptoms should also be treated (physiotherapy, physical therapy, occupational therapy, speech therapy, neuropsychological training, psychosocial measures, the administration of analgesics, rehabilitative measures). ↑ (strong consensus 13/13)

#### Recommendation for choosing antibiotics for children and adults

- Antibiotic treatment of early and late Lyme neuroborreliosis should be performed with one of the following substances: doxycycline, ceftriaxone, cefotaxime, penicillin G. The choice of antibiotic should be based on the individual patient (allergies, other tolerability, age, pregnancy, mode and frequency of application, etc.). ↑↑ (consensus 9/13) (Table 5 (Tab. 5))

#### Recommendations for monitoring the treatment of children and adults

- The success of the treatment should be assessed on the basis of the clinical symptoms. ↑↑ (strong consensus 12/12)
- If clinical deterioration occurs during or after treatment, the differential diagnoses should be reviewed on an interdisciplinary basis. ↑ (strong consensus 10/10)
- If a patient continues to have impairing symptoms 6 months after treatment, the diagnostic testing of CSF should be repeated; if there are doubts about whether the symptoms are improving, an earlier follow-up CSF analysis can be considered; if the pleocytosis persists, a new course of antibiotic treatment should be carried out after review of the differential diagnosis. ↑ (11/13 consensus)
- The following parameters should not be used to monitor treatment ↓↓ (strong consensus 13/13):
  - Borrelia-specific antibody concentrations (and/or titre) in serum
  - Borrelia-specific CSF/serum antibody index
  - Oligoclonal bands in CSF
  - Total protein in CSF
  - Band pattern in Lyme immunoblot

## Notes

### Guideline information

This is the English version of the German DGN S3 Guideline “Neuroborreliose”, AWMF Register No. 030-071 [243].

- Development stage: S3
- Lead coordinator: Prof. Dr. Sebastian Rauer
- Deputy coordinator: PD Dr. Stephan Kastenbauer
- Published by the Guideline Commission of the German Neurological Society

#### Version

- Completely revised: 21 March 2018
- German version online at https://www.awmf.org since: 12 April 2018 [243]
- Valid until: 12 April 2021
- Chapter: Inflammatory and pathogen-causing diseases

#### ICD 10 code

A69.2+, G01*, G63.0*

#### Internet

https://www.dgn.org/

https://www.awmf.org

### Support/Financing of the guideline

This guideline was created without the influence or financial support of sponsors.

The funds required to create and to translate this guideline were provided by the Society for Promotion of Quality Assurance in Medical Laboratories (INSTAND e.V.), Düsseldorf.

Travel expenses were provided by the respective expert medical societies.

See Attachment 4

### Competing interests

See Attachment 4

### Dissenting opinions

The DBG and the patient organisations BFBD, BZK and OnLyme-Aktion.org have issued dissenting opinions, which are published in an appendix (Attachment 1) to the guideline report (Attachment 2).

### Methods used to develop guideline

This guideline is based on an update of the S1 guideline “Neuroborreliosis” No. 030-071 issued by a panel of experts in 2012 [2]. The guideline was created in accordance with the methodological guidelines of the German Association of Scientific Medical Societies (AWMF) for the development and further development of diagnosis and treatment guidelines. It corresponds to an S3 guideline in accordance with AWMF’s three-stage concept. The composition of the guideline group was interdisciplinary (IDA).

Uniform formulations are used in order to standardise the recommendations of the guideline. The following gradations shall apply here:

- Strong recommendation: “shall” ↑↑
- Recommendation: “should” ↑
- Open recommendation: “may be considered” ↔
- Recommendation against an intervention: “should not” ↓
- Strong recommendation against an intervention: “shall not” ↓↓

### DGN guideline commission

#### Chair

Prof. Dr. med. Hans-Christoph Diener

Prof. Dr. med. Christian Gerloff (vice chairman)

#### Chief editor

Prof. Dr. med. Christian Weimar

#### Members (in alphabetical order)

Prof. Dr. med. Peter Berlit (Vertreter der Chefärzte), Prof. Dr. med. Claudio L. A. Bassetti (Vertreter der SNG), Dr. med. Uwe Meier (Vertreter der Niedergelassenen), Prof. Dr. med. Jörg R. Weber (Vertreter der ÖGN), Prof. Dr. med. Claudia Sommer (Vertreterin für Schmerzen und PNP), Prof. Dr. med. Dr. h.c. Günther Deuschl, PD Dr. med. Karla Eggert, Prof. Dr. med. Christian Elger, Prof. Dr. med. Gereon R. Fink, Prof. Dr. med. Peter U. Heuschmann, Prof. Dr. med. Andreas Hufschmidt, Prof. Dr. med. Thomas Lempert, Prof. Dr. med. Dr. h.c. Wolfgang H. Oertel, Prof. Dr. med. Hans Walter Pfister, Prof. Dr. med. Heinz Reichmann, PD Dr. Christiane Schneider-Gold, Prof. Dr. med. Bernhard J. Steinhoff, Prof. Dr. med. Lars Timmermann, Prof. Dr. med. Claus W. Wallesch, Prof. Dr. med. Christian Weimar, Prof. Dr. med. Michael Weller, Prof. Dr. med. Wolfgang Wick

## Supplementary Material

Dissenting opinions

Guideline report

Appendix

Conflict of interest

## Figures and Tables

**Table 1:**
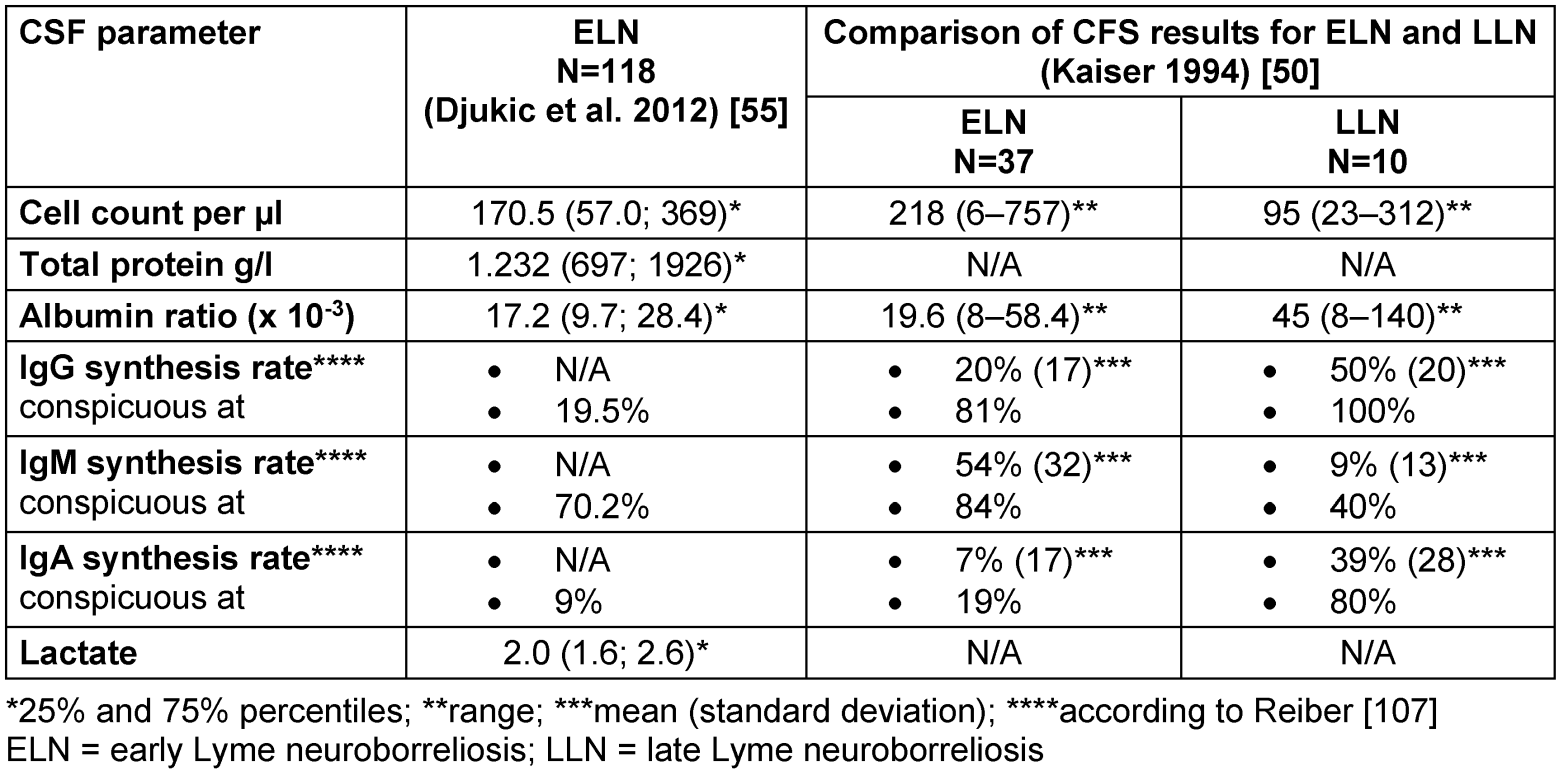
Cerebrospinal fluid results for early and late manifestations of Lyme neuroborreliosis prior to antibiotic treatment

**Table 2:**
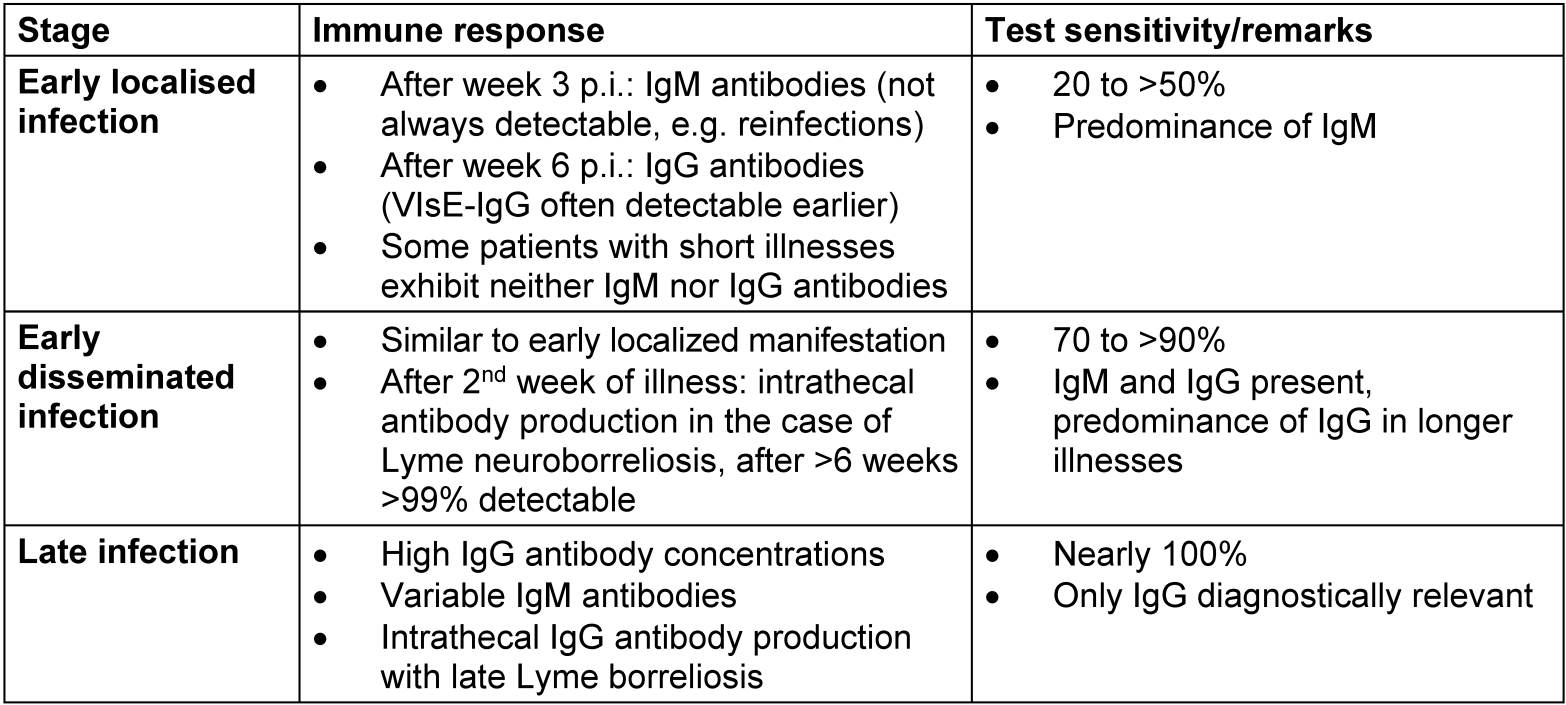
Antibody detection and test sensitivity based on disease stage (modified according to [85])

**Table 3:**
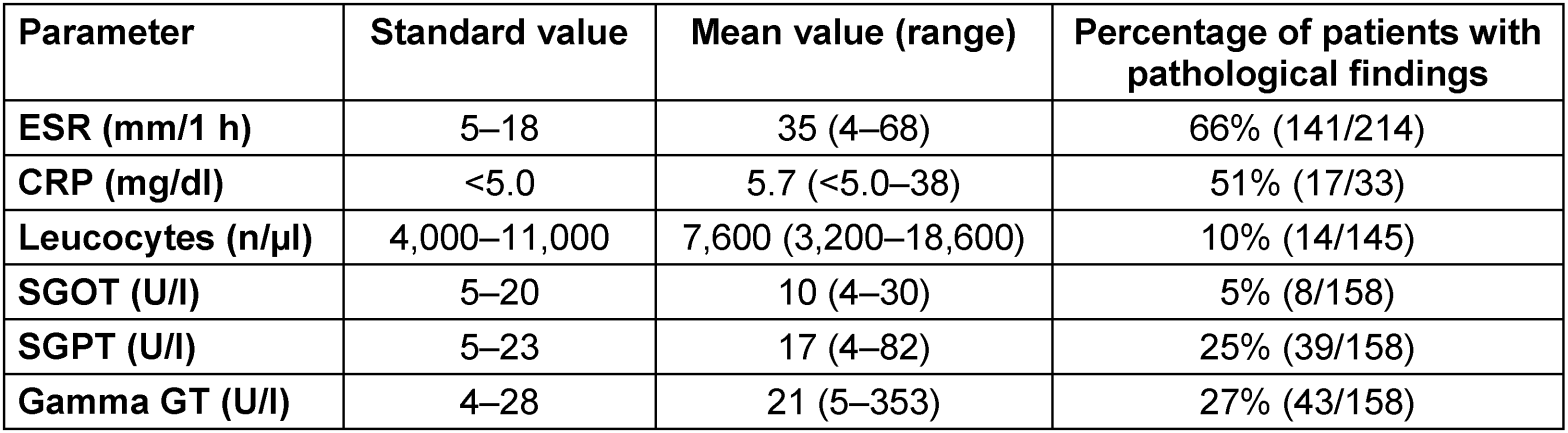
Routine lab parameters for patients with early or late manifestations of Lyme neuroborreliosis [47]

**Table 4:**
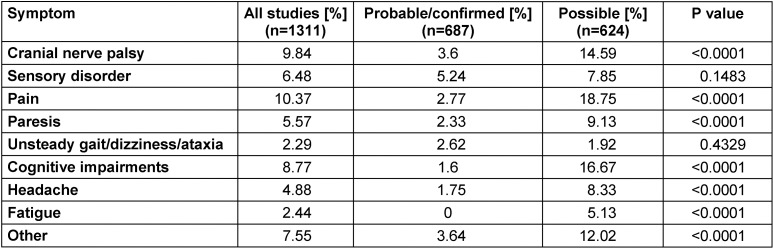
Systematic evaluation of the frequency of persistent symptoms following the treatment of Lyme neuroborreliosis in relation to diagnostic certainty (probable/confirmed vs. possible) (modified according to [5])

**Table 5:**
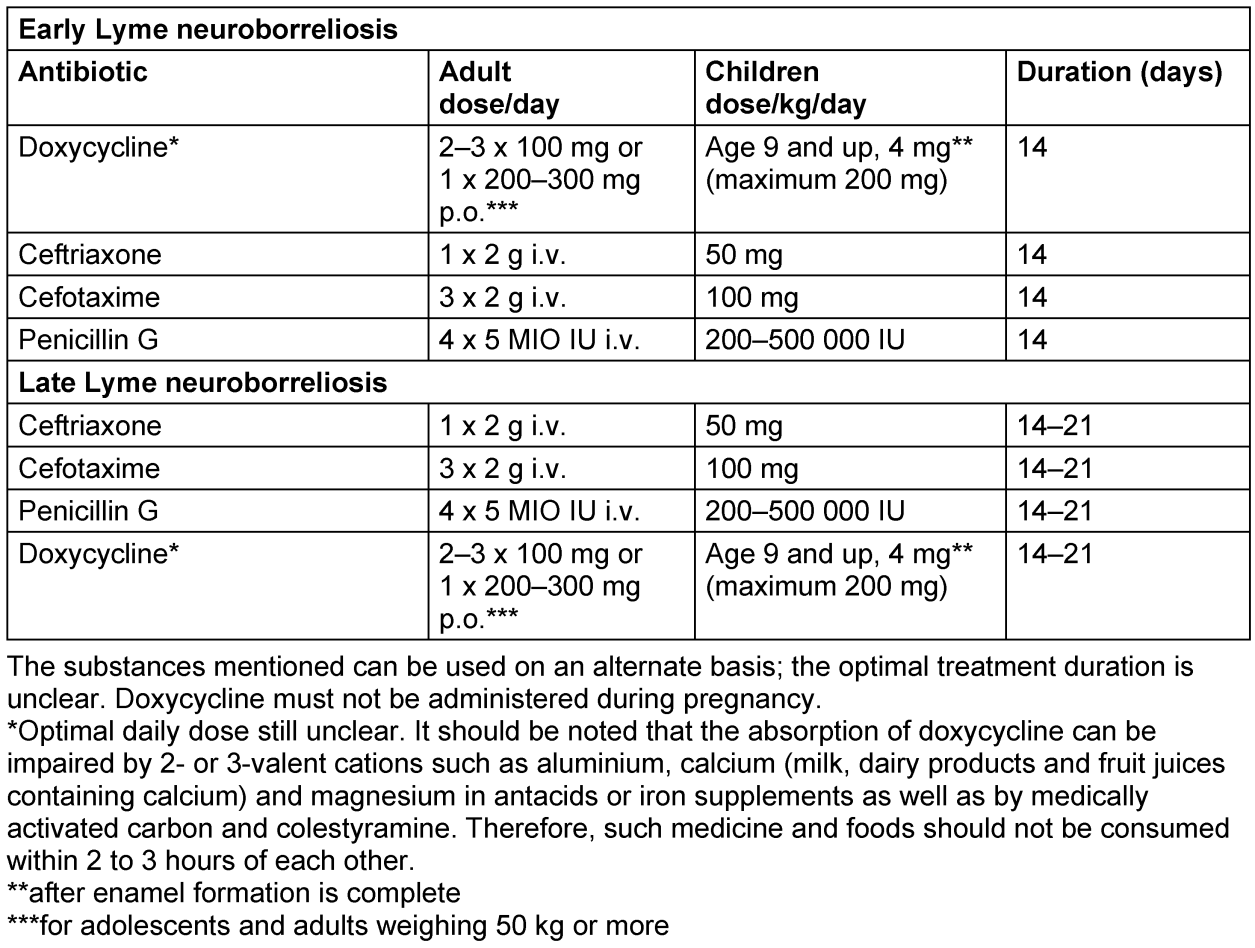
Overview of antibiotic treatment

## References

[1] Deutsche Gesellschaft für Dermatologie (2016). S2k Leitlinie Kutane Lyme Borreliose. AWMF-Register No. 013-044.

[2] Deutsche Gesellschaft für Neurologie (2012). S1 Leitlinie Neuroborreliose. AWMF Register No. 030-071.

[3] Dersch R, Hottenrott T, Schmidt S, Sommer H, Huppertz HI, Rauer S, Meerpohl JJ (2016). Efficacy and safety of pharmacological treatments for Lyme neuroborreliosis in children: a systematic review. BMC Neurol.

[4] Dersch R, Freitag MH, Schmidt S, Sommer H, Rauer S, Meerpohl JJ (2015). Efficacy and safety of pharmacological treatments for acute Lyme neuroborreliosis – a systematic review. Eur J Neurol.

[5] Dersch R, Sommer H, Rauer S, Meerpohl JJ (2016). Prevalence and spectrum of residual symptoms in Lyme neuroborreliosis after pharmacological treatment: a systematic review. J Neurol.

[6] Fingerle V, Schulte-Spechtel UC, Ruzic-Sabljic E, Leonhard S, Hofmann H, Weber K, Pfister K, Strle F, Wilske B (2008). Epidemiological aspects and molecular characterization of Borrelia burgdorferi s.l. from southern Germany with special respect to the new species Borrelia spielmanii sp. nov. Int J Med Microbiol.

[7] van Dam AP, Kuiper H, Vos K, Widjojokusumo A, de Jongh BM, Spanjaard L, Ramselaar AC, Kramer MD, Dankert J (1993). Different genospecies of Borrelia burgdorferi are associated with distinct clinical manifestations of Lyme borreliosis. Clin Infect Dis.

[8] Margos G, Vollmer SA, Cornet M, Garnier M, Fingerle V, Wilske B, Bormane A, Vitorino L, Collares-Pereira M, Drancourt M, Kurtenbach K (2009). A new Borrelia species defined by multilocus sequence analysis of housekeeping genes. Appl Environ Microbiol.

[9] Wilking H, Stark K (2014). Trends in surveillance data of human Lyme borreliosis from six federal states in eastern Germany, 2009–2012. Ticks Tick Borne Dis.

[10] Müller I, Freitag MH, Poggensee G, Scharnetzky E, Straube E, Schoerner C, Hlobil H, Hagedorn HJ, Stanek G, Schubert-Unkmeir A, Norris DE, Gensichen J, Hunfeld KP (2012). Evaluating frequency, diagnostic quality, and cost of Lyme borreliosis testing in Germany: a retrospective model analysis. Clin Dev Immunol.

[11] Huppertz HI, Böhme M, Standaert SM, Karch H, Plotkin SA (1999). Incidence of Lyme borreliosis in the Würzburg region of Germany. Eur J Clin Microbiol Infect Dis.

[12] Berglund J, Eitrem R, Ornstein K, Lindberg A, Ringér A, Elmrud H, Carlsson M, Runehagen A, Svanborg C, Norrby R (1995). An epidemiologic study of Lyme disease in southern Sweden. N Engl J Med.

[13] Kaiser R, Kern A, Kampa D, Neumann-Haefelin D (1997). Prevalence of antibodies to Borrelia burgdorferi and tick-borne encephalitis virus in an endemic region in southern Germany. Zentralbl Bakteriol.

[14] Stanek G, Flamm H, Groh V, Hirschl A, Kristoferitsch W, Neumann R, Schmutzhard E, Wewalka G (1987). Epidemiology of borrelia infections in Austria. Zentralbl Bakteriol Mikrobiol Hyg A.

[15] Wilking H, Fingerle V, Klier C, Thamm M, Stark K (2015). Antibodies against Borrelia burgdorferi sensu lato among Adults, Germany, 2008–2011. Emerging Infect Dis.

[16] Fahrer H, Sauvain MJ, Zhioua E, Van Hoecke C, Gern LE (1998). Longterm survey (7 years) in a population at risk for Lyme borreliosis: what happens to the seropositive individuals?. Eur J Epidemiol.

[17] Dehnert M, Fingerle V, Klier C, Talaska T, Schlaud M, Krause G, Wilking H, Poggensee G (2012). Seropositivity of Lyme borreliosis and associated risk factors: a population-based study in children and adolescents in Germany (KiGGS). PLoS ONE.

[18] Wilske B, Steinhuber R, Bergmeister H, Fingerle V, Schierz G, Preac-Mursic V, Vanek E, Lorbeer B (1987). Lyme-Borreliose in Süddeutschland. Epidemiologische Daten zum Auftreten von Erkrankungsfällen sowie zur Durchseuchung von Zecken (Ixodes ricinus) mit Borrelia burgdorferi. Dtsch Med Wochenschr.

[19] Jouda F, Perret JL, Gern L (2004). Density of questing Ixodes ricinus nymphs and adults infected by Borrelia burgdorferi sensu lato in Switzerland: spatio-temporal pattern at a regional scale. Vector Borne Zoonotic Dis.

[20] Crippa M, Rais O, Gern L (2002). Investigations on the mode and dynamics of transmission and infectivity of Borrelia burgdorferi sensu stricto and Borrelia afzelii in Ixodes ricinus ticks. Vector Borne Zoonotic Dis.

[21] Munderloh UG, Kurtti TJ (2005). The ABCs of Lyme disease spirochaetes in ticks. Lancet.

[22] Heininger U, Zimmermann T, Schoerner C, Brade V, Stehr K (1993). Zeckenstich und Lyme-Borreliose. Eine epidemiologische Untersuchung im Raum Erlangen. Monatsschr Kinderheilkd.

[23] Maiwald M, Oehme R, March O, Petney TN, Kimmig P, Naser K, Zappe HA, Hassler D, von Knebel Doeberitz M (1998). Transmission risk of Borrelia burgdorferi sensu lato from Ixodes ricinus ticks to humans in southwest Germany. Epidemiol Infect.

[24] Paul H, Gerth HJ, Ackermann R (1987). Infectiousness for humans of Ixodes ricinus containing Borrelia burgdorferi. Zentralbl Bakteriol Mikrobiol Hyg A.

[25] Nahimana I, Gern L, Blanc DS, Praz G, Francioli P, Péter O (2004). Risk of Borrelia burgdorferi infection in western Switzerland following a tick bite. Eur J Clin Microbiol Infect Dis.

[26] Gern L (2009). Life cycle of Borrelia burgdorferi sensu lato and transmission to humans. Curr Probl Dermatol.

[27] Wormser GP, Dattwyler RJ, Shapiro ED, Halperin JJ, Steere AC, Klempner MS, Krause PJ, Bakken JS, Strle F, Stanek G, Bockenstedt L, Fish D, Dumler JS, Nadelman RB (2006). The clinical assessment, treatment, and prevention of lyme disease, human granulocytic anaplasmosis, and babesiosis: clinical practice guidelines by the Infectious Diseases Society of America. Clin Infect Dis.

[28] Leenders AC (2001). Single-dose doxycycline for the prevention of Lyme disease. N Engl J Med.

[29] Nadelman RB, Nowakowski J, Fish D, Falco RC, Freeman K, McKenna D, Welch P, Marcus R, Agüero-Rosenfeld ME, Dennis DT, Wormser GP, Tick Bite Study Group (2001). Prophylaxis with single-dose doxycycline for the prevention of Lyme disease after an Ixodes scapularis tick bite. N Engl J Med.

[30] Wilske B (2005). Epidemiology and diagnosis of Lyme borreliosis. Ann Med.

[31] Knauer J, Krupka I, Fueldner C, Lehmann J, Straubinger RK (2011). Evaluation of the preventive capacities of a topically applied azithromycin formulation against Lyme borreliosis in a murine model. J Antimicrob Chemother.

[32] Piesman J, Hojgaard A, Ullmann AJ, Dolan MC (2014). Efficacy of an experimental azithromycin cream for prophylaxis of tick-transmitted lyme disease spirochete infection in a murine model. Antimicrob Agents Chemother.

[33] Schwameis M, Kündig T, Huber G, von Bidder L, Meinel L, Weisser R, Aberer E, Härter G, Weinke T, Jelinek T, Fätkenheuer G, Wollina U, Burchard GD, Aschoff R, Nischik R, Sattler G, Popp G, Lotte W, Wiechert D, Eder G, Maus O, Staubach-Renz P, Gräfe A, Geigenberger V, Naudts I, Sebastian M, Reider N, Weber R, Heckmann M, Reisinger EC, Klein G, Wantzen J, Jilma B (2017). Topical azithromycin for the prevention of Lyme borreliosis: a randomised, placebo-controlled, phase 3 efficacy trial. Lancet Infect Dis.

[34] Wallich R, Kramer MD, Simon MM (1996). The recombinant outer surface protein A (lipOspA) of Borrelia burgdorferi: a Lyme disease vaccine. Infection.

[35] Steere AC, Sikand VK, Meurice F, Parenti DL, Fikrig E, Schoen RT, Nowakowski J, Schmid CH, Laukamp S, Buscarino C, Krause DS (1998). Vaccination against Lyme disease with recombinant Borrelia burgdorferi outer-surface lipoprotein A with adjuvant. Lyme Disease Vaccine Study Group. N Engl J Med.

[36] Kalish RS, Wood JA, Golde W, Bernard R, Davis LE, Grimson RC, Coyle PK, Luft BJ (2003). Human T lymphocyte response to Borrelia burgdorferi infection: no correlation between human leukocyte function antigen type 1 peptide response and clinical status. J Infect Dis.

[37] Abbott A (2006). Lyme disease: uphill struggle. Nature.

[38] Nigrovic LE, Thompson KM (2007). The Lyme vaccine: a cautionary tale. Epidemiol Infect.

[39] Barrett PN, Portsmouth D (2013). A novel multivalent OspA vaccine against Lyme borreliosis shows promise in Phase I/II studies. Expert Rev Vaccines.

[40] Stanek G, Fingerle V, Hunfeld KP, Jaulhac B, Kaiser R, Krause A, Kristoferitsch W, O’Connell S, Ornstein K, Strle F, Gray J (2011). Lyme borreliosis: clinical case definitions for diagnosis and management in Europe. Clin Microbiol Infect.

[41] Hansen K, Lebech AM (1992). The clinical and epidemiological profile of Lyme neuroborreliosis in Denmark 1985–1990. A prospective study of 187 patients with Borrelia burgdorferi specific intrathecal antibody production. Brain.

[42] Kaiser R (2004). Verlauf der akuten und chronischen Neuroborreliose nach Behandlung mit Ceftriaxon. Nervenarzt.

[43] Koedel U, Fingerle V, Pfister HW (2015). Lyme neuroborreliosis – epidemiology, diagnosis and management. Nat Rev Neurol.

[44] Stanek G, Wormser GP, Gray J, Strle F (2012). Lyme borreliosis. Lancet.

[45] Stanek G, Strle F (2009). Lyme borreliosis: a European perspective on diagnosis and clinical management. Curr Opin Infect Dis.

[46] Steere AC (1989). Lyme disease. N Engl J Med.

[47] Oschmann P, Dorndorf W, Hornig C, Schäfer C, Wellensiek HJ, Pflughaupt KW (1998). Stages and syndromes of neuroborreliosis. J Neurol.

[48] Henningsson AJ, Malmvall BE, Ernerudh J, Matussek A, Forsberg P (2010). Neuroborreliosis – an epidemiological, clinical and healthcare cost study from an endemic area in the south-east of Sweden. Clin Microbiol Infect.

[49] Kaiser R, Fingerle V (2009). Neuroborreliose. Nervenarzt.

[50] Kaiser R (1994). Variable CSF findings in early and late Lyme neuroborreliosis: a follow-up study in 47 patients. J Neurol.

[51] Christen HJ (1996). Lyme neuroborreliosis in children. Ann Med.

[52] Pfister HW, Wilske B, Weber K (1994). Lyme borreliosis: basic science and clinical aspects. Lancet.

[53] Reik L, Steere AC, Bartenhagen NH, Shope RE, Malawista SE (1979). Neurologic abnormalities of Lyme disease. Medicine (Baltimore).

[54] Rupprecht TA, Koedel U, Fingerle V, Pfister HW (2008). The pathogenesis of lyme neuroborreliosis: from infection to inflammation. Mol Med.

[55] Djukic M, Schmidt-Samoa C, Lange P, Spreer A, Neubieser K, Eiffert H, Nau R, Schmidt H (2012). Cerebrospinal fluid findings in adults with acute Lyme neuroborreliosis. J Neurol.

[56] Dotevall L, Hagberg L (1999). Successful oral doxycycline treatment of Lyme disease-associated facial palsy and meningitis. Clin Infect Dis.

[57] Drack FD, Weissert M (2013). Outcome of peripheral facial palsy in children – a catamnestic study. Eur J Paediatr Neurol.

[58] Kowalski TJ, Berth WL, Mathiason MA, Agger WA (2011). Oral antibiotic treatment and long-term outcomes of Lyme facial nerve palsy. Infection.

[59] Steere AC, Pachner AR, Malawista SE (1983). Neurologic abnormalities of Lyme disease: successful treatment with high-dose intravenous penicillin. Ann Intern Med.

[60] Kristoferitsch W, Sluga E, Graf M, Partsch H, Neumann R, Stanek G, Budka H (1988). Neuropathy associated with acrodermatitis chronica atrophicans. Clinical and morphological features. Ann N Y Acad Sci.

[61] Kindstrand E, Nilsson BY, Hovmark A, Pirskanen R, Asbrink E (1997). Peripheral neuropathy in acrodermatitis chronica atrophicans – a late Borrelia manifestation. Acta Neurol Scand.

[62] Halperin J, Luft BJ, Volkman DJ, Dattwyler RJ (1990). Lyme neuroborreliosis. Peripheral nervous system manifestations. Brain.

[63] Logigian EL, Steere AC (1992). Clinical and electrophysiologic findings in chronic neuropathy of Lyme disease. Neurology.

[64] Farhad K, Traub R, Ruzhansky KM, Brannagan TH (2016). Causes of neuropathy in patients referred as “idiopathic neuropathy”. Muscle Nerve.

[65] Mygland A, Skarpaas T, Ljøstad U (2006). Chronic polyneuropathy and Lyme disease. Eur J Neurol.

[66] Hassler D, Zöller L, Haude M, Hufnagel HD, Sonntag HG (1992). Lyme-Borreliose in einem europäischen Endemiegebiet. Antikörperprävalenz und klinisches Spektrum. Dtsch Med Wochenschr.

[67] Fingerle V, Goodman JL, Johnson RC, Kurtti TJ, Munderloh UG, Wilske B (1997). Human granulocytic ehrlichiosis in southern Germany: increased seroprevalence in high-risk groups. J Clin Microbiol.

[68] Hess A, Buchmann J, Zettl UK, Henschel S, Schlaefke D, Grau G, Benecke R (1999). Borrelia burgdorferi central nervous system infection presenting as an organic schizophrenialike disorder. Biol Psychiatry.

[69] Pasareanu AR, Mygland Å, Kristensen Ø (2012). A woman in her 50s with manic psychosis. Tidsskr Nor Laegeforen.

[70] Roelcke U, Barnett W, Wilder-Smith E, Sigmund D, Hacke W (1992). Untreated neuroborreliosis: Bannwarth’s syndrome evolving into acute schizophrenia-like psychosis. A case report. J Neurol.

[71] Markeljević J, Sarac H, Rados M (2011). Tremor, seizures and psychosis as presenting symptoms in a patient with chronic lyme neuroborreliosis (LNB). Coll Antropol.

[72] Császár T, Patakfalvi A (1994). Differenciáldiagnosztikai problémák Lyme-kórban (Borrelia fertözés okozta akut exogen reakciós típusú psychosis). Orv Hetil.

[73] Riedel M, Straube A, Schwarz MJ, Wilske B, Müller N (1998). Lyme disease presenting as Tourette’s syndrome. Lancet.

[74] Wittwer B, Pelletier S, Ducrocq X, Maillard L, Mione G, Richard S (2015). Cerebrovascular Events in Lyme Neuroborreliosis. J Stroke Cerebrovasc Dis.

[75] Zajkowska J, Garkowski A, Moniuszko A, Czupryna P, Ptaszyńska-Sarosiek I, Tarasów E, Ustymowicz A, Łebkowski W, Pancewicz S (2015). Vasculitis and stroke due to Lyme neuroborreliosis – a review. Infect Dis (Lond).

[76] Schmutzhard E, Willeit J, Gerstenbrand F (1986). Meningopolyneuritis Bannwarth with focal nodular myositis. A new aspect in Lyme borreliosis. Klin Wochenschr.

[77] Reimers CD, de Koning J, Neubert U, Preac-Mursic V, Koster JG, Müller-Felber W, Pongratz DE, Duray PH (1993). Borrelia burgdorferi myositis: report of eight patients. J Neurol.

[78] Huppertz HI, Sticht-Groh V (1989). Meningitis due to Borrelia burgdorferi in the initial stage of Lyme disease. Eur J Pediatr.

[79] Huppertz HI, Bartmann P, Heininger U, Fingerle V, Kinet M, Klein R, Korenke GC, Nentwich HJ, Committee for Infectious Diseases and Vaccinations of the German Academy for Pediatrics and Adolescent Health (2012). Rational diagnostic strategies for Lyme borreliosis in children and adolescents: recommendations by the Committee for Infectious Diseases and Vaccinations of the German Academy for Pediatrics and Adolescent Health. Eur J Pediatr.

[80] Huisman TA, Wohlrab G, Nadal D, Boltshauser E, Martin E (1999). Unusual presentations of neuroborreliosis (Lyme disease) in childhood. J Comput Assist Tomogr.

[81] Wilke M, Eiffert H, Christen HJ, Hanefeld F (2000). Primarily chronic and cerebrovascular course of Lyme neuroborreliosis: case reports and literature review. Arch Dis Child.

[82] Vukelic D, Bozinovic D, Morovic M, Tesovic G, Ruzic Sabljic E, Barisic N, Knezovic I (2000). Opsoclonus-myoclonus syndrome in a child with neuroborreliosis. J Infect.

[83] Ylitalo V, Hagberg BA (1994). Progressive ataxia in Swedish children: a re-evaluation study. Acta Neurol Scand.

[84] Halperin JJ, Logigian EL, Finkel MF, Pearl RA (1996). Practice parameters for the diagnosis of patients with nervous system Lyme borreliosis (Lyme disease). Quality Standards Subcommittee of the American Academy of Neurology. Neurology.

[85] Fingerle V, Eiffert H, Gessner A, Göbel U, Hofmann H, Hunfeld KP, Krause A, Pfister HW, Reischl U, Sing A, Stanek G, Wilske B, Zöller L (2017). Lyme Borreliose.

[86] Reiber H, Ressel CB, Spreer A (2013). Diagnosis of Neuroborreliosis – Improved knowledge base for qualified antibody analysis and cerebrospinal fluid data pattern related interpretations. Neurol Psychiatry Brain Res.

[87] Wilske B, Fingerle V, Schulte-Spechtel U (2007). Microbiological and serological diagnosis of Lyme borreliosis. FEMS Immunol Med Microbiol.

[88] Eiffert H, Hanefeld F, Thomssen R, Christen HJ (1996). Reinfection in Lyme borreliosis. Infection.

[89] Steere AC (1993). Seronegative Lyme disease. JAMA.

[90] Hammers-Berggren S, Lebech AM, Karlsson M, Svenungsson B, Hansen K, Stiernstedt G (1994). Serological follow-up after treatment of patients with erythema migrans and neuroborreliosis. J Clin Microbiol.

[91] Hilton E, Tramontano A, DeVoti J, Sood SK (1997). Temporal study of immunoglobin M seroreactivity to Borrelia burgdorferi in patients treated for Lyme borreliosis. J Clin Microbiol.

[92] Kalish RA, McHugh G, Granquist J, Shea B, Ruthazer R, Steere AC (2001). Persistence of immunoglobulin M or immunoglobulin G antibody responses to Borrelia burgdorferi 10–20 years after active Lyme disease. Clin Infect Dis.

[93] Wilske B, Zöller L, Brade V, Eiffert H, Göbel U, Stanek G, Pfister HW (2000). Mikrobiologische Qualitätsstandards Lyme-Borreliose.

[94] Wilske B, Johnson BJ, Schriefer ME, Murray PR, Baron EJ, Jorgensen HJ, Landry ML, Pfaller MA (2007). Borrelia. Manual of Clinical Microbiology.

[95] Goettner G, Schulte-Spechtel U, Hillermann R, Liegl G, Wilske B, Fingerle V (2005). Improvement of Lyme borreliosis serodiagnosis by a newly developed recombinant immunoglobulin G (IgG) and IgM line immunoblot assay and addition of VlsE and DbpA homologues. J Clin Microbiol.

[96] Wilske B, Fingerle V, Herzer P, Hofmann A, Lehnert G, Peters H, Pfister HW, Preac-Mursic V, Soutschek E, Weber K (1993). Recombinant immunoblot in the serodiagnosis of Lyme borreliosis. Comparison with indirect immunofluorescence and enzyme-linked immunosorbent assay. Med Microbiol Immunol.

[97] Liang FT, Aberer E, Cinco M, Gern L, Hu CM, Lobet YN, Ruscio M, Voet PE, Weynants VE, Philipp MT (2000). Antigenic conservation of an immunodominant invariable region of the VlsE lipoprotein among European pathogenic genospecies of Borrelia burgdorferi SL. J Infect Dis.

[98] Hauser U, Lehnert G, Wilske B (1999). Validity of interpretation criteria for standardized Western blots (immunoblots) for serodiagnosis of Lyme borreliosis based on sera collected throughout Europe. J Clin Microbiol.

[99] Hauser U, Lehnert G, Lobentanzer R, Wilske B (1997). Interpretation criteria for standardized Western blots for three European species of Borrelia burgdorferi sensu lato. J Clin Microbiol.

[100] Zöller L, Burkard S, Schäfer H (1991). Validity of western immunoblot band patterns in the serodiagnosis of Lyme borreliosis. J Clin Microbiol.

[101] Tumani H, Nölker G, Reiber H (1995). Relevance of cerebrospinal fluid variables for early diagnosis of neuroborreliosis. Neurology.

[102] Blanc F, Jaulhac B, Fleury M, de Seze J, de Martino SJ, Remy V, Blaison G, Hansmann Y, Christmann D, Tranchant C (2007). Relevance of the antibody index to diagnose Lyme neuroborreliosis among seropositive patients. Neurology.

[103] Ljøstad U, Skarpaas T, Mygland A (2007). Clinical usefulness of intrathecal antibody testing in acute Lyme neuroborreliosis. Eur J Neurol.

[104] Kaiser R, Rauer S (1998). Analysis of the intrathecal immune response in neuroborreliosis to a sonicate antigen and three recombinant antigens of Borrelia burgdorferi sensu stricto. Eur J Clin Microbiol Infect Dis.

[105] Reiber H, Peter JB (2001). Cerebrospinal fluid analysis: disease-related data patterns and evaluation programs. J Neurol Sci.

[106] Reiber H, Lange P (1991). Quantification of virus-specific antibodies in cerebrospinal fluid and serum: sensitive and specific detection of antibody synthesis in brain. Clin Chem.

[107] Reiber H, Felgenhauer K (1987). Protein transfer at the blood cerebrospinal fluid barrier and the quantitation of the humoral immune response within the central nervous system. Clin Chim Acta.

[108] Kaiser R, Lücking CH (1993). Intrathecal synthesis of specific antibodies in neuroborreliosis. Comparison of different ELISA techniques and calculation methods. J Neurol Sci.

[109] Rauer S, Kaiser R, Kölmel HW, Pfister HW, Schmutzhard E, Sturzenegger M, Wilske B, Diener HC, Weimar C (2012). Neuroborreliose. Leitlinien für Diagnostik und Therapie in der Neurologie.

[110] Hansen K, Lebech AM (1991). Lyme neuroborreliosis: a new sensitive diagnostic assay for intrathecal synthesis of Borrelia burgdorferi-specific immunoglobulin G, A, and M. Ann Neurol.

[111] Christen HJ, Hanefeld F, Eiffert H, Thomssen R (1993). Epidemiology and clinical manifestations of Lyme borreliosis in childhood. A prospective multicentre study with special regard to neuroborreliosis. Acta Paediatr Suppl.

[112] Baig S, Olsson T, Hansen K, Link H (1991). Anti-Borrelia burgdorferi antibody response over the course of Lyme neuroborreliosis. Infect Immun.

[113] Krüger H, Reuss K, Pulz M, Rohrbach E, Pflughaupt KW, Martin R, Mertens HG (1989). Meningoradiculitis and encephalomyelitis due to Borrelia burgdorferi: a follow-up study of 72 patients over 27 years. J Neurol.

[114] Hammers-Berggren S, Hansen K, Lebech AM, Karlsson M (1993). Borrelia burgdorferi-specific intrathecal antibody production in neuroborreliosis: a follow-up study. Neurology.

[115] Rupprecht TA, Pfister HW, Angele B, Kastenbauer S, Wilske B, Koedel U (2005). The chemokine CXCL13 (BLC): a putative diagnostic marker for neuroborreliosis. Neurology.

[116] Schmidt C, Plate A, Angele B, Pfister HW, Wick M, Koedel U, Rupprecht TA (2011). A prospective study on the role of CXCL13 in Lyme neuroborreliosis. Neurology.

[117] Ljøstad U, Mygland A (2008). CSF B-lymphocyte chemoattractant (CXCL13) in the early diagnosis of acute Lyme neuroborreliosis. J Neurol.

[118] Rupprecht TA, Lechner C, Tumani H, Fingerle V (2014). CXCL13 als Biomarker der akuten Neuroborreliose: Überprüfung des prädiktiven Wertes in der klinischen Routine. Nervenarzt.

[119] Rupprecht TA, Plate A, Adam M, Wick M, Kastenbauer S, Schmidt C, Klein M, Pfister HW, Koedel U (2009). The chemokine CXCL13 is a key regulator of B cell recruitment to the cerebrospinal fluid in acute Lyme neuroborreliosis. J Neuroinflammation.

[120] Hytönen J, Kortela E, Waris M, Puustinen J, Salo J, Oksi J (2014). CXCL13 and neopterin concentrations in cerebrospinal fluid of patients with Lyme neuroborreliosis and other diseases that cause neuroinflammation. J Neuroinflammation.

[121] Dersch R, Hottenrott T, Senel M, Lehmensiek V, Tumani H, Rauer S, Stich O (2015). The chemokine CXCL13 is elevated in the cerebrospinal fluid of patients with neurosyphilis. Fluids Barriers CNS.

[122] Rubenstein JL, Wong VS, Kadoch C, Gao HX, Barajas R, Chen L, Josephson SA, Scott B, Douglas V, Maiti M, Kaplan LD, Treseler PA, Cha S, Hwang JH, Cinque P, Cyster JG, Lowell C (2013). CXCL13 plus interleukin 10 is highly specific for the diagnosis of CNS lymphoma. Blood.

[123] Keller TL, Halperin JJ, Whitman M (1992). PCR detection of Borrelia burgdorferi DNA in cerebrospinal fluid of Lyme neuroborreliosis patients. Neurology.

[124] Lebech AM, Hansen K, Brandrup F, Clemmensen O, Halkier-Sørensen L (2000). Diagnostic value of PCR for detection of Borrelia burgdorferi DNA in clinical specimens from patients with erythema migrans and Lyme neuroborreliosis. Mol Diagn.

[125] Kaiser R (1998). Neuroborreliosis. J Neurol.

[126] Dattwyler RJ, Volkman DJ, Luft BJ, Halperin JJ, Thomas J, Golightly MG (1988). Seronegative Lyme disease. Dissociation of specific T- and B-lymphocyte responses to Borrelia burgdorferi. N Engl J Med.

[127] Valentine-Thon E, Müller K, Guzzi G, Kreisel S, Ohnsorge P, Sandkamp M (2006). LTT-MELISA is clinically relevant for detecting and monitoring metal sensitivity. Neuro Endocrinol Lett.

[128] von Baehr V, Doebis C, Volk HD, von Baehr R (2012). The lymphocyte transformation test for borrelia detects active lyme borreliosis and verifies effective antibiotic treatment. Open Neurol J.

[129] Dessau RB, Fingerle V, Gray J, Hunfeld KP, Jaulhac B, Kahl O, Kristoferitsch W, Stanek G, Strle F (2014). The lymphocyte transformation test for the diagnosis of Lyme borreliosis has currently not been shown to be clinically useful. Clin Microbiol Infect.

[130] Nordberg M, Forsberg P, Nyman D, Skogman BH, Nyberg C, Ernerudh J, Eliasson I, Ekerfelt C (2012). Can ELISPOT be applied to a clinical setting as a diagnostic utility for neuroborreliosis?. Cells.

[131] Marques A, Telford SR, Turk SP, Chung E, Williams C, Dardick K, Krause PJ, Brandeburg C, Crowder CD, Carolan HE, Eshoo MW, Shaw PA, Hu LT (2014). Xenodiagnosis to detect Borrelia burgdorferi infection: a first-in-human study. Clin Infect Dis.

[132] Bockenstedt LK, Radolf JD (2014). Xenodiagnosis for posttreatment Lyme disease syndrome: resolving the conundrum or adding to it?. Clin Infect Dis.

[133] Hartmann F, Müller-Marienburg H (2003). Indirekter Neurotoxinnachweis durch den „Visual Contrast Sensitivity“-Test bei Patienten mit einer chronischen Borreliose. Med Welt.

[134] Lantos PM, Auwaerter PG, Wormser GP (2014). A systematic review of Borrelia burgdorferi morphologic variants does not support a role in chronic Lyme disease. Clin Infect Dis.

[135] Stricker RB, Winger EE (2001). Decreased CD57 lymphocyte subset in patients with chronic Lyme disease. Immunol Lett.

[136] Smit PW, Kurkela S, Kuusi M, Vapalahti O (2015). Evaluation of two commercially available rapid diagnostic tests for Lyme borreliosis. Eur J Clin Microbiol Infect Dis.

[137] Halperin JJ (2015). Nervous system Lyme disease. Infect Dis Clin North Am.

[138] Halperin JJ (2014). Nervous system Lyme disease. Handb Clin Neurol.

[139] Borchers AT, Keen CL, Huntley AC, Gershwin ME (2015). Lyme disease: a rigorous review of diagnostic criteria and treatment. J Autoimmun.

[140] Oliveira CR, Shapiro ED (2015). Update on persistent symptoms associated with Lyme disease. Curr Opin Pediatr.

[141] Cameron DJ (2010). Proof that chronic lyme disease exists. Interdiscip Perspect Infect Dis.

[142] Feder HM, Johnson BJ, O’Connell S, Shapiro ED, Steere AC, Wormser GP, Ad Hoc International Lyme Disease Group, Agger WA, Artsob H, Auwaerter P, Dumler JS, Bakken JS, Bockenstedt LK, Green J, Dattwyler RJ, Munoz J, Nadelman RB, Schwartz I, Draper T, McSweegan E, Halperin JJ, Klempner MS, Krause PJ, Mead P, Morshed M, Porwancher R, Radolf JD, Smith RP Jr, Sood S, Weinstein A, Wong SJ, Zemel L (2007). A critical appraisal of “chronic Lyme disease”. N Engl J Med.

[143] Cameron DJ, Johnson LB, Maloney EL (2014). Evidence assessments and guideline recommendations in Lyme disease: the clinical management of known tick bites, erythema migrans rashes and persistent disease. Expert Rev Anti Infect Ther.

[144] Perronne C (2015). Critical review of studies trying to evaluate the treatment of chronic Lyme disease. Presse Med.

[145] Baker PJ (2012). The pain of “chronic Lyme disease”: moving the discourse in a different direction. FASEB J.

[146] Halperin JJ (2015). Chronic Lyme disease: misconceptions and challenges for patient management. Infect Drug Resist.

[147] Johnson L, Wilcox S, Mankoff J, Stricker RB (2014). Severity of chronic Lyme disease compared to other chronic conditions: a quality of life survey. PeerJ.

[148] Vrethem M, Hellblom L, Widlund M, Ahl M, Danielsson O, Ernerudh J, Forsberg P (2002). Chronic symptoms are common in patients with neuroborreliosis – a questionnaire follow-up study. Acta Neurol Scand.

[149] Lantos PM, Wormser GP (2014). Chronic coinfections in patients diagnosed with chronic lyme disease: a systematic review. Am J Med.

[150] Reid MC, Schoen RT, Evans J, Rosenberg JC, Horwitz RI (1998). The consequences of overdiagnosis and overtreatment of Lyme disease: an observational study. Ann Intern Med.

[151] Steere AC, Taylor E, McHugh GL, Logigian EL (1993). The overdiagnosis of Lyme disease. JAMA.

[152] Sigal LH (1990). Summary of the first 100 patients seen at a Lyme disease referral center. Am J Med.

[153] Hassett AL, Radvanski DC, Buyske S, Savage SV, Sigal LH (2009). Psychiatric comorbidity and other psychological factors in patients with “chronic Lyme disease”. Am J Med.

[154] Ljøstad U, Mygland Å (2012). The phenomenon of 'chronic Lyme'; an observational study. Eur J Neurol.

[155] Djukic M, Schmidt-Samoa C, Nau R, von Steinbüchel N, Eiffert H, Schmidt H (2011). The diagnostic spectrum in patients with suspected chronic Lyme neuroborreliosis – the experience from one year of a university hospital’s Lyme neuroborreliosis outpatients clinic. Eur J Neurol.

[156] Coumou J, Herkes EA, Brouwer MC, van de Beek D, Tas SW, Casteelen G, van Vugt M, Starink MV, de Vries HJ, de Wever B, Spanjaard L, Hovius JW (2015). Ticking the right boxes: classification of patients suspected of Lyme borreliosis at an academic referral center in the Netherlands. Clin Microbiol Infect.

[157] Borgermans L, Goderis G, Vandevoorde J, Devroey D (2014). Relevance of chronic lyme disease to family medicine as a complex multidimensional chronic disease construct: a systematic review. Int J Family Med.

[158] Lightfoot RW, Luft BJ, Rahn DW, Steere AC, Sigal LH, Zoschke DC, Gardner P, Britton MC, Kaufman RL (1993). Empiric parenteral antibiotic treatment of patients with fibromyalgia and fatigue and a positive serologic result for Lyme disease. A cost-effectiveness analysis. Ann Intern Med.

[159] Tugwell P, Dennis DT, Weinstein A, Wells G, Shea B, Nichol G, Hayward R, Lightfoot R, Baker P, Steere AC (1997). Laboratory evaluation in the diagnosis of Lyme disease. Ann Intern Med.

[160] Benedetti F (2014). Placebo effects: from the neurobiological paradigm to translational implications. Neuron.

[161] Nieman GF, Zerler BR (2001). A role for the anti-inflammatory properties of tetracyclines in the prevention of acute lung injury. Curr Med Chem.

[162] Tikka T, Usenius T, Tenhunen M, Keinänen R, Koistinaho J (2001). Tetracycline derivatives and ceftriaxone, a cephalosporin antibiotic, protect neurons against apoptosis induced by ionizing radiation. J Neurochem.

[163] Leite LM, Carvalho AG, Ferreira PL, Pessoa IX, Gonçalves DO, Lopes Ade A, Góes JG, Alves VC, Leal LK, Brito GA, Viana GS (2011). Anti-inflammatory properties of doxycycline and minocycline in experimental models: an in vivo and in vitro comparative study. Inflammopharmacology.

[164] Berglund J, Stjernberg L, Ornstein K, Tykesson-Joelsson K, Walter H (2002). 5-y Follow-up study of patients with neuroborreliosis. Scand J Infect Dis.

[165] Ljøstad U, Mygland A (2010). Remaining complaints 1 year after treatment for acute Lyme neuroborreliosis; frequency, pattern and risk factors. Eur J Neurol.

[166] Eikeland R, Mygland Å, Herlofson K, Ljøstad U (2013). Risk factors for a non-favorable outcome after treated European neuroborreliosis. Acta Neurol Scand.

[167] Treib J, Fernandez A, Haass A, Grauer MT, Holzer G, Woessner R (1998). Clinical and serologic follow-up in patients with neuroborreliosis. Neurology.

[168] Aucott JN, Crowder LA, Kortte KB (2013). Development of a foundation for a case definition of post-treatment Lyme disease syndrome. Int J Infect Dis.

[169] Luo N, Johnson JA, Shaw JW, Feeny D, Coons SJ (2005). Self-reported health status of the general adult U.S. population as assessed by the EQ-5D and Health Utilities Index. Med Care.

[170] Wessely S (2001). Chronic fatigue: symptom and syndrome. Ann Intern Med.

[171] Skogman BH, Croner S, Nordwall M, Eknefelt M, Ernerudh J, Forsberg P (2008). Lyme neuroborreliosis in children: a prospective study of clinical features, prognosis, and outcome. Pediatr Infect Dis J.

[172] Skogman BH, Glimåker K, Nordwall M, Vrethem M, Ödkvist L, Forsberg P (2012). Long-term clinical outcome after Lyme neuroborreliosis in childhood. Pediatrics.

[173] Wang TJ, Sangha O, Phillips CB, Wright EA, Lew RA, Fossel AH, Fossel K, Shadick NA, Liang MH, Sundel RP (1998). Outcomes of children treated for Lyme disease. J Rheumatol.

[174] Dersch R, Sarnes AA, Maul M, Hottenrott T, Baumgartner A, Rauer S, Stich O (2015). Quality of life, fatigue, depression and cognitive impairment in Lyme neuroborreliosis. J Neurol.

[175] Cerar D, Cerar T, Ruzić-Sabljić E, Wormser GP, Strle F (2010). Subjective symptoms after treatment of early Lyme disease. Am J Med.

[176] Kalish RA, Kaplan RF, Taylor E, Jones-Woodward L, Workman K, Steere AC (2001). Evaluation of study patients with Lyme disease, 10-20-year follow-up. J Infect Dis.

[177] Seltzer EG, Gerber MA, Cartter ML, Freudigman K, Shapiro ED (2000). Long-term outcomes of persons with Lyme disease. JAMA.

[178] Eikeland R, Mygland A, Herlofson K, Ljøstad U (2011). European neuroborreliosis: quality of life 30 months after treatment. Acta Neurol Scand.

[179] Vázquez M, Sparrow SS, Shapiro ED (2003). Long-term neuropsychologic and health outcomes of children with facial nerve palsy attributable to Lyme disease. Pediatrics.

[180] Shadick NA, Phillips CB, Logigian EL, Steere AC, Kaplan RF, Berardi VP, Duray PH, Larson MG, Wright EA, Ginsburg KS, Katz JN, Liang MH (1994). The long-term clinical outcomes of Lyme disease. A population-based retrospective cohort study. Ann Intern Med.

[181] Shadick NA, Phillips CB, Sangha O, Logigian EL, Kaplan RF, Wright EA, Fossel AH, Fossel K, Berardi V, Lew RA, Liang MH (1999). Musculoskeletal and neurologic outcomes in patients with previously treated Lyme disease. Ann Intern Med.

[182] Aucott JN, Rebman AW, Crowder LA, Kortte KB (2013). Post-treatment Lyme disease syndrome symptomatology and the impact on life functioning: is there something here?. Qual Life Res.

[183] Cairns V, Godwin J (2005). Post-Lyme borreliosis syndrome: a meta-analysis of reported symptoms. Int J Epidemiol.

[184] Shapiro ED, Dattwyler R, Nadelman RB, Wormser GP (2005). Response to meta-analysis of Lyme borreliosis symptoms. Int J Epidemiol.

[185] Chandra AM, Keilp JG, Fallon BA (2013). Correlates of perceived health-related quality of life in post-treatment Lyme encephalopathy. Psychosomatics.

[186] Benke T, Gasse T, Hittmair-Delazer M, Schmutzhard E (1995). Lyme encephalopathy: long-term neuropsychological deficits years after acute neuroborreliosis. Acta Neurol Scand.

[187] Eikeland R, Ljøstad U, Mygland A, Herlofson K, Løhaugen GC (2012). European neuroborreliosis: neuropsychological findings 30 months post-treatment. Eur J Neurol.

[188] Bujak DI, Weinstein A, Dornbush RL (1996). Clinical and neurocognitive features of the post Lyme syndrome. J Rheumatol.

[189] Krupp LB, Masur D, Schwartz J, Coyle PK, Langenbach LJ, Fernquist SK, Jandorf L, Halperin JJ (1991). Cognitive functioning in late Lyme borreliosis. Arch Neurol.

[190] Kaplan RF, Meadows ME, Vincent LC, Logigian EL, Steere AC (1992). Memory impairment and depression in patients with Lyme encephalopathy: comparison with fibromyalgia and nonpsychotically depressed patients. Neurology.

[191] Gaudino EA, Coyle PK, Krupp LB (1997). Post-Lyme syndrome and chronic fatigue syndrome. Neuropsychiatric similarities and differences. Arch Neurol.

[192] Keilp JG, Corbera K, Slavov I, Taylor MJ, Sackeim HA, Fallon BA (2006). WAIS-III and WMS-III performance in chronic Lyme disease. J Int Neuropsychol Soc.

[193] Fallon BA, Keilp JG, Corbera KM, Petkova E, Britton CB, Dwyer E, Slavov I, Cheng J, Dobkin J, Nelson DR, Sackeim HA (2008). A randomized, placebo-controlled trial of repeated IV antibiotic therapy for Lyme encephalopathy. Neurology.

[194] Elkins LE, Pollina DA, Scheffer SR, Krupp LB (1999). Psychological states and neuropsychological performances in chronic Lyme disease. Appl Neuropsychol.

[195] Kaplan RF, Trevino RP, Johnson GM, Levy L, Dornbush R, Hu LT, Evans J, Weinstein A, Schmid CH, Klempner MS (2003). Cognitive function in post-treatment Lyme disease: do additional antibiotics help?. Neurology.

[196] Klempner MS, Hu LT, Evans J, Schmid CH, Johnson GM, Trevino RP, Norton D, Levy L, Wall D, McCall J, Kosinski M, Weinstein A (2001). Two controlled trials of antibiotic treatment in patients with persistent symptoms and a history of Lyme disease. N Engl J Med.

[197] Krupp LB, Hyman LG, Grimson R, Coyle PK, Melville P, Ahnn S, Dattwyler R, Chandler B (2003). Study and treatment of post Lyme disease (STOP-LD): a randomized double masked clinical trial. Neurology.

[198] Klempner MS, Baker PJ, Shapiro ED, Marques A, Dattwyler RJ, Halperin JJ, Wormser GP (2013). Treatment trials for post-Lyme disease symptoms revisited. Am J Med.

[199] Schulz KF, Grimes DA (2002). Sample size slippages in randomised trials: exclusions and the lost and wayward. Lancet.

[200] Lantos PM (2011). Chronic Lyme disease: the controversies and the science. Expert Rev Anti Infect Ther.

[201] Halperin JJ (2014). Lyme disease: neurology, neurobiology, and behavior. Clin Infect Dis.

[202] Nowakowski J, Nadelman RB, Sell R, McKenna D, Cavaliere LF, Holmgren D, Gaidici A, Wormser GP (2003). Long-term follow-up of patients with culture-confirmed Lyme disease. Am J Med.

[203] Asch ES, Bujak DI, Weiss M, Peterson MG, Weinstein A (1994). Lyme disease: an infectious and postinfectious syndrome. J Rheumatol.

[204] Klempner MS (2002). Controlled trials of antibiotic treatment in patients with post-treatment chronic Lyme disease. Vector Borne Zoonotic Dis.

[205] Wormser GP, Nadelman RB, Dattwyler RJ, Dennis DT, Shapiro ED, Steere AC, Rush TJ, Rahn DW, Coyle PK, Persing DH, Fish D, Luft BJ (2000). Practice guidelines for the treatment of Lyme disease. The Infectious Diseases Society of America. Clin Infect Dis.

[206] Deutsche Gesellschaft für Allgemeinmedizin und Familienmedizin (2017). S3 Leitlinie Müdigkeit. AWMF Register No. 053-002.

[207] Deutsche Schmerzgesellschaft (2012). S3 Leitlinie Fibromyalgiesyndrom. AWMF Register No. 041-004.

[208] Deutsche Gesellschaft für Allgemeinmedizin und Familienmedizin (2013). S1 Leitlinie Chronischer Schmerz. AWMF Register No. 053-036.

[209] Deutsche Gesellschaft für Psychiatrie und Psychotherapie, Psychosomatik und Nervenheilkunde, Bundesärztekammer, Kassenärztliche Bundesvereinigung, Arbeitsgemeinschaft der Wissenschaftlichen Medizinischen Fachgesellschaften (2015). S3 Leitlinie/Nationale Versorgungsleitlinie Unipolare Depression. AWMF Register No. nvl-005.

[210] Deutsche Gesellschaft für Neurologie (2019). S2e Leitlinie Diagnostik und Therapie von Gedächtnisstörungen. AWMF Register No. 030-124.

[211] Deutsches Kollegium für Psychosomatische Medizin, Deutsche Gesellschaft für Psychosomatische Medizin und Ärztliche Psychotherapie (2018). S3 Leitlinie Funktionelle Körperbeschwerden. AWMF Register No. 051-001.

[212] Halperin JJ, Pass HL, Anand AK, Luft BJ, Volkman DJ, Dattwyler RJ (1988). Nervous system abnormalities in Lyme disease. Ann N Y Acad Sci.

[213] Halperin JJ, Krupp LB, Golightly MG, Volkman DJ (1990). Lyme borreliosis-associated encephalopathy. Neurology.

[214] Logigian EL, Kaplan RF, Steere AC (1990). Chronic neurologic manifestations of Lyme disease. N Engl J Med.

[215] Young GB (2013). Encephalopathy of infection and systemic inflammation. J Clin Neurophysiol.

[216] Halperin JJ (2016). Nervous system Lyme disease, chronic Lyme disease, and none of the above. Acta Neurol Belg.

[217] Dersch R, Freitag MH, Schmidt S, Sommer H, Rücker G, Rauer S, Meerpohl JJ (2014). Efficacy and safety of pharmacological treatments for neuroborreliosis – protocol for a systematic review. Syst Rev.

[218] Krüger H, Kohlhepp W, König S (1990). Follow-up of antibiotically treated and untreated neuroborreliosis. Acta Neurol Scand.

[219] Kristoferitsch W, Baumhackl U, Sluga E, Stanek G, Zeiler K (1987). High-dose penicillin therapy in meningopolyneuritis Garin-Bujadoux-Bannwarth. Clinical and cerebrospinal fluid data. Zentralbl Bakteriol Mikrobiol Hyg A.

[220] Hirsch E, Sellal F, Christmann D, Steinmetz G, Monteil H, Jesel M, Warter JM, Collard M (1987). Les méningo-radiculites après morsure de tique. Etude de 31 cas. Rev Neurol (Paris).

[221] Karlsson M, Hammers-Berggren S, Lindquist L, Stiernstedt G, Svenungsson B (1994). Comparison of intravenous penicillin G and oral doxycycline for treatment of Lyme neuroborreliosis. Neurology.

[222] Bensch J, Olcén P, Hagberg L (1987). Destructive chronic borrelia meningoencephalitis in a child untreated for 15 years. Scand J Infect Dis.

[223] Oksi J, Nikoskelainen J, Hiekkanen H, Lauhio A, Peltomaa M, Pitkäranta A, Nyman D, Granlund H, Carlsson SA, Seppälä I, Valtonen V, Viljanen M (2007). Duration of antibiotic treatment in disseminated Lyme borreliosis: a double-blind, randomized, placebo-controlled, multicenter clinical study. Eur J Clin Microbiol Infect Dis.

[224] Mygland A, Ljøstad U, Fingerle V, Rupprecht T, Schmutzhard E, Steiner I, European Federation of Neurological Societies (2010). EFNS guidelines on the diagnosis and management of European Lyme neuroborreliosis. Eur J Neurol.

[225] Halperin JJ, Shapiro ED, Logigian E, Belman AL, Dotevall L, Wormser GP, Krupp L, Gronseth G, Bever CT, Quality Standards Subcommittee of the American Academy of Neurology (2007). Practice parameter: treatment of nervous system Lyme disease (an evidence-based review): report of the Quality Standards Subcommittee of the American Academy of Neurology. Neurology.

[226] Ljøstad U, Skogvoll E, Eikeland R, Midgard R, Skarpaas T, Berg A, Mygland A (2008). Oral doxycycline versus intravenous ceftriaxone for European Lyme neuroborreliosis: a multicentre, non-inferiority, double-blind, randomised trial. Lancet Neurol.

[227] Pfister HW, Preac-Mursic V, Wilske B, Schielke E, Sörgel F, Einhäupl KM (1991). Randomized comparison of ceftriaxone and cefotaxime in Lyme neuroborreliosis. J Infect Dis.

[228] Pfister HW, Preac-Mursic V, Wilske B, Einhäupl KM (1989). Cefotaxime vs penicillin G for acute neurologic manifestations in Lyme borreliosis. A prospective randomized study. Arch Neurol.

[229] Hassler D, Zöller L, Haude M, Hufnagel HD, Heinrich F, Sonntag HG (1990). Cefotaxime versus penicillin in the late stage of Lyme disease – prospective, randomized therapeutic study. Infection.

[230] Ogrinc K, Lusa L, Lotrič-Furlan S, Bogovič P, Stupica D, Cerar T, Ružić-Sabljić E, Strle F (2016). Course and Outcome of Early European Lyme Neuroborreliosis (Bannwarth Syndrome): Clinical and Laboratory Findings. Clin Infect Dis.

[231] Bremell D, Dotevall L (2014). Oral doxycycline for Lyme neuroborreliosis with symptoms of encephalitis, myelitis, vasculitis or intracranial hypertension. Eur J Neurol.

[232] Kindstrand E, Nilsson BY, Hovmark A, Pirskanen R, Asbrink E (2002). Peripheral neuropathy in acrodermatitis chronica atrophicans – effect of treatment. Acta Neurol Scand.

[233] Topakian R, Stieglbauer K, Nussbaumer K, Aichner FT (2008). Cerebral vasculitis and stroke in Lyme neuroborreliosis. Two case reports and review of current knowledge. Cerebrovasc Dis.

[234] May EF, Jabbari B (1990). Stroke in neuroborreliosis. Stroke.

[235] Kohns M, Karenfort M, Schaper J, Laws HJ, Mayatepek E, Distelmaier F (2013). Transient ischaemic attack in a 5-year-old girl due to focal vasculitis in neuroborreliosis. Cerebrovasc Dis.

[236] Heinrich A, Khaw AV, Ahrens N, Kirsch M, Dressel A (2003). Cerebral vasculitis as the only manifestation of Borrelia burgdorferi infection in a 17-year-old patient with basal ganglia infarction. Eur Neurol.

[237] Back T, Grünig S, Winter Y, Bodechtel U, Guthke K, Khati D, von Kummer R (2013). Neuroborreliosis-associated cerebral vasculitis: long-term outcome and health-related quality of life. J Neurol.

[238] Schmiedel J, Gahn G, von Kummer R, Reichmann H (2004). Cerebral vasculitis with multiple infarcts caused by lyme disease. Cerebrovasc Dis.

[239] Lebas A, Toulgoat F, Saliou G, Husson B, Tardieu M (2012). Stroke due to lyme neuroborreliosis: changes in vessel wall contrast enhancement. J Neuroimaging.

[240] Schmitt AB, Küker W, Nacimiento W (1999). Neuroborreliose mit ausgeprägter zerebraler Vaskulitis und multiplen Hirninfarkten. Nervenarzt.

[241] Komdeur R, Zijlstra JG, van der Werf TS, Ligtenberg JJ, Tulleken JE (2001). Immunosuppressive treatment for vasculitis associated with Lyme borreliosis. Ann Rheum Dis.

[242] Deutsche Gesellschaft für Neurologie (2018). S1 Leitlinie Zerebrale Vaskulitis und zerebrale Beteiligung bei systemischen Vaskulitiden und rheumatischen Grunderkrankungen. AWMF Register No. 030-085.

[243] Deutsche Gesellschaft für Neurologie (2018). S3 Leitlinie Neuroborreliose. AWMF Register No. 030-071.

[244] Cameron D, Gaito A, Harris N, Bach G, Bellovin S, Bock K, Bock S, Burrascano J, Dickey C, Horowitz R, Phillips S, Meer-Scherrer L, Raxlen B, Sherr V, Smith H, Smith P, Stricker R, ILADS Working Group (2004). Evidence-based guidelines for the management of Lyme disease. Expert Rev Anti Infect Ther.

[245] Pfister HW, Einhäupl KM, Franz P, Garner C (1988). Corticosteroids for radicular pain in Bannwarth’s syndrome: A double-blind, randomized, placebo-controlled trial. Ann NY Acad Sci.

[246] Kohlhepp W, Oschmann P, Mertens HG (1989). Treatment of Lyme borreliosis. Randomized comparison of doxycycline and penicillin G. J Neurol.

[247] Borg R, Dotevall L, Hagberg L, Maraspin V, Lotric-Furlan S, Cimperman J, Strle F (2005). Intravenous ceftriaxone compared with oral doxycycline for the treatment of Lyme neuroborreliosis. Scand J Infect Dis.

