# Supplementary material for: Guidelines for diagnosis and treatment in neurology – Lyme neuroborreliosis
Source: Ger Med Sci. 2020 Feb 27;18:Doc03. doi: 10.3205/000279 (PMC7174852; doi:10.3205/000279)
Supplement: Dissenting opinions [file GMS-18-03-s-001.pdf]

Attachment 1: Dissenting opinions

BORRELIOSE UND FSME BUND DEUTSCHLAND
PATIENTENORGANISATION BUNDESVERBAND

Dissenting opinion on the Neuroborreliosis Guideline

As the largest borreliosis patient organisation in Germany, with over 2,500 members and supporting members, we were invited, but not involved, in the development of these guideline on neuroborreliosis. We had no opportunity to contribute our decades of patient experience. With respect to controversial topics, in particular the range of diagnostic testing methods as well as treatment dosages and duration, we were outvoted by the numerical dominance of mandate holders from professional associations. Studies – most of which were conducted outside of Europe and in the last century – were given more weight than current patient experience from Germany. Our request for two special votes to keep a window open for the responsible decision of the attending physician with regard to diagnosis and therapy was rejected for ostensibly formal reasons. As patients, we view ourselves misused in the role of an alibi just to attain the guideline classification S3.

Furthermore, we have doubts about the suitability of representatives from professional societies who receive substantial financial backing from pharmaceutical companies that economically profit from misdiagnoses and produce the aftermath of improper and inadequate therapies.

In the matter itself, we must insist that the attending physician not be restricted to serological blood tests, which, even according to the consensus of medical experts, are not sufficiently reliable in detecting the presence of (neuro) Lyme borreliosis, and which – as regards testing cerebrospinal fluid for intrathecal antibodies – represent a major intervention while providing insufficient certainty.

The present guideline suggests a high level of evidence despite the low number and quality of reliable studies. For example, about 24 per cent of patients with probable or certain neuroborreliosis are diagnosed with residual symptoms despite an absence of reliable definitions (Dersch et al.). Renowned physicians such as the Nobel laureate Luc Montagnier or Kim Lewis from Northeastern University in Boston, are tirelessly conducting research on Lyme borreliosis. This signifies that crucial questions pertaining to this insidious disease have by no means been answered and that an S3 guideline therefore does not seem justified from our point of view. Its content does not reflect any progress, neither for the patients and practitioners involved, nor for the expert assessments.

Borreliosis and FSME Association Germany www.borreliose-bund.de
Ute Fischer, Chair
9 October 2017

OnLyme-Aktion.org
Aktionsbündnis gegen zeckenübertragene Infektionen Deutschland e. V.

Ursula Dahlem
Am Haag 21
65812 Bad Soden

E-Mail: vorstand@onlyme-aktion.org
Tel: 06196 9504841
www.Onlyme-Aktion.org

5/9/2017

Statement from OnLyme-Aktion.org on the S3 guideline *Neuroborreliosis*

According to an internal survey, 97% of our members believe this guideline does not reflect an improvement over the "actual situation", something which would be necessary for those affected. **Based on this dissent report, OnLyme-Aktion.org does not support the final version of the guideline.**

Unfortunately, the recommendations in this guideline have only sparsely incorporated the experiences of the patients we represent. The reason for this may be that patients who seek help from our association and our forum have often been ill for a long time. The state of research is particularly thin for patients with late neuroborreliosis and/or with residual symptoms following neuroborreliosis. Other manifestations of Lyme borreliosis are not the subject of this guideline.

Based on our experience, many forms of later-stage Lyme borreliosis have neurological involvement in addition to chronic symptoms in the joints, skin, muscles and tendons. This is usually not only limited to the central nervous system but can also affect the peripheral or autonomic nervous system.

We are aware that the development methods necessary for the neuroborreliosis guideline to receive an S3 classification have been fulfilled. However, from our point of view, it suggests a higher level of evidence to the recipients of the guideline than the available studies should allow. The present guideline often contains strong recommendations even though it has been established that the number of robust studies on treating neuroborreliosis is low, the quality of these studies is low, and there is no information at all about some issues. It is not sufficiently clear that these are based less on the identified evidence than on subjective elements such as the clinical expertise of the consensus group. From our point of view, strong recommendations should only be used with caution in view of the paltry situation with regard to the studies.

Precisely because the other parts of the general S3 guideline on Lyme borreliosis have not yet been completed, we consider it all the more necessary to clarify in our public relations work that ruling out neuroborreliosis should not be equated with ruling out Lyme borreliosis. In many cases, the

neuroborreliosis guideline was referred to in the past in the medical assessments of manifestations of borreliosis that were not neuroborreliosis, even though the guideline was not applicable. We also feel it is necessary to clearly point out the significance of administering antibiotic treatment early on despite there often being uncertainty surrounding early-phase diagnostic testing. The good chances of curing the disease when treated early must not be abandoned in favour of a reliable diagnosis.

By referring to the guidelines of other professional societies it is insufficiently clear that an active case of neuroborreliosis cannot always be ruled out in later stages if there are persisting or newly occurring symptoms, especially if it is accompanied by comorbidities. Aspects of Lyme borreliosis are often not addressed in these other guidelines or there are no robust studies. The patients we represent usually do not benefit from the treatments recommended in these other guidelines, which aim at treating symptoms.

We therefore attach great importance to the significance and importance of probatory antibiotic treatment when specific IgG antibodies are present. This can be offered to patients following a differential diagnosis after indication that the diagnosis is unconfirmed (cf. 4.2.4). Although the recommendations on treatment monitoring (cf. 5.5) sometimes make reference to the necessity of a prolonged or repeated antibiotic therapy, it unfortunately does not include – with the exception of ACA – the necessity of longer treatments for other symptoms of Lyme borreliosis. Begging for treatment (not only in the context of neuroborreliosis!) is deeply humiliating and ethically unacceptable for people who have been ill for a long time.

Residual or persistent symptoms were found in about 24% of patients with a probable or confirmed cased of neuroborreliosis (Dersch et al., cf. 4.1). The cause of these symptoms cannot always be clearly identified. Our members therefore expect a greater receptiveness to extended treatment options of all kinds and the implementation of patient participation – also for patients with statutory health insurance – whose benefits should also be covered by the measures recommended in the guidelines. This is currently not always the case. To address patient concerns, we also expect increased research efforts into other reliable diagnostic options and treatment concepts for Lyme borreliosis.

The dramatic health, social, professional and existential consequences for the patients we represent must receive greater attention in the future.

In order to achieve these goals, we ask all participating associations and organisations to continue this constructive dialogue.

OnLyme-Aktion.org
Aktionsbündnis gegen zeckenübertragene
Infektionen Deutschland e.V.

c/o Krottorfer Str.122
57258 Freudenberg

E-Mail: kontakt@onlyme-aktion.org
http://www.onlyme-aktion.org
Fax: Fax +49(0)322 260 084 79

Sparkasse Leverkusen
Kto: 10009464
BLZ: 37551440
IBAN: DE 16 3755 1440 01000946 48
BIC: WELADEDLLEV
St-Nr: 230 /5722/4064 Finanzamt
Leverkusen
Vereinsregister 17392 Amtsgericht Köln

Bundesverband
Zecken-Krankheiten e. V.

Declaration of dissent to the S3 guideline on neuroborreliosis

For procedural reasons, the BZK was only able to participate in the processes at the very end, i.e. during the last 2 sessions of guideline discussions. The content of the guideline was largely developed without us!
Even though we agree to around 80% of what was stated in the neuroborreliosis guideline, we believe the following points could be greatly improved!

1) By submitting 6 international, evidence-based studies, we attempted unsuccessfully to incorporate into the guideline the Borna disease virus infection which is the most important differential diagnosis of neuroborreliosis. The reason may have been that BZK was late in becoming involved in the process. We therefore anticipate that this topic will be incorporated into the next version of the guideline.

2) Our objection to possible resistance by the Borrelia to conventional antibiotics in immunocompromised and immunodeficient people with many other primary diseases at the onset of borreliosis was not acknowledged, as one guideline point states that no resistance to conventional antibiotics has been identified in patients with **healthy immune systems**. We are unable to endorse this statement since most of the affected members of our association and self-help group are immunocompromised and immunodeficient patients.

3) The evidence-based studies on borreliosis used as a basis for this guideline were in part very outdated and therefore not in line with the current state of knowledge. A major portion of these studies came from the US and are based on a completely different borreliosis pathogen than the Borrelia species that are found in Germany (namely Borrelia. sensu stricto – actual Lyme borreliosis). This has a very low incidence rate here (experts estimate 10–15%). This appears to us to be a very important point in regard to treatment since we believe that other treatment parameters are necessary for this dangerous species and for the immunodeficient patients mentioned above!

   We would also like to remark that, for the most part, all participants tended to lump all 5 Borrelia species found in Germany together and that no necessary differentiation is made with respect to symptoms or treatment.
   Not to mention the lab testing methods can also be greatly improved.

Attachment 1 to: *Rauer S, Kastenbauer S, Hofmann H, Fingerle V, Huppertz HI, Hunfeld KP, Krause A, Ruf B, Dersch R, Consensus group. Guidelines for diagnosis and treatment in neurology – Lyme neuroborreliosis. GMS Ger Med Sci. 2020;18:Doc03. DOI: 10.3205/000279, URN: urn:nbn:de:0183-0002795*
Online available from: https://www.egms.de/en/journals/gms/2020-18/000279.shtml

Bundesverband
Zecken-Krankheiten e. V.

Bundesverband Zecken-Krankheiten e.V. (BZK)
Christel Schmedt, 1. Vorsitzende
www.bzk-online.de
17.10. 2017

Dissent Report

The German Borreliosis Society (DBG) has considerable objections to various aspects of the present guideline “Neuroborreliosis” issued by the German Society of Neurology (DGN). DGN rejected the inclusion of special votes and dissenting opinions for the corresponding section of the GL. The DBG’s objections are therefore presented in the following dissent report. DBG’s comments relate to various sections of the GL and are specified under the numbers corresponding to the guideline sections. This dissent report is divided into three sections: objections, explanations, literature. It is ordered in accordance with the numbering of the sections.

Objections

1.1.1

The term “neuroborreliosis” refers to all neurological manifestations of Lyme borreliosis. It is therefore not a disease in its own right.

1.1.3

Neurological symptoms occur only in 15% of Lyme borreliosis cases. About 85% of LB patients are neurologically inconspicuous.

The publication by Huppertz et al. 1999 included a preselected cohort and only focused on the incidence of Lyme neuroborreliosis and not on the frequency of individual disease manifestations.

EM appears in a maximum of 70% of Lyme borreliosis cases, i.e. EM is absent in 30% of LB patients.

CSF testing is not indicated for Lyme borreliosis without neurological manifestations.

Calculating the frequency of different disease manifestations based on the literature (1–11) results in the following data:

**Disease manifestations**	**Frequency**
Erythema migrans	50%
Acrodermatitis chronica atrophicans (ACA)	10%
Flu-like symptoms	80%
Fatigue	80%
Joint pain	70%
Muscle pain	93%
Headaches	60%
Brain dysfunction	50%
Mental illness	N/A
Sleep disorders	70%
Paraesthesia	40%
Arthritis	30%
Sore throat	25%
Sweating	20%
Cardiac symptoms	15%
Recurring rashes	15%
Neuroborreliosis	15%
Acute neuroborreliosis (stage II)	3.5%
Peripheral facial palsy	N/A
Meningoradiculitis (Bannwarth's syndrome)	N/A
Polyneuropathy	N/A
Eye disease	10%

N/A = not available

(1–18)

2

No literature is available on the frequency of late Lyme neuroborreliosis. Therefore, the claim that late manifestations are rare cannot be substantiated.

2.2

Studies on the frequency of polyneuropathy without ACA in Europe are not available.

3

The diagnostic algorithm for late neuroborreliosis (Figure 2 in the GL text) must be qualified since for encephalitis and myelitis there are no data on the frequency of pleocytosis and intrathecal antibodies. When CSF is inconspicuous, late Lyme neuroborreliosis cannot be ruled out. Moreover, the algorithm is limited to encephalitis, myelitis and meningitis; other manifestations of late Lyme neuroborreliosis (polyneuropathy, neuroradiculitis, cranial neuropathy) are not taken into account.

3.3.1

Seronegativity, i.e. the absence of antibodies, occurs in 30% of patients with late Lyme borreliosis. A frequency of nearly 100% for IgG antibodies in a "late infection", as indicated in Table 2 of the GL, has not been scientifically proven.

3.10

The diagnostic criteria for neuroborreliosis apply to acute Lyme neuroborreliosis, i.e. the early stage; there is insufficient data on the late stage.

3.11

Pleocytosis only occurs in connection with meningitis in LLN. Pleocytosis is always detectable in acute Lyme neuroborreliosis. For other forms of Lyme neuroborreliosis, especially for late stage (encephalitis, myelitis, radiculitis, cranial neuropathy, plexopathy, neuritis, neuritis multiplex), there is insufficient literature to assess the frequency of pleocytosis. Pleocytosis is therefore not requisite for this manifestation.

The algorithm for late neuroborreliosis presented in the GL (Figure 2) pertains exclusively to encephalomyelitis, encephalitis, myelitis and chronic meningitis.

The lymphocyte transformation test (LTT, von Baehr et al. 2012) plays an integral role in diagnosing Lyme borreliosis, especially in cases with typical clinical symptoms and seronegativity.

4.1

PTLDS is a hypothesis. These were symptoms that occurred with confirmed Lyme borreliosis and did not disappear after antibiotic treatment. In this context it is assumed that adequate standard treatment based on opinions (recommendations) of different professional associations and not on evidence-based studies, guarantees the elimination of Lyme borreliosis. In practised medicine and in forensic contexts, a connection between Lyme borreliosis and PTLDS is often negated without stating any reasons; there is no scientific literature regarding this issue. – It is not possible to distinguish between late Lyme borreliosis and so-called PTLDS. Late Lyme borreliosis is clearly defined in the scientific literature as a medical condition, so it is substantiated, whereas PTLDS is a hypothesis. A distinction between fact and hypothesis is not compatible with the laws of logic.

4.2.4.

Chronic Lyme borreliosis and chronic Lyme neuroborreliosis are conceptually identical to late stage LB and LLB respectively. LLB with persisting infection after antibiotic treatment is described multiple times in the literature and often proven by pathogen detection. The medical condition is based on a persisting infection and requires adequate antibiotic treatment.

4.4

Encephalopathy occurs in at least 60% of cases of late Lyme borreliosis and late Lyme neuroborreliosis. It leads to considerable cognitive and affective disorders with corresponding effects on social functions.

5.3

In the GL it is aptly stated that there are no evidence-based studies on the efficacy of antibiotic treatment for late Lyme neuroborreliosis. The data on late neuroborreliosis, as listed in Table 5 “Overview of antibiotic treatment”, are therefore not scientifically proven, nor does the GL state the basis for the treatment recommendation.

Explanations

1.1.1

The term “neuroborreliosis” refers to all neurological manifestations of Lyme borreliosis. Such a remark is particularly important since numerous paragraphs and sections of the GL text deviate from actual neurological issues.

1.1.3

It is important to note that neurological manifestations only occur in 10–15% of cases of Lyme borreliosis, i.e. that the vast majority of LB patients do not exhibit any neurological symptoms.

The DBG is of the opinion that the publication of Huppertz et al. 1999 is an investigation of a preselected cohort. The study exclusively focused on determining incidence and is unable to comment on the frequency of manifestations. This is because the frequency of neuroborreliosis is only indicated at 3%, arthritis at 5% and the absence of erythema migrans at 10%.

The study by Huppertz et al. 1999 does not allow any statement to be made on the frequency of EM. The statement that EM is the only symptom in 92% of cases is contradicted by other literature (1–18).

2

The work cited in the GL by Stanek and Strle 2009 and by Stanek et al. 2012 states nothing about the frequency of late Lyme neuroborreliosis. The publication by Stanek et al. 2012, is a general review of the diagnosis, treatment and prevention of Lyme neuroborreliosis. This publication also does not contain any information on the frequency of Lyme neuroborreliosis. The paper by Steere 1998 is a review of the

symptomatology, serology and antibiotic treatment. Information on the frequency of late Lyme neuroborreliosis is not provided.

2.2

It is undisputed that polyneuropathy in LB is multifocal and more or less asymmetrical. However, this fact has little to do with the problem at hand. The decisive point is that there are no data on the frequency of polyneuropathy without ACA for Europe.

Only patients with ACA were included in the studies published by Kindstrand et al. 1997 and Kristoferitsch et al. 1988, and cited in the GL. The frequency of peripheral neuropathy in these patients was 64% and 50% respectively. In American studies, polyneuropathy without simultaneous ACA is identified in 36% of patients with late Lyme borreliosis (19–21).

3.3.1

The assertion made in Table 2 of the GL that IgG antibodies occur in the late phase of Lyme borreliosis (late infection) at a frequency of close to 100% is incorrect. Two publications (Hansen and Asbrink 1989, Wilske et al. 1993) are cited in this context, both of which are methodological studies (to improve serological testing procedures) and do not examine at all the frequency of antibodies in the late phase. On the contrary, there is extensive literature proving that antibodies in late Lyme borreliosis are often absent (seronegativity), with a frequency of around 30% (22–61).

The steering group indicated that 40 papers were evaluated and that none of these papers showed seronegativity in late Lyme borreliosis. Reference to the appendix (40 papers) was made.

Short remarks on this appendix are given below:

- Dattwyler et al. 1997. Seronegativity at follow-up (late stage with residual symptoms) was 29%.
- Coyle et al. 1995. The paper refers to 83 patients with LLN. The duration of the disease is not stipulated in the publication. Forty-seven per cent of the patients

had no inflammatory CSF, i.e. no indication of acute Lyme neuroborreliosis, 20% of the patients with confirmed Lyme neuroborreliosis were seronegative

- Luft et al. 1997. 140 patients with recurring LB after antibiotic treatment: seronegativity 29%.
- Lomholt et al. 2000. Follow-up after erythema migrans over an average of 23 months. 41% remained seronegative.
- Eldoen et al. 2001. 25 patients with Lyme neuroborreliosis. The duration of the disease is not stated. (Remark by Dr Berghoff: Pleocytosis and positive borreliosis serology in cerebrospinal fluid do not prove that this was early stage. In 56% of the patients the serology was positive in CSF while being negative in serum).
- Grignolo et al. 2001. 93 LB patients. The duration of the disease is not mentioned in the publication. However, it can be inferred from the context that the disease had already persisted for many weeks. Fifty-one per cent of patients were seronegative.
- Klempner et al. 2001. The data originally intended for the study showed a seronegativity in 25% of the patients. Seronegativity was 51% in the patients ultimately enrolled in the study. – The steering group quoted a passage in which a persisting infection with Bb was not detected because there was no positive pathogen detection. In this context, it must, of course, be taken into consideration that the methods used to detect pathogens have a very low sensitivity.
- Kalish et al. 2001. In actuality, there was always seropositivity in the case of Lyme arthritis. The rate of seronegativity reached 20% in the overall cohort of LB patients with other manifestations.
  - (Table 1, inserted by the steering group, is not from the publication by Kalish et al. 2001, remark by Dr. Berghoff).
- Dinermann et al. 1992. 15 Patients with LB. Monitoring of disease progression late in the disease. 3 patients seronegative, seronegativity also 20%.
- Engstrom et al. 1995. 95 patients. After antibiotic treatment of EM. Monitoring of disease progression for up to one year following treatment. 20% remained seronegative throughout this one-year period.
- Dattwyler et al. 1997. 140 patients. 15% seronegative during the follow-up period of over one year. 30% seronegative at the beginning of the study. Disease duration not specified in the publication. Patients with disseminated early stage

(multiple erythema migrans), AV block, cranial neuropathy, neuroradiculitis for at least 3 months (!)

- Logigian et al. 1999. Agreement with DBG/steering group. 83% seropositive, hence 17% seronegative.
- Nikkilä et al. 1999. 65% seronegativity according to the steering group, 70% according to DBG, in other words no significant difference.

The list of literature on seronegativity (compiled by R. Dersch) does not help in clarifying the issue as it does not contain any quantitative data.

It is particularly disconcerting that the publication by Klemann and Huismans 2009 is not included in the GL under the aspect of seronegativity in late Lyme borreliosis. This important publication on patients with late Lyme borreliosis, proven by pathogen detection, shows seronegativity for IgG AB in 48% of cases.

The publication by Leeflang et al. 2016, a literature survey that points to the possibility of seronegativity in a considerable proportion of cases, should also be mentioned.

Also incomprehensible is the assertion by the steering group that "seronegativity in immunocompetent patients is implausible from a pathophysiological perspective" and that this fundamental consideration does not prompt a special vote to be taken on detecting seronegativity in late Lyme borreliosis. It is obvious that the steering group cannot prove on the basis of scientific literature that IgG antibodies are present in late Lyme borreliosis. It therefore uses (supposedly correct) principles of infection pathophysiology to **make** seronegativity implausible in late Lyme borreliosis.

Once again it should be pointed out that scientific literature does not support the assertion in the guideline text that late Lyme borreliosis is generally seropositive. The GL text refers exclusively to the publications by Hansen and Asbrink 1989 and Wilske et al. 1993. However, these publications do not deal with the frequency of seropositivity in late Lyme borreliosis. Instead they are methodological studies for improving serological testing methods.

3.10

The paper by Kaiser und Rauer 1998, was essentially a methodological study on the detection of intrathecal antibodies. Only patients with neuroborreliosis are mentioned in the study; no distinction is made between the early and late stages. The paper by Halperin et al. 1996 also does not distinguish between the early and late stages.

3.11

It can be gathered from algorithm for early neuroborreliosis (Figure 1 in the GL) that pleocytosis occurs in meningoradiculitis, cranial neuritis, plexus neuritis or mononeuritis multiplex without concomitant meningitis. In this context, the steering group referred to neurology textbooks. Of course, textbooks **without literary sources** cannot replace scientific literature, especially studies. The internationally authoritative textbook "Adams and Victor's Principle of Neurology" states: Lumbar puncture is an essential part of an examination of patients with signs and symptoms of meningitis or of any patient suspected of having meningitis. The online reference book 'UpToDate' also mentions pleocytosis of infectious origin only in connection with meningitis. B. Scheidt's renowned textbook on neurology states: "Cell proliferation indicates an inflammatory reaction of the meninges." In this context, the decisive question is whether inflammation of the parenchyma without simultaneous meningitis leads to pleocytosis. Multiple sclerosis is the prototype of one such constellation, with CSF being inconspicuous in 65% of cases (Rudick RA, Whitaker JN in Neurology/Neurosurgery Update Series, Scheinberg B (Ed), CPEC, Princeton, NJ 1987. Vol 7, p. 1.).

The main publications dealing with this issue are presented below:

Pleocytosis with Encephalitis/Myelitis

Cerebrospinal fluid findings in aquaporin-4 antibody positive neuromyelitis optica: results from 211 lumbar punctures

Jarius S, Paul F, Franciotta D, Ruprecht K, Ringelstein M, Bergamaschi R, Rommer P, Kleiter I, Stich O, Reuss R, Rauer S, Zettl UK, Wandinger KP, Melms A, Aktas O, Kristoferitsch W, Wildemann B. J Neurol Sci. 2011;306(1-2):82-90.

Pleocytosis in 50% of the CSF samples. Mostly a minor case of pleocytosis, 19 cells/ul on average.

CSF pleocytosis and expansion of spinal lesions in Japanese multiple sclerosis with special reference to the new diagnostic criteria

Fukazawa T, Kikuchi S, Miyagishi R, Miyazaki Y, Fukaura H, Yabe I, Hamada T, Tashiro K, Sasaki H. J Neurol. 2005;252(7):824-9.

According to new diagnostic criteria, pleocytosis over 50/mm3 is considered a criterion for exclusion.

CSF characteristics in early-onset multiple sclerosis

Pohl D, Rostasy K, Reiber H, Hanefeld F. Neurology. 2004;63(10):1966-7.

Pleocytosis in 66% of MS cases.

Cerebrospinal fluid pleocytosis in multiple sclerosis patients with lesions showing reduced diffusion

Eisele P, Szabo K, Griebe M, Wolf ME, Hennerici MG, Gass A. Mult Scler. 2014;20(10):1391-5.

Pleocytosis (11–46 cells/ul) is apparently a very early and temporary phenomenon of MS.

With respect to Lyme borreliosis there is no literature on the frequency of pleocytosis with respect to encephalitis, myelitis or neuroradiculitis. Therefore, it cannot be ruled out that, in the case of Lyme neuroborreliosis of the central nervous system and also in other manifestations (plexopathy, neuroradiculitis, cranial neuropathy, neuritis, neuritis multiplex), no pleocytosis will occur in the cerebrospinal fluid.

CSF was pathological in only 25% of cases of facial palsy (Kohler et al. 1999). A study by Belman et al. 1997 found CSF to be pathological in 68% of the cases, for Albasetti et al. 1997 it was 80%, for Pohl et al. 1987 70% with cranial neuropathy, 50% with acute inflammation of the cranial nerve VIII.

3.12

The Borrelia lymphocyte transformation test (LTT) is another tool (indication) for the diagnosis of Lyme borreliosis. The current objections to LTT are based on insufficient specificity. However, this argumentation is inaccurate. The decisive publication by von Baehr et al. 2012 is currently under scrutiny for ostensibly lacking a definition of Lyme borreliosis in the verum group. In fact, the description of LB patients in the publication of von Baehr et al. 2012 is on a par with numerous studies on the serology of Lyme borreliosis. From the papers by von Baehr et al. 2012 and Valentine-Thon et al. 2007, it follows that the sensitivity and specificity of LTT correspond to serology data. Like serological findings, LTT is also an indication for Lyme borreliosis. Both tests prove a resolved Borrelia infection. A false negative result can be expected in 10–20% of the cases when LTT is used, so that a negative LTT result cannot rule out Lyme borreliosis. False positive LTT results are rare and far below 10%. While a positive serological finding only proves a resolved infection, a positive Borrelia LTT (as an indication, not as proof) indicates a current Borrelia infection.

Contrary to the assumption made by the steering group, neuroradiculitis and cranial neuropathy also occur in late Lyme borreliosis.

According to the algorithm for late neuroborreliosis, pleocytosis and increased protein in the cerebrospinal fluid are preconditions for an assumed late neuroborreliosis. This assumption cannot be substantiated by literature for all manifestations of late Lyme

neuroborreliosis. Instead, it can be assumed that the cerebrospinal fluid is inconspicuous in a relevant percentage of cases.

4.2

Section 4.2 is entitled “Presumptive chronic neuroborreliosis”. The term “presumptive” implies a negation. The DBG is of the opinion that chronic neuroborreliosis is identical to late Lyme neuroborreliosis, which has been frequently documented in the literature. In the case of chronic neuroborreliosis, the infection persists, which in turn sustains the disease process. The view of the steering group that the terms “chronic Lyme borreliosis” or “chronic neuroborreliosis” confusingly overlap are not supported by the DBG. Chronic Lyme borreliosis is the persistence of an infection with the symptoms described in the literature for Lyme borreliosis. Chronic Lyme neuroborreliosis refers to a persisting infection with ongoing recurrent or newly occurring neurological symptoms.

Section 4.2 plays down the problem of late Lyme borreliosis and late Lyme neuroborreliosis (identical to chronic Lyme borreliosis and chronic Lyme neuroborreliosis respectively). With respect to various literature references, the GL text states: “The diagnosis of Lyme borreliosis was confirmed in a small proportion of patients; PTLDS in 6%–20%.” The GL text primarily refers to the literature sources Hassett et al. 2009, Ljostad and Mygland 2012, Djukic et al. 2011, and Coumou et al. 2015.

A paper by Hassett et al. examined 240 patients initially assumed to have a persisting Bb infection. The study showed that 60% of the symptoms could not be explained by persisting Lyme borreliosis; however, alternative diagnoses were not given. Nevertheless, a “chronic multi-symptom disease” was more common than in the control group. A publication by Ljostad and Mygland studied 29 patients who attributed their symptoms to chronic Lyme borreliosis (i.e. no medical diagnosis). However, a persisting Bb infection could not be proven in any of the cases. The authors point out that the current diagnostic criteria are controversial in patients with long-term symptoms. The paper by Djukic et al. is the only publication mentioned that deals with Lyme neuroborreliosis. Of the 122 patients examined, nine suffered from acute Lyme borreliosis. One of the 9 patients met the criteria for acute Lyme neuroborreliosis. This patient was treated with ceftriaxone for three weeks. Six months later, however,

headaches and other symptoms (of LNB) reappeared. The authors believe that the data should encourage further studies with new experimental parameters. Out of a group of 95 patients with previous Lyme borreliosis and antibiotic treatment, almost 30% suffered from symptoms without “a demonstrable somatic cause”. Coumou et al. 2015, published a retrospective study of 200 patients who had been admitted with the following diagnoses: Lyme borreliosis, PTLDS, persisting Bb infection despite antibiotic treatment or no indication of Lyme borreliosis. 60% had no Lyme borreliosis, 16% suffered from localised disseminated Lyme borreliosis, 17% had PTLDS, and 8% were diagnosed with persisting Lyme borreliosis. The authors point out that the number of cases of persisting Lyme borreliosis is low and stress that proving or ruling out an association with Bb is often a challenge.

It is not clear why the steering group doubts the existence of chronic Lyme borreliosis on the basis of this literature. It is undisputed that false diagnoses occur in many disease situations. This also applies the false presumption of Lyme borreliosis in cases of disease caused by something else. However, this does not justify trivialising chronic Lyme borreliosis.

It is even more problematic to refer to the paper by Feder et al. 2007, which is not a study, but rather an opinion publication with numerous arbitrary assertions and assumptions that lack a scientific foundation.

The text in the GL about the four categories of chronic Lyme borreliosis can be traced back to the publication by Feder et al. 2007: A Critical Appraisal of “Chronic Lyme Disease”. The authors are the opinion leaders of the IDSA (Infectious Disease Society of America).

The wording chosen in the GL does not correspond to the corresponding passage in the publication by Feder et al. 2007. In fact, the text reads:

“Patients with category 3 have no history of objective clinical findings compatible with Lyme borreliosis. Bb AB are detectable, however chronic subjective symptoms of unclear origin are stated as the reason for the serological testing. Category 3 patients usually only have a vague indication of a Bb infection because the predictive value of a positive serological result in this context is low. Although some clinicians would offer category 3 patients empirical treatment with oral antibiotics for two to four

weeks, these patients should be told that the diagnosis is unconfirmed and it is unlikely that they would benefit from the treatment."

According to the authors, their publication does not refer to the objective manifestations of late Lyme borreliosis, but rather to the imprecisely defined disease situation known as "chronic Lyme borreliosis". According to the authors, this term is used by a small number of general practitioners (often self-designated "Lyme-literate physicians") to describe patients they believe have a persisting B. burgdorferi infection, a disease situation which they suggest requires long-term antibiotic treatment or may even be incurable. Although chronic Lyme borreliosis includes post-Lyme disease syndrome, it also includes a wide range of diseases or symptom complexes for which there is no reproducible or convincing scientific evidence of a connection to a B. burgdorferi infection. Chronic Lyme disease is a diagnosis given to patients in North America and increasingly in Europe who have persistent pain, neurocognitive symptoms, fatigue or any such symptoms with or without clinical or serological evidence of a previous case of early Lyme borreliosis.

The diagnosis (of chronic Lyme disease) is often solely based on a clinical assessment rather than on well-defined clinical criteria and validated laboratory studies. Often the fact that the patients had been in endemic areas is disregarded. Although proponents of the diagnosis "chronic Lyme disease" believe that patients have a persisting Borrelia burgdorferi infection, they require no objective clinical or laboratory evidence of an infection when making the diagnosis. One of the misconceptions (of the proponents) is the unproven and very unlikely assumption that a chronic B. burgdorferi infection may also be present when there is seronegativity. (In other words) false seropositive results often come from dubious laboratories.

Categories of chronic Lyme borreliosis:

The diagnosis of chronic Lyme borreliosis essentially relates to four categories. (The text on category 3 has already been presented above. Even though "chronic Lyme borreliosis" is described as an imprecisely defined disease situation and the term is often used to label misdiagnoses, the authors categorise it into four groups. In addition to category 3 mentioned above, the other categories are of no relevance to the present case, remarks by the DBG).

Statement by the DBG

Although Feder et al. basically deny the existence of chronic Lyme borreliosis, they incomprehensibly break down chronic Lyme borreliosis into categories. It remains unclear whether, contrary to the general statement, they agree with the diagnosis of category 3 chronic Lyme borreliosis; they merely state that some clinicians would offer category 3 patients an empirical treatment of oral antibiotics (obviously for differential therapeutic reasons, remarks by the DBG).

The publication by Feder et al. expresses concern that symptoms of unexplained cause are labelled “chronic Lyme borreliosis” and that antibiotic treatment is carried out on such a basis. On the other hand, they point out that symptoms may persist after antibiotic treatment if Lyme borreliosis is (initially) confirmed. This symptomatology is referred to by the authors as “post-Lyme disease symptoms” or “post-Lyme disease syndromes” if it lasts more than six months. It should be noted that such post-Lyme disease symptoms and “post-Lyme disease syndromes” are usually mild and self-limiting. The publication does not contain any comments or explanations on the pathophysiology of these persisting symptoms; in particular, they are not differentiated from late Lyme borreliosis. Literature references are missing.

It is incomprehensible that, based on this, the GL (Section 4.2.3) recommends considering antibiotic treatment for 14–21 days. Without a clear clinical diagnosis of late Lyme borreliosis, a positive serology (on its own, in line with category 3) does not justify antibiotic treatment.

It is important to note that late Lyme borreliosis cannot be equated with post-Lyme disease symptoms or post-Lyme disease syndromes.

The GL commission should be aware that the publication by Feder et al. 2007 refers to “chronic Lyme borreliosis” and not to “presumptive chronic neuroborreliosis”. Therefore Section 4.2. has no convincing basis and should be deleted.

Essential points in the publication by Feder et al. 2007 are contradictory and deficient in representation and argumentation. It appears that there are differences of opinion among the group of authors and/or the content has not been exhaustively

considered. This particularly applies to category 3. However, a discussion on this matter does not belong in the guideline of the German Society of Neurology.

With respect to Feder's so-called category 3, according to the underlying literature, the symptomatology is "fibromyalgia" and "fatigue" when the borrelia serology is positive. The antibiotic treatment recommended in the guideline is also to be rejected on this basis.

4.3

Post-Lyme syndrome (PLS), post-treatment Lyme disease syndrome (PTLDS), residual symptoms, residual syndrome and residual complaints are synonyms. PLS is not a defined disease (nosological unit). For terminology reasons and due to the progression of the symptoms, it can be assumed that there is a causal link between Lyme borreliosis and PLS. There is no literature on the differentiation between PLS and late Lyme borreliosis.

Post-Lyme syndrome (PLS) and post-treatment Lyme disease syndrome (PTLDS), subsequently introduced in literature, are hypothetical terms with a hypothetical premise. In patients with proven Lyme borreliosis, standard antibiotic therapy did not relieve symptoms. On the contrary, some of the symptoms existing prior to antibiotic treatment persisted. In this context, it was (implicitly) assumed in the literature that standard antibiotic treatment guarantees the elimination of Lyme borreliosis. However, the high therapeutic success of antibiotic treatment of Lyme borreliosis has only been scientifically proven for erythema migrans, i.e. early Lyme borreliosis, and no such evidence in the form of evidence-based studies is available for **the late stage**. There is no evidence-based data on the efficacy of antibiotic treatment, especially for late Lyme borreliosis. In fact, the literature often describes the failure of antibiotic treatment, in particular for late Lyme borreliosis, on the basis of the clinical picture and pathogen detection.

Since the premise (standard antibiotic treatment guarantees elimination of LB) already represents a hypothesis, the conclusion that the persisting LB symptoms following antibiotic treatment represent a (separate) syndrome is also hypothetical. The wording of the GL text that states "PTLDS is to be diagnostically differentiated from symptoms caused by the persistence of reproducing pathogens" is illogical. It is not possible to distinguish between a fact, namely late Lyme borreliosis (persistence of reproducing

pathogens) and a hypothesis (guaranteed cure through antibiotics), since a distinction between fact and hypothesis is not compatible with the laws of logic.

A causal link between Lyme borreliosis and PTLDS is rejected in practiced medicine and in forensic contexts. The hypothetical assumption of PTLDS thus leads to a failure to administer causal (anti-infectious) treatment and to the rejection of the causal link between symptoms and Lyme borreliosis in the field of forensics.

The wording of the GL text "PTLDS is to be diagnostically differentiated from symptoms caused by the persistence of reproducing pathogens" must be replaced by the following wording: "The hypothetically assumed PTLDS cannot be distinguished from late Lyme borreliosis."

In addition, it should be noted that the GL text on PTLDS only lists symptoms which are not related to the nervous system, i.e. which cannot be attributed to Lyme neuroborreliosis.

Section 4.3 should therefore only show that PTLDS is a hypothesis and cannot be distinguished from late Lyme neuroborreliosis. All other findings in Section 4.3 of the GL text are "suggestively misleading" and contain no medically or forensically relevant information.

4.3.5

The publication by Klempner et al. 2001 does not deal with PTLDS but with the efficacy of antibiotic after-treatment of chronically persisting symptoms of Lyme borreliosis (after prior initial antibiotic treatment) in seropositive and seronegative patients. There was a significant disease burden prior to post-treatment. During post-treatment, 2 g of ceftriaxone was administered for four weeks, followed by doxycycline for two months. The authors used the term "chronic Lyme borreliosis", but not terms such as "post-Lyme syndrome". – The initial antibiotic treatment consisted of an average of three antibiotic treatment cycles with a total duration of approximately 50 to 65 days. The original disease consisted mainly of erythema migrans or acute neuroborreliosis. The paper by Kaplan et al. 2003 corresponds in design and cohorts studied to the publication by Klempner et al. 2001, but essentially refers to cognitive and social functions of mood and pain. The term used is "post-treatment chronic Lyme

disease" (PTCLD) and not PLS. In a paper by Krupp et al. 2003, antibiotic treatment led to a significant improvement in fatigue, but not in cognitive disorders. However, the work of Kaplan et al. 2003 and Krupp et al. 2003 showed improvements in cognitive performance based on patient self-assessments. The publication by Fallon et al. 2008 also does not deal with PTLDS, but with the efficacy of antibiotic after-treatment for encephalopathy. The authors insist that treatment strategies with a lasting effect are needed in cases of persisting cognitive impairment.

PTLDS is a hypothesis. There is no literature on the differentiation between PTLDS and late Lyme borreliosis. Late Lyme borreliosis can occur as the primary disease, i.e. without a preceding early stage. Antibiotic treatment is indicated if late Lyme borreliosis cannot be ruled out based on the available data (medical history, physical examination, medical and technical data, and differential diagnosis).

4.4

Encephalopathy in late Lyme disease has been clearly documented by the literature.

Encephalopathy is the term used to describe impaired cognition and mental disorders that can lead to significant impairments in social functions. Education may be affected in school-age children. The use of the term "encephalopathy" in connection with PTLDS is unfounded as PTLDS is not defined as a disease.

It is undisputed that the pathogenesis of Lyme encephalopathy is unknown. The steering group's reference to encephalitis or a "toxic-metabolic" encephalopathy is illogical. Specifying alleged causes when pathogenesis is unexplained once again contradicts the laws of logic. The steering group's reference to other authors using the term Lyme encephalopathy in connection with cognitive symptoms in PTLDS patients is also incomprehensible since PTLDS is a hypothesis and this argumentation (in the case of unexplained pathogenesis) would also be illogical.

5.3

The efficacy of antibiotic treatment of late Lyme neuroborreliosis is very limited as indicated by the publications cited. Equating antibiotic treatment during the early stage with that during the late stage is wrong and arbitrary. The literature does not provide evidence that the efficacy of antibiotic treatment in the early stage can be transferred to the late stage; this applies in particular to the duration of treatment. It is also incorrect to claim that the cited literature showed no evidence of treatment failure when beta-lactams or doxycycline were administered for two to three weeks for late Lyme neuroborreliosis. In the publication by Hansen and Lebech, 94% of the examined patients suffered from early Lyme borreliosis, i.e. only about 10 patients had a late stage form of the disease. The type of antibiotic treatment chosen to treat the late stage is not deducible from the publication. Kaiser's paper looks at 15 patients with chronic neuroborreliosis, (only) 66% of whom were cured by antibiotic treatment. Moreover, the text correctly states that there are no evidence-based studies on the antibiotic treatment of late Lyme borreliosis. It is illogical to cite the lack of literature on the benefits of prolonged antibiotic treatment as an argument for limiting antibiotic treatment to two to three weeks. The recommended time limit for antibiotic treatment is arbitrary and the assumed efficacy hypothetical. Therefore, based on the current state of knowledge, the type and duration of antibiotic treatment for late Lyme neuroborreliosis must depend on the clinical course of the disease.

5.5

The recommendation (Table 5 of the GL) regarding antibiotic treatment of late Lyme borreliosis cannot be substantiated by the literature. The data were arbitrarily taken from early-stage studies.

6

Appendix 2

The IDSA has only made criteria proposals for PTLDS; in fact, there are currently no recognised criteria. PTLDS is a hypothesis and not defined as a disease. Therefore, it cannot be differentiated from late-stage neuroborreliosis.

List of references

1

1. Asch ES, Bujak DI, Weiss M, Peterson MG, Weinstein A. Lyme disease: an infectious and postinfectious syndrome. J Rheumatol. 1994;21:454-56.
2. Ziska MH, Donta ST, Demarest FC. Physician preferences in the diagnosis and treatment of Lyme disease in the United States. Infection. 1996;24:182-6.
3. Hassler D. Langzeitbeobachtungen zum Krankheitsbild der Lyme-Borreliose in einem Endemiegebiet [Habilitation]. Erlangen: Universität Erlangen; 1997.
4. Logigian EL, Kaplan RF, Steere AC. Chronic neurologic manifestation of Lyme disease. N Engl J Med. 1990;323:1438-44.
5. Fallon BA, Nields JA. Lyme disease: a neuro-psychiatric illness. Am J Psychiatry. 1994;151:1571-83.
6. Culp RW, Eichenfield AH, Davidson RS, Drummond DS, Christofersen MR, Goldsmith DP. Lyme-arthritis in children: an orthopedic perspective. J Bone Joint Surg AM. 1987;69:96-9.
7. Pfister HW, Einhäupl KM, Wilske B, Preac-Mursic V. Bannwarth-syndrome and the enlarged neurological spectrum of arthropod-born Borreliosis. Zentralblatt Bakt Hyg A. 1986;263:343-7.
8. Ogrinc K, Logar M, Lotric-Furlan S, Cerar D, Ruzic-Sabljic E, Strle F. Doxycycline versus ceftriaxone for the treatment of patients with chronic Lyme borreliosis. Wien Klin Wochenschr. 2006;118:696-701.
9. Stanek G, Flamm H, Groh V, Hirschl A, Kristoferitsch W, Neumann R, Schmutzhard E, Wewalka G. Epidemiology of Borrelia infections in Austria. Zentralbl Bakteriol Mikrobiol Hyg A. 1986;263:442-9.

10. Asbrink E, Hovmark A. Early and late cutaneous manifestations in Ixodes-borne borreliosis (erythema migrans borreliosis, Lyme borreliosis). Ann NY Acad Sci. 1988;539:4-15.

11. Stanek G, Pletschette M, Flamm H, Hirschl AM, Aberer E, Kristoferitsch W, Schmutzhard E. European Lyme borreliosis. Ann NY Acad Sci. 1988;539:472-81.

12. Koedel U, Fingerle V, Pfister HW. Lyme neuroborreliosis-epidemiology, diagnosis and management. Nat Rev Neurol. 2015;11(8):446-56.

13. Halperin JJ. Nervous system lyme disease: diagnosis and treatment. Rev Neurol Dis. 2009;6(1):4-12.

14. Fülöp B, Poggensee G. Epidemiological situation of Lyme borreliosis in Germany: surveillance data from six Eastern German States, 2002 to 2006. Parasitol Res. 2008;103 Suppl 1:S117-20.

15. Lovett JK, Evans PH, O'Connell S, Gutowski NJ. Neuroborreliosis in the South West of England. Epidemiol Infect. 2008;136(12):1707-11.

16. Christova I, Komitova R. Clinical and epidemiological features of Lyme borreliosis in Bulgaria. Wien Klin Wochenschr. 2004;116(1-2):42-6.

17. Smith R, O'Connell S, Palmer S. Lyme disease surveillance in England and Wales, 1986-1998. Emerg Infect Dis. 2000;6(4):404-7.

18. Klemann W, Huismans BT. Patienten mit Erreger-Direktnachweis bei chronischer Lyme-Borreliose: Klinik, Labordiagnostik, Antibiotika-Therapie und Krankheitsverlauf – Eine retrospektive Studie. Umwelt-Medizin-Gesellschaft. 22, 2/2009.

2.2

19. Kindstrand E, Nilsson BY, Hovmark A, Pirskanen R, Asbrink, E. Peripheral neuropathy in acrodermatitis chronica atrophicans – a late Borrelia amnifestation. Acta Neurol Scand. 1997;95:338-45.

20. Kristoferitsch W, Sluga E, Graf M, Partsch H, Neumann R, Stanek G, Budka H. Neuropathy associated with acrodermatitis chronica atrophicans. Clinical and morphological features. Ann NY Acad Sci. 1988;539:35-45.

21 Halperin JJ, Little BW, Coyle PK, Dattwyler RJ. Lyme disease: cause of a treatable peripheral neuropathy. Neurology. 1987;37(11):1700-6.

3.3.1

22. Klemann W, Huismans BD. Patienten mit Erreger-Direktnachweis bei chronischer Lyme-Borreliose: Klinik, Labordiagnostik, Antibiotika-Therapie und Krankheitsverlauf – Eine retrospektive Studie. Umwelt-Medizin-Gesellschaft. 22, 2/2009.

23. Lawrence C, Lipton RB, Lowy FD, Coyle PK. Seronegative chronic relapsing neuroborreliosis. Eur Neurol. 1995;35(2):113-7.

24. Keller TL, Halperin JJ, Whitman M. PCR detection of Borrelia burgdorferi DNA in cerebrospinal fluid of Lyme neuroborreliosis patients. Neurology. 1992;42 (1):32-42.

25. Tylewska-Wierzbanowska S, Chmielewski T. Limitation of serological testing for Lyme borreliosis: evaluation of ELISA and western blot in comparison with PCR and culture methods. Wien Klein Wochenschr. 2002 Jul 31;114(13-14): 601-5.

26. Mouritsen CL, Wittwer CT, Litwin CM, Yang L, Weis JJ, Martins TB, Jaskowski TD, Hill HR. Polymerase chain reaction detection of Lyme disease: correlation with clinical manifestations and serologic responses. AM J Clin Pathol. 1996 May;105(5):647-54.

27. Hassler D, Zöller L, Haude M, Hufnagel HD, Heinrich F, Sonntag HG. Cefotaxime versus penicillin in the late stage of Lyme disease – prospective, randomized therapeutic study. Infection. 1990;18(1):16-20.

28. Klempner MS, Hu LT, Evans J, Schmid CH, Johnson GM, Trevino RP, Norton D, Levy L, Wall D, McCall J, Kosinski M, Weinstein A. Two controlled trials of antibiotic treatment in patients with persistent symptoms and a history of Lyme disease. N Engl J Med. 2001;346(2):85-92.

29. Preac-Mursic V, Weber K, Pfister HW, Wilske B, Gross B, Baumann A, Prokop J. Survival of Borrelia burgdorferi in antibiotically treated patients with Lyme borreliosis. Infection. 1989;17(6):355-9.

30. Feder HM Jr, Gerber MA, Luger SW, Ryan RW. Persistence of serum antibodies to Borrelia burgdorferi in patients treated for Lyme disease. Clin Infect Dis. 1992;15(5):788-93.

31. Preac-Mursic V, Pfister HW, Spiegel H, Burk R, Wilske B, Reinhardt S, Böhmer R. First isolation of Borrelia burgdorferi from an iris biopsy. J Clin Neuroophthalmol. 1993;13(3):155-61.

32. Luft BJ, Dattwyler RJ, Johnson RC, Luger SW, Bosler EM, Rahn DW, Masters EJ, Grunwaldt E, Gadgil SD. Azithromycin compared with amoxicillin in the treatment of Erythema migrans. A double-blind, randomized, controlled trial. Ann Intern Med. 1996;124(9):785-91.

33. Preac Mursic V, Marget W, Busch U, Pleterski Rigler D, Hagl S. Kill kinetics of Borrelia burgdorferi and bacterial findings in relation to the treatment of Lyme borreliosis. Infection. 1996;24(1):9-16.

34. Branigan P, Rao J, Rao J, Gerard H, Hudson A, Williams W, Arayssi T, Pando J, Bayer M, Rothfuss S, Clayburne G, Sieck M, Schumacher HR. PCR evidence for Borrelia burgdorferi DNA in synovium in absence of positive serology. America College of Rheumatology. 1997;40(9):270.

35. Logigian EL, Kaplan RF, Steere AC. Successful treatment of Lyme encephalopathy with intravenous ceftriaxone. J Infect Dis. 1999;180(2):377-83.

36. Oksi J, Marjamaki M, Nikoskelainen J, Viljanen MK. Borrelia burgdorferi detected by culture and PCR in clinical relapse of disseminated Lyme borreliosis. Ann Med. 1999;31(3):225-32.

37. Klemann W, Huismans BD. Patienten mit Erreger-Direktnachweis bei chronischer Lyme-Borreliose: Klinik, Labordiagnostik, Antibiotika-Therapie und Krankheitsverlauf – Eine retrospektive Studie. Umwelt-Medizin-Gesellschaft. 22, 2/2009.

38. Lawrence C, Lipton RB, Lowy FD, Coyle PK. Seronegative chronic relapsing neuroborreliosis. Eur Neurol. 1995;35(2):113-7.

39. Tylewska-Wierzbanowska S, Chmielewski T. Limitation of serological testing for Lyme borreliosis: evaluation of ELISA and western blot in comparison with PCR and culture methods. Wien Klein Wochenschr. 2002 Jul 31;114(13-14): 601-5.

40. Mouritsen CL, Wittwer CT, Litwin CM, Yang L, Weis JJ, Martins TB, Jaskowski TD, Hill HR. Polymerase chain reaction detection of Lyme disease: correlation with clinical manifestations and serologic responses. AM J Clin Pathol. 1996 May;105(5):647-54.

41. Klempner MS, Hu LT, Evans J, Schmid CH, Johnson GM, Trevino RP, Norton D, Levy L, Wall D, McCall J, Kosinski M, Weinstein A. Two controlled trials of antibiotic treatment in patients with persistent symptoms and a history of Lyme disease. N Engl J Med. 2001;346(2):85-92.

42. Preac-Mursic V, Weber K, Pfister HW, Wilske B, Gross B, Baumann A, Prokop J. Survival of Borrelia burgdorferi in antibiotically treated patients with Lyme borreliosis. Infection. 1989;17(6):355-9.

43. Häupl T, Hahn G, Rittig M, Krause A, Schoerner C, Schönherr U, Kalden JR, Burmester GR. Persistence of Borrelia burgdorferi in ligamentous tissue from a patient with chronic Lyme borreliosis. Arthritis Rheum. 1993;36(11):1621-6.

44. Luft BJ, Dattwyler RJ, Johnson RC, Luger SW, Bosler EM, Rahn DW, Masters EJ, Grunwaldt E, Gadgil SD. Azithromycin compared with amoxicillin in the treatment of Erythema migrans. A double-blind, randomized, controlled trial. Ann Intern Med. 1996;124(9):785-91.

45. Preac Mursic V, Marget W, Busch U, Pleterski Rigler D, Hagl S. Kill kinetics of Borrelia burgdorferi and bacterial findings in relation to the treatment of Lyme borreliosis. Infection. 1996;24(1):9-16.

46. Ziska MH, Donta ST, Demarest FC. Physician preferences in the diagnosis and treatment of Lyme disease in the United States. Infection. 1996;24:182-6.

47. Hassler D. Langzeitbeobachtungen zum Krankheitsbild der Lyme-Borreliose in einem Endemiegebiet [Habilitation]. Erlangen: Universität Erlangen; 1997.

48. von Baehr V, Liebenthal C, Gaida B, Schmidt FP, von Baehr R, Volk HD. Untersuchungen zur diagnostischen Wertigkeit des Lymphozytentransformationstestes bei Patienten mit Borreliose. J Lab Med. 2007;31(3):149-58.

49. Breier F, Khanakah G, Stanek G, Kunz G, Aberer E, Schmidt B, Tappeiner G. Isolation and polymerase chain reaction typing of Borrelia afzelii from a skin lesion in a seronegative patient with generalized ulcerating bullous lichen sclerosus et atrophicus. Br J Dermatol. 2001;144(2):387-92.

50. Dattwyler RJ, Volkman DJ, Luft BJ, Halperin JJ, Thomas J, Glightly MG. Seronegative Lyme disease. Dissociation of specific T- and B-lymphocyte responses to Borrelia burgdorferi. N Engl J Med. 1988;319(22):1441-6.

51. Dejmkova H, Hulinska D, Tegzova D, Pavelka K, Gatterova J, Vavrik P. Seronegative Lyme arthritis caused by Borrelia garinii. Clin Rheumatol. 2002;21(4):330-4.

52. Wang P, Hilton E. Contribution of HLA alleles in the regulation of antibody production in Lyme disease. Front Biosci. 2001;6:810-6.

53. Grignolo MC, Buffrini L, Monteforte P, Rovetta G. [Reliability of a polymerase chain reaction (PCR) technique in the diagnosis of Lyme borreliosis]. Med. 2001;92(1):29-33.

54. Honegr K, Hulinska D, Dostal V, Gebousky P, Hankova E, Horacek J, Vyslouzil J. [Persistence of Borrelia burgdorferi sensu lato in patients with Lyme borreliosis]. Epidemiol Mikrobiol Imunol. 2001;50(1):10-6.

55. Coyle PK, Schutzer SE, Deng Z, Krupp LB, Belman AL, Benach JL, Luft BJ. Author Detection of Borrelia burgdorferi-specific antigen in antibody-negative cerebrospinal fluid in neurologic Lyme disease. Neurology. 1995;45(11):2010-5.

56. Breier F, Klade H, Stanek G, Poitschek C, Kirnbauer R, Dorda W, Aberer E. Lymphoproliferation response to Borrelia burgdorferi in circumscribed scleroderma. British Journal of Dermatology. 1996;134:285-91.

57. Huppertz HI, Mösbauer S, Busch DH, Karch H. Lymphoproliferative responses to Borrelia burgdorferi in the diagnosis of Lyme arthritis in children and adolescent. Eur J Pediatr. 1996;155:297-302.

58. Dinermann H, Steere AC. Lyme disease associated with fibromyalgie. Annals of Internal medicine. 1992;117:281-5.

59. Mikkilä H, Karma A, Viljanen M, Seppälä I. The laboratory diagnosis of ocular Lyme borreliosis. Graefes Arch Clin Exp Ophthalmol. 1999;237(3):225-30.

60. Bil-Lula I, Matuszek P, Pfeiffer T, Wozniak M. Lyme Borreliosis – the Utility of Improved Real-Time PCR Assay in the Detection of Borrelia burgdorferi Infections. Adv Clin Exp Med. 2015;24(4):663-70.

61. Kalish RA, Kaplan RF, Taylor E, Jones-Woodward L, Workman K, Steere AC. Evaluation of study patients with Lyme disease, 10-20-year follow-up. J Infect Dis. 2001;183(3):453-60.

3.4

62. Reiber H, Ressel CB, Spreer A. Diagnosis of neuroborreliosis – Improved knowledge base for qualified antibody analysis and cerebrospinal fluid data pattern related interpretations. Neurology, Psychiatry and Brain Research. 2013;19(4):159-69.

3.11

63. von Baehr V, Liebenthal C, Gaida B, Schmidt FP, von Baehr R, Volk HD. Untersuchungen zur diagnostischen Wertigkeit des

Lymphozytentransformationstestes bei Patienten mit Borreliose. J Lab Med. 2007;31(3):149-58.

64. Valentine-Thon E, Ilsemann K, Sandkamp M. A novel lymphocyte transformation test (LTT-MELISA®) for Lyme borreliosis. Diagn Microbiol Infect Dis. 2007;57:27-34.

65. Ogrinc K, Lusa L, Lotric-Furlan S, Bogovic P, Stupica D, Cerar T, Ruzic-Sabljic E, Strle F. Course and Outcome of Early European Lyme Neuroborreliosis (Bannwarth Syndrome): Clinical and Laboratory Findings. Clin Infect Dis. 2016;63(3):346-53.

66. Wormser GP, Dattwyler RJ, Shapiro ED, Halperin JJ, Steere AC, Klempner MS, Krause PJ, Bakken JS, Strle F, Stanek G, Bockenstedt L, Fish D, Dumler JS, Nadelman RB. The clinical assessment, treatment, and prevention of lyme disease, human granulocytic anaplasmosis, and babesiosis: clinical practice guidelines by the Infectious Diseases Society of America. Clin Infect Dis. 2006;43(9):1089-134.

67. Wormser GP, Weitzner E, McKenna D, Nedalman RB, Scavarda C, Molla I, Dornbush R, Visintainer P, Nowakowski J. Long-term assessment of health-related quality of life in patients with culture-confirmed early Lyme disease. Clin Infect Dis. 2015;61(2):244-7.

68. Wills AB, Spaulding AB, Adjemian J, Prevots DR, Turk SP, Williams C, Marques A. Long-term Follow-up of Patients With Lyme Disease: Longitudinal Analysis of Clinical and Quality-of-life Measures. Clin Infect Dis. 2016;62(12);1546-51.

69. Weitzner E, McKenna D, Nowakowski J, Scavarda C, Dornbush R, Bittker S, Cooper D, Nadelman RB, Visintainer P, Schwartz I, Wormser GP. Long-term Assessment of Post-Treatment Symptoms in Patients With Culture-Confirmed Early Lyme Disease. Clin Infect Dis. 2015;61(12):1800-6.

4.4

70. Halperin JJ. Neuroborreliosis. Am J Med. 1995;98(4A):52S-56S; discussion 56S-59S.

71. Bujak DI, Weinstein A, Dornbush RL. J Rheumatol. Clinical and neurocognitive features of the post Lyme syndrome. 1996;23(8):1392-7.

72. Logigian EL, Kaplan RF, Steere AC. Successful treatment of Lyme encephalopathy with intravenous ceftriaxone. J Infect Dis. 1999;180(2):377-83.

73. Logigian EL, Johnson KA, Kijewski MF, Kaplan RF, Becker JA, Jones KJ, Garada BM, Holman BL, Steere AC. Reversible cerebral hypoperfusion in Lyme encephalopathy. Neurology. 1997;49(6):1661-70.

74. Klemann W, Huismans BD. Patienten mit Erreger-Direktnachweis bei chronischer Lyme-Borreliose: Klinik, Labordiagnostik, Antibiotika-Therapie und Krankheitsverlauf – Eine retrospektive Studie. Umwelt-Medizin-Gesellschaft. 22, 2/2009.

75. Keller TL, Halperin JJ, Whitman M. PCR detection of Borrelia burgdorferi DNA in cerebrospinal fluid of Lyme neuroborreliosis patients. Neurology. 1992;42 (1):32-42.

76. Logigian EL, Kaplan RF, Steere AC. Chronic neurologic manifestations of Lyme disease. N Engl J Med. 1990;323(21):1438-44.

77. Klempner MS, Hu LT, Evans J, Schmid CH, Johnson GM, Trevino RP, Norton D, Levy L, Wall D, McCall J, Kosinski M, Weinstein A. Two controlled trials of antibiotic treatment in patients with persistent symptoms and a history of Lyme disease. N Engl J Med. 2001;346(2):85-92.

78. Kaplan RF, Trevino RP, Johnson GM, Levy L, Dombush R, Hu LT, Evans J, Weinstein A, Schmid CH, Klempner MS. Cognitive function in post-treatment Lyme disease: do additional antibiotics help? Neurology. 2003;60(12):1916-22.

79. Fallon BA, Keilp JG, Corbera KM, Petkova E, Britton CB, Dwyer E, Slavov I, Cheng J, Dobkin J, Nelson DR, Sackeim HA. A randomized, placebo-controlled trial of repeated IV antibiotic therapy for Lyme encephalopathy. Neurology. 2008;70(13):992-1003.

80. Shadick NA, Phillips CB, Logigian EL, Steere AC, Kaplan RF, Berardi VP, Duray PH, Larson MG, Wright EA, Ginsburg KS, Katz JN, Liang MH. The long-term clinical outcomes of Lyme disease. A population-based retrospective cohort study. Ann Intern Med. 1994;121(8):560-7.

81. McAuliffe P, Brassard MR, Fallon B. Memory and executive functions in adolescents with posttreatment Lyme disease. Appl Neuropsychol. 2008;15(3):208-19.

82. Bloom BJ, Wyckoff PM, Meissner HC, Steere AC. Neurocognitive abnormalities in children after classic manifestations of Lyme disease. Pediatr Infect Dis J. 1998;17(3):189-96.

83. Krupp LB, Hyman LG, Grimson R, Coyle PK, Melville P, Ahnn S, Dattwyler R, Chandler B. Study and treatment of post Lyme disease (STOP-LD): a randomized double masked clinical trial. Neurology. 2003;60(12):1923-30.

84. Halperin JJ, Pass HL, Anand AK, Luft BJ, Volkman DJ, Dattwyler RJ. Nervous system abnormalities in Lyme disease. Ann NY Acad Sci. 1988;539, 24-34.

85. Tager FA, Fallon BA, Keilp J, Rissenberg M, Jones CR, Liebowitz MR. A controlled study of cognitive deficits in children with chronic Lyme disease. J Neuropsychiatry Clin Neurosci. 2001;13(4):500-7.

86. Fallon BA, Lipkin RB, Corbera KM, Yu S, Nobler MS, Keilp JG, Petkova E, Lisanby SH, Moeller JR, Slavov I, Van Heertum R, Mensh BD, Sackeim HA. Regional cerebral blood flow and metabolic rate in persistent Lyme encephalopathy. Arch Gen Psychiatry. 2009;66(5):554-63.

87. Krupp LB, Masur D, Schwartz J, Coyle PK, Langenbach LJ, Fernquist SK, Jandorf L, Halperin JJ. Cognitive functioning in late Lyme borreliosis. Arch Neurol. 1991;48(11):1125-9.

88. Eikeland R, Ljoesstad U, Mygland A, Herofson K, Loehaugen GC. European neuroborreliosis: neuropsychological findings 30 months post-treatment. Eur J Neurol. 2012;19(3):480-7.

5

89. Hansen K, Lebech AM. The clinical and epidemiological profile of Lyme neuroborreliosis in Denmark 1985–1990. A prospective study of 187 patients with Borrelia burgdorferi specific intrathecal antibody production. Brain. 1992;115(2):399-423.

90. Kaiser R. Variable CSF findings in early and late Lyme neuroborreliosis: A follow-up study in 47 patients. J Neurol. 1994;242:26-36.

91. Bujak DI, Weinstein A, Dornbush RL. Clinical and neurocognitive features of the post Lyme syndrome. J Rheumatol. 1996;23(8):1392-7.

92. Logigian EL, Kaplan RF, Steere AC. Successful treatment of Lyme encephalopathy with intravenous ceftriaxone. J Infect Dis. 1999;180(2):377-83.

93. Cimperman J, Maraspin V, Lotric-Furlan S, Ruzić-Sabljić E, Strle F. Lyme meningitis: a one-year follow up controlled study. Wien Klin Wochenschr. 1999;111(22-23):961-3.

94. Dieterle L, Kubina FG, Staudacher T, Büdingen HJ. Neuroborreliose oder Bandscheibenvorfall? [Neuro-borreliosis or intervertebral disk prolapse?]. Dtsch Med Wochenschr. 1989;114(42):1602-6.

95. Rohacova H, Hancil J, Hulinska D, Mailer H, Havlik J. Ceftriaxone in the treatment of Lyme neuroborreliosis. Infection. 1996;24(1):88-90.

96. Nanagara R, Duray PH, Schumacher HR Jr. Ultrastructural demonstration of spirochetal antigens in synovial fluid and synovial membrane in chronic Lyme disease: possible factors contributing to persistence of organisms. Hum Pathol. 1996;27(10):1025-34.

97. Oksi J, Marjamaki M, Nikoskelainen J, Viljanen MK. Borrelia burgdorferi detected by culture and PCR in clinical relapse of disseminated Lyme borreliosis. Ann Med. 1999;31(3):225-32.

98. Klemann W, Huismans BD. Patienten mit Erreger-Direktnachweis bei chronischer Lyme-Borreliose: Klinik, Labordiagnostik, Antibiotika-Therapie und Krankheitsverlauf – Eine retrospektive Studie. Umwelt-Medizin-Gesellschaft. 22, 2/2009.

99. Nocton JJ, Dressler F, Rutledge BJ, Rys PN, Persing DH, Steere AC. Detection of Borrelia burgdorferi DNA by polymerase chain reaction in synovial fluid from patients with Lyme arthritis. N Engl J Med. 1994;330(4):229-34.

100. Lawrence C, Lipton RB, Lowy FD, Coyle PK. Seronegative chronic relapsing neuroborreliosis. Eur Neurol. 1995;35(2):113-7.

101. Nocton JJ, Bloom BJ, Rutledge BJ, Persing DH, Logigian EL, Schmid CH, Steere AC. Detection of Borrelia burgdorferi DNA by polymerase chain reaction in cerebrospinal fluid in Lyme neuroborreliosis. J Infect Dis. 1996;174(3):623-7.

102. Priem S, Burmester GR, Kamradt T, Wolbart K, Rittig MG, Krause A. Detection of Borrelia burgdorferi by polymerase chain reaction in synovial membrane, but not in synovial fluid from patients with persisting Lyme arthritis after antibiotic therapy. Ann Rheum Dis. 1998;57(2):118-21.

103. Pícha D, Moravcová L, Holečková D, Žďárský E, Valešová M, Marešová V, Hercogová J, Vanousová D. Examination of specific DNA by PCR in patients with different forms of Lyme borreliosis. Report. International Journal of Dermatology. 2008;47:1004-10.

104. Mursic VP, Wannder G, Reinhardt S, Wilske B, Busch U, Marget W. Formation and cultivation of Borrelia burgdorferi spheroplast-L-form variants. Infection. 1996;24(3):218-26.

105. Steere AC, Green J, Schoen RT, Taylor E, Hutchinson GJ, Rahn DW, Malawista SE. Successful parenteral penicillin therapy of established Lyme arthritis. N Engl J Med. 1985;312(14):869-74.

106. Hassler D, Zöller L, Haude M, Hufnagel HD, Heinrich F, Sonntag HG. Cefotaxime versus penicillin in the late stage of Lyme disease – prospective, randomized therapeutic study. Infection. 1990;18(1):16-20.

107. Steere AC, Levin RE, Molloy PJ, Kalish RA, Abraham JH 3rd, Liu NY, Schmid CH. Treatment of Lyme arthritis. Arthritis Rheum. 1994;37(6):878-88.

108. Valesová H, Mailer J, Havlik J, Hulinská D, Hercogová J. Long-term results in patients with Lyme arthritis following treatment with ceftriaxone. Infection. 1996;24(1):98-102.

109. Logigian EL, Kaplan RF, Steere AC. Chronic neurologic manifestations of Lyme disease. N Engl J Med. 1990;323(21):1438-44.

110. Klempner MS, Hu LT, Evans J, Schmid CH, Johnson GM, Trevino RP, Norton D, Levy L, Wall D, McCall J, Kosinski M, Weinstein A. Two controlled trials of antibiotic treatment in patients with persistent symptoms and a history of Lyme disease. N Engl J Med. 2001;346(2):85-92.

111. Oksi J, Nikoskelainen J, Hiekkanen H, Lauhio A, Peltomaa M, Pitkäranta A, Nyman D, Granlund H, Carlsson SA, Seppälä I, Valtonen V, Viljanen M. Duration of antibiotic treatment in disseminated Lyme borreliosis: a double-blind, randomized, placebo-controlled, multicenter clinical trial. Eur J Clin Microbiol Infect Dis. 2007;26:571-81.

112. Fallon BA, Keilp JG, Corbera KM, Petkova E, Britton CB, Dwyer E, Slavov I, Cheng J, Dobkin J, Nelson DR, Sackeim HA. A randomized, placebo-controlled trial of repeated IV antibiotic therapy for Lyme encephalopathy. Neurology. 2008;70(13):992-1003.

113. Preac-Mursic V, Weber K, Pfister HW, Wilske B, Gross B, Baumann A, Prokop J. Survival of Borrelia burgdorferi in antibiotically treated patients with Lyme borreliosis. Infection. 1989;17(6):355-9.

114. Pfister HW, Preac-Mursic V, Wilske B, Schielke E, Sörgel F, Einhäupl KM. Randomized comparison of ceftriaxone and cefotaxime in Lyme neuroborreliosis. J Infect Dis. 1991;163(2):311-8.

115. Feder HM Jr, Gerber MA, Luger SW, Ryan RW. Persistence of serum antibodies to Borrelia burgdorferi in patients treated for Lyme disease. Clin Infect Dis. 1992;15(5):788-93.

116. Preac-Mursic V, Pfister HW, Spiegel H, Burk R, Wilske B, Reinhardt S, Böhmer R. First isolation of Borrelia burgdorferi from an iris biopsy. J Clin Neuroophthalmol. 1993;13(3):155-61.

117. Klempner MS, Noring R, Rogers RA. Invasion of human skin fibroblasts by the Lyme disease spirochete, Borrelia burgdorferi. J Infect Dis. 1993;167(5):1074-81.

118. Häupl T, Hahn G, Rittig M, Krause A, Schoerner C, Schönherr U, Kalden JR, Burmester GR. Persistence of Borrelia burgdorferi in ligamentous tissue from a patient with chronic Lyme borreliosis. Arthritis Rheum. 1993;36(11):1621-6.

119. Chancellor MB, McGinnis DE, Shenot PJ, Kiilholma P, Hirsch IH. Urinary dysfunction in Lyme disease. J Urol. 1993;149(1):26-30.

120. Liu An. [Lyme disease in China and its ocular manifestations]. Zhonghua Yan Ke Za Zhi. 1993; 29(5):271-3.

121. Bradley JF, Johnson RC, Goodman JL. The persistence of spirochetal nucleic acids in active Lyme arthritis. Ann Intern Med. 1994;120(6):487-9.

122. Asch ES, Bujak DI, Weiss M, Peterson MG, Weinstein A. Lyme disease: an infectious and postinfectious syndrome. J Rheumatol. 1994;21(3):454-61.

123. Shadick NA, Phillips CB, Logigian EL, Steere AC, Kaplan RF, Berardi VP, Duray PH, Larson MG, Wright EA, Ginsburg KS, Katz JN, Liang MH. The long-term clinical outcomes of Lyme disease. A population-based retrospective cohort study. Ann Intern Med. 1994;121(8):560-7.

124. Steere AC. Musculoskeletal manifestations of Lyme disease. Am J Med. 1995;98(4A):44-8; discussion 48S-51S.

125. Luft BJ, Dattwyler RJ, Johnson RC, Luger SW, Bosler EM, Rahn DW, Masters EJ, Grunwaldt E, Gadgil SD. Azithromycin compared with amoxicillin in the treatment of Erythema migrans. A double-blind, randomized, controlled trial. Ann Intern Med. 1996;124(9):785-91.

126. Bayer ME, Zhang L, Bayer MH. Borrelia burgdorferi DNA in the urine of treated patients with chronic Lyme disease symptoms. A PCR study of 97 cases. Infection. 1996;24(5):347-53.

127. Aberer E, Kersten A, Klade H, Potschek C, Jurecka W. Heterogeneity of Borrelia burgdorferi in the skin. Am J Dermatopathol. 1996;18(6):571-9.

128. Oksi J, Kalimo H, Marttila RJ, Marjamäki M, Sonninen P, Nikoskelainen J, Viljanen MK. Inflammatory brain changes in Lyme borreliosis. A report on three patients and review of literature. Brain. 1996;119(6):2143-54.

129. Preac Mursic V, Marget W, Busch U, Pleterski Rigler D, Hagl S. Kill kinetics of Borrelia burgdorferi and bacterial findings in relation to the treatment of Lyme borreliosis. Infection. 1996;24(1):9-16.

130. Nowakowski J, Nadelmann RB, Sell R, McKenna D, Cavaliere LF, Holmgren D, Gaidici A, Wormser GP. Long-term follow-up of patients with culture-confirmed Lyme disease. Am J Med. 2003;115(2):91-6.

131. Wormser GP, Ramanathan R, Nowakowski J, McKenna D, Holmgren D, Visintainer P, Dornbush R, Singh B, Nadelman RB. Duration of antibiotic therapy for early Lyme disease. A randomized, double-blind, placebo-controlled trial. Ann Intern Med. 2003;138(9):697-704.

132. Dattwyler RJ, Wormser GP, Rush TJ, Finkel MF, Schoen RT, Grunwaldt E, Franklin M, Hilton E, Bryant GL, Agger WA, Maladorno D. A comparison of two treatment regimens of ceftriaxone in late Lyme disease. Wien KlinWochenschr. 2005;117(11-12):393-7.

133. Borg R, Dotevall L, Hagberg L, Maraspin V, Lotric-Furlan S, Cimperman J, Strle F. Intravenous ceftriaxone compared with oral doxycycline for the treatment of Lyme neuroborreliosis. Scand J Infect Dis. 2005;37(6-7):449-54.

134. Ogrinc K, Logar M, Lotric-Furlan S, Cerar D, Ruzic-Sabljic E, Strle F. Doxycycline versus ceftriaxone for the treatment of patients with chronic Lyme borreliosis. Wien Klin Wochenschr. 2006;118(21-22):696-701.

135. Krüger H, Reuss K, Pulz M, Rohrbach E, Pflughaupt KW, Martin R, Mertens HG. Meningoradiculitis and encephalomyelitis due to Borrelia burgdorferi: a follow-up study of 72 patients over 27 years. J Neurol. 1989;236(6):322-8.

136. Hammers-Berggren S, Hansen K, Lebech AM, Karlsson M. Borrelia burgdorferi-specific intrathecal antibody production in neuroborreliosis: a follow-up study. Neurology. 1993;43(1):169-75.

137. Berglund J, Stjernberg L, Ornstein K, Tykesson-Joelsson K, Walter H. 5-y Follow-up study of patients with neuroborreliosis. Scand J Infect Dis. 2002;34(6):421-5.

138. Treib J, Fernandez A, Haass A, Grauer MT, Holzer G, Woessner R. Clinical and serologic follow-up in patients with neuroborreliosis. Neurology. 1998;51(5):1489-91.

139. Kohlhepp W, Oschmann P, Mertens HG. Treatment of Lyme borreliosis. Randomized comparison of doxycycline and penicillin G. J Neurol. 1989;236(8):464-9.

140. Honegr K, Hulinská D, Beran J, Dostál V, Havlasová J, Cermáková Z. Long term and repeated electron microscopy and PCR detection of Borrelia burgdorferi sensu lato after an antibiotic treatment. Cent Eur J Public Health. 2004;12(1):6-11.

141. Strle F, Preac-Mursic V, Cimperman J, Ruzic E, Maraspin V, Jereb M. Azithromycin versus doxycycline for treatment of erythema migrans: clinical and microbiological findings. Infection. 1993;21(2):83-8.

142. Strle F, Maraspin V, Lotric-Furlan S, Ruzić-Sabljić E, Cimperman J. Azithromycin and doxycycline for treatment of Borrelia culture-positive erythema migrans. Infection. 1996;24(1):64-8.

143. Dersch R, Freitag MH, Schmidt S, Sommer H, Rauer S, Meerpohl JJ. Efficacy and safety of pharmacological treatments for acute Lyme neuroborreliosis – a systematic review. Eur J Neurol. 2015;22(9):1249-59.

144. Weber K, Neubert U. Clinical features of early erythema migrans disease and related disorders. Zentralbl Bakteriol Mikrobiol Hyg A. 1986;263(1-2):209-28.

145. Ljostat U, Skogvoll E, Eikeland R, Midgard R, Skarpaas T, Berg A, Mygland A. Oral doxycycline versus intravenous ceftriaxone for European Lyme neuroborreliosis: a multicenter, non-inferiority, double-blind, randomized trial. Lancet Neurol. 2008;7(8):690-5.

146. Donta ST. Tetracycline therapy for chronic Lyme disease. Clin Infect Dis. 1997;25 Suppl 1:552-6.

147. Donta ST. Macrolide therapy of chronic Lyme Disease. Med Sci Monit. 2003;9(11):PI136-42.

148. Breier F, Khanakah G, Stanek G, Kunz G, Aberer E, Schmidt B, Tappeiner G. Isolation and polymerase chain reaction typing of Borrelia afzelii from a skin lesion in a seronegative patient with generalized ulcerating bullous lichen sclerosus et atrophicus. Br J Dermatol. 2001;144(2):387-92.

149. Nadelman RB, Pavia CS, Magnarelli LA, Wormser GP. Isolation of Borrelia burgdorferi from the blood of seven patients with Lyme disease. Am J Med. 1990;88(1):21-6.

150. Steere AC, Duray PH, Butcher EC. Spirochetal antigens and lymphoid cell surface markers in Lyme synovitis. Comparison with rheumatoid synovium and tonsillar lymphoid tissue. Arthritis Rheu. 1988;31(4):4878-95.

151. Dattwyler RJ, Volkman DJ, Luft BJ, Halperin JJ, Thomas J, Glightly MG. Seronegative Lyme disease. Dissociation of specific T- and B-lymphocyte responses to Borrelia burgdorferi. N Engl J Med. 1988;319(22):1441-6.

152. Berger BW. Treating Erythema chronicum migrans of Lyme disease. J Am Acad Dermatol. 1986;15(3):459-63.

153. Cadavid D, Auwaerter PG, Rumbaugh J, Gelderblom H. Antibiotics for the neurological complications of Lyme disease (Review). Cochrane Database Syst Rev. 2016;12:CD0069678.

Pleocytosis with Encephalitis/Myelitis

Cerebrospinal fluid findings in aquaporin-4 antibody positive neuromyelitis optica: results from 211 lumbar punctures

Jarius S, Paul F, Franciotta D, Ruprecht K, Ringelstein M, Bergamaschi R, Rommer P, Kleiter I, Stich O, Reuss R, Rauer S, Zettl UK, Wandinger KP, Melms A, Aktas O, Kristoferitsch W, Wildemann B. J Neurol Sci. 2011;306(1-2):82-90.

Pleocytosis in 50% of the CSF samples. Mostly minor case of pleocytosis, 19 cells/ul on average.

CSF pleocytosis and expansion of spinal lesions in Japanese multiple sclerosis with special reference to the new diagnostic criteria

Fukazawa T, Kikuchi S, Miyagishi R, Miyazaki Y, Fukaura H, Yabe I, Hamada T, Tashiro K, Sasaki H. J Neurol. 2005;252(7):824-9.

According to new diagnostic criteria, pleocytosis above 50/mm3 is considered a criterion for exclusion.

CSF characteristics in early-onset multiple sclerosis

Pohl D, Rostasy K, Reiber H, Hanefeld F. Neurology. 2004;63(10):1966-7.

Pleocytosis in 66% of MS cases.

Cerebrospinal fluid pleocytosis in multiple sclerosis patients with lesions showing reduced diffusion

Eisele P, Szabo K, Griebe M, Wolf ME, Hennerici MG, Gass A. Mult Scler. 2014:20(10):1391-5.

Pleocytosis (11–46 cells/ul) is obviously a very early and transient phenomenon of MS.
