# Supplementary material for: Guidelines for diagnosis and treatment in neurology – Lyme neuroborreliosis
Source: Ger Med Sci. 2020 Feb 27;18:Doc03. doi: 10.3205/000279 (PMC7174852; doi:10.3205/000279)
Supplement: Guideline report [file GMS-18-03-s-002.pdf]

Attachment 2: Guideline report

AWMF Register No. 030/071 Development Stage: S3

Guideline of the German Society of Neurology (DNG)

Neuroborreliosis

ICD-10 Codes: A69.2; A69.2+, L90.4

Guideline report

Keywords (German): Lyme-Borreliose, Lyme-Neuroborreliose, *Borrelia-burgdorferi*-Infektion, Bannwarth-Syndrom, lymphozytäre Meningoradikulitis, Fazialisparese, Polyradikulitis, Meningitis, Enzephalomyelitis, Polyneuropathie, Schildzecken-Borreliose

Keywords (English): Lyme disease, Lyme neuroborreliosis, *Borrelia burgdorferi* infection, Bannwarth's syndrome, lymphocytic meningoradiculitis, facial palsy, polyradiculitis, meningitis, encephalomyelitis, polyneuropathy, ixodid tick-borne borreliosis

1. Scope and purpose

Reasons for selecting the guideline topic

Lyme borreliosis is the most common tick-borne infectious disease in Europe. The Borrelia enter the skin during the blood sucking process of the hard-bodied tick *Ixodes ricinus*. There they are either inactivated by the innate (congenital) immune system or a local infection occurs. Disease develops in a small proportion of infected patients. Inflammation of the skin frequently occurs, typically as erythema migrans. As the disease progresses, the Borrelia can disseminate and infect various organs such as the skin, nervous system, joints and heart. The nervous system is involved in 3–15% of all patients with Lyme borreliosis. This usually manifests as meningoradiculitis. The late or chronic form of the disease rarely occurs but can lead to encephalomyelitis with an unfavourable prognosis. In very rare cases vasculitis of the arteries to the brain develops with consecutive strokes. If antibiotic treatment is not available or its onset is considerably delayed, serious neurological residuals can persist.

Guideline objective

This guideline on neuroborreliosis aims to provide:

- Recommendations for confirming a clinical diagnosis; clarifying, in particular, which clinical constellation warrants CSF testing
- Recommendations for stage-appropriate diagnostic testing: serological detection of IgG Borrelia antibodies using a 2-step ELISA/immunoblot process
- Recommendations on determining Borrelia-specific intrathecal antibody synthesis (Borrelia-specific CSF/serum antibody index)
- Meaningful use of molecular-diagnostic testing and culture tests
- Recommendations on diagnostic certainty (possible, probably, confirmed neuroborreliosis)
- Treatment of early- and late-stage neuroborreliosis
- Recommendations on monitoring treatment
- Recommendations on treating persisting atypical or non-specific symptoms after antibiotic treatment
- Prevention of Lyme borreliosis
- Recommendations on the follow-up observation of the tick bite
- Supplying information to patients (Appendix 6 in Attachment 1)
- The guideline does **not** include information on diseases caused by *Borrelia recurrentis* (relapsing fever)
- Matters pertaining to co-infections linked to tick-borne diseases are **not** within the scope of this guideline

Patient target group

- Children and adults suffering from neuroborreliosis or suspected of having neuroborreliosis
- Patients presenting to a physician for diagnosis and treatment of neuroborreliosis
- Patients presenting to a physician with neurological symptoms that indicate neuroborreliosis
- Patients whose symptoms persist after antibiotic treatment for neuroborreliosis and who need a differential diagnosis
- Patients presenting to a physician with questions about neuroborreliosis
- Patients presenting to a physician for a tick bite

Area of care

- In- and out-patient care

Target user group/target audience

- Information for physicians in private practices and clinics involved in treating neuroborreliosis (see Section 2, Composition of the guideline group: participation of interest groups)

2. Composition of the guideline group: participation of interest groups

Guideline group representation: participating occupational groups

Prof. Dr. Sebastian Rauer from Freiburg was commissioned by the German Society of Neurology (DGN) to coordinate the first draft of the guideline manuscript, to prepare consensus building, to draft the decisions of the guideline group in the context of consensus building and to prepare the guideline report. This S3 guideline is module 2 of a scheduled interdisciplinary S3 guideline on the "Diagnosis and Treatment of Lyme Borreliosis". Module 1 "Cutaneous Lyme Borreliosis" is currently being developed from an S2k into an S3 guideline.

After consensus on the key issues, the initial draft of the manuscript, authored by Prof. S. Rauer, was revised and evaluated by a DGN expert group (see below) using a modified Delphi procedure. The expert group consisted of 3 national representatives as well as a representative from Austria (Prof. E. Schmutzhardt) and Switzerland (Prof. M. Sturzenegger) respectively.

Due to the high complexity and interdisciplinary nature of the topic, neurologists were joined by other physicians, a natural scientist (Prof. Dr. rer. nat. R. Wallich) and a veterinarian (Dr. med. vet. Hendrik Wilking) in developing the guideline and participating in the consensus process. A total of 20 AWMF member societies, the Robert Koch Institute, 3 patient organisations and the German Borreliosis Society were involved in the development of the guideline.

Guideline group representation: participation of patients

Representatives from 3 patient organisations were actively involved in the consensus process:

- Action Alliance Against Tick-Borne Infections Germany (OnLyme-Aktion)
- The Borreliosis and FSME Association Germany (BFBD)
- The German Federal Association for Tick-Borne Diseases (BZK) (involvement in the S3 guideline on neuroborreliosis since August 2016)

In order to give patients room for discussion beyond the regular consensus conferences, an additional conference was held with patient representatives, the German Borreliosis Society and members of the steering group in Frankfurt am Main on 17 January 2017.

Guideline group representation: participating medical societies and organisations

Steering group
Leading:
Prof. Dr. med. Sebastian Rauer – coordinator
with collaboration from Dr. med. Rick Dersch (evidence process)
German Society of Neurology (DGN)

PD Dr. med. Stephan Kastenbauer – (deputy coordinator)
German Society of Neurology

Prof. Dr. med. Heidelore Hofmann – coordinator
German Dermatology Society (DDG)

Dr. med. Volker Fingerle
German Society for Hygiene and Microbiology (DGHM)

Prof. Dr. med. Hans-Iko Huppertz
German Society of Paediatrics and Adolescent Medicine (DGKJ) and
German Society of Paediatric Infectology (DGPI)

Prof. Dr. med. Klaus-Peter Hunfeld
The German United Society of Clinical Chemistry and Laboratory Medicine (DGKL) and INSTAND e.V.
Prof. Dr. med. Andreas Krause
German Society of Rheumatology (DGRh)

Prof. Dr. med. Bernhard Ruf
German Society of Infectious Diseases (DGI)

Advisory group of experts (appointed by the DGN guideline committee)
Prof. Dr. R. Kaiser, Clinic for Neurology, Helios Hospital Pforzheim
Prof. Dr. H. W. Kölmel, former Clinic for Neurology, Helios Hospital Erfurt
Prof. Dr. H. W. Pfister, Neurological Clinic, Ludwig Maximilians University Munich
Prof. Dr. E. Schmutzhard, University Hospital for Neurology – NICU, Medical University of Innsbruck, Austria (on behalf of the Austrian Society of Neurology)
Prof. Dr. M. Sturzenegger, University Clinic for Neurology, Inselspital, University of Bern, Switzerland (on behalf of the Swiss Neurological Society)

Consensus group (alphabetically) (the steering group is a component of the consensus group)

Prof. Dr. med. Karl Bechter
The German Association of Psychiatry, Psychotherapy and Psychosomatics (DGPPN)

PD Dr. med. Walter Berghoff
German Borreliosis Society (DBG)

Ursula Dahlem
Action Alliance Against Tick-Borne Infections Germany (OnLyme-Aktion)

Ute Fischer
Borreliosis and FSME Association Germany (BFBD)

Prof. Dr. med. Michael H. Freitag
German College of General Practitioners and Family Physicians (DEGAM)

PD Dr. med. Gudrun Gossrau
German Pain Society (DGSS)

Prof. Dr. med. Gerd Gross
Paul Ehrlich Society for Chemotherapy (PEG)

Prof. Dr. med. Rainer Müller
German Society of Oto-Rhino-Laryngology, Head and Neck Surgery (DGHNO-KHC)

Prof. Dr. med. Mathias Pauschinger
German Society of Cardiology and Cardiovascular Research (DGK)

Prof. Dr. med. Monika A. Rieger
German Society for Occupational and Environmental Medicine (DGAUM)

Prof. Dr. med. Rainer Schäfert
German Society of Psychosomatic Medicine and Medical Psychotherapy (DGPM) and the German College of Psychosomatic Medicine (DKPM)

Christel Schmedt
German Federal Association for Tick-Borne Diseases (BZK)

Prof. Dr. med. Stephan Thurau
German Ophthalmological Society (DOG)

Prof. Dr. rer. nat. Reinhard Wallich
German Society for Immunology (DGfI)

Dr. med. vet. Hendrik Wilking
Robert Koch Institute (RKI)

Moderation
Prof. Dr. med. Ina B. Kopp
AWMF Institute for Medical Knowledge Management

3. Methodological accuracy

Review, selection and evaluation of scientific evidence (evidence-based)

The systematic literature survey and evaluation was carried out by the German Cochrane Centre Freiburg (Cochrane Germany) in collaboration with Dr. Rick Dersch. The procedure was conducted in line with the **PICO** process (**P** = patient characteristics, clinical problem; **I** = intervention; **C** = comparison [compare with alternatives]; **O** = outcome [target criteria]).

Formulation of key questions

The key questions for the literature survey were formulated at an initial meeting with formal consensus by the consensus group. They were developed with respect to patients, interventions, comparative interventions (comparatives) and patient-relevant endpoints (outcomes) in accordance with the PICO process. The meeting was chaired by an independent moderator from the AWMF in Frankfurt am Main on 11 February 2014.

*a) Definition of neuroborreliosis (**P**ICO):*

In infectiology, the microbiological detection of pathogens is considered the "gold standard" for defining an infectious disease. Since detecting the pathogen in CSF is not sensitive enough with respect to neuroborreliosis (10–30% sensitivity), diagnostic criteria have been agreed on which

define the disease based on a combination of typical clinical symptoms, CSF findings and Borrelia serology and based on recommendations by earlier reviews and existing guidelines (Halperin et al. 2007; Kaiser 1998; Mygland et al. 2010; Rauer et al. 2012; Stanek et al. 2011); this definition differentiates between a "possible", "probable" and "confirmed" case of neuroborreliosis (Section 3.11 of the guideline text).

Discussion on the above procedure:

- "Seronegative cases" (controversial) are not taken into account in clinical definitions:
  Decision: These should be taken into account in the descriptive review and discussed as part of the recommendations under the aspect of their transferability to extended patient groups.
- Serological tests are not uniformly validated:
  Decision: the type of serological test should be provided in study extracts (validity)
- "NB without CSF pleocytosis" against the backdrop of a possible biomarker (e.g. cytokines like CXCL13): Decision: take into account in descriptive review

Strong consensus: 13/13

*b) Intervention, comparison with alternative (P**IC**O)*:

- Antibiotic treatment vs. placebo
- Comparison of antibiotic treatments in terms of: classes/substances, application form, dose, duration, drug level
- The following antibiotics should be examined/compared: amoxicillin, azithromycin, cefotaxime, ceftriaxone, cefuroxime, clarithromycin, doxycycline, penicillin, metronidazole, minocycline, bactrim, erythromycin, quinolone, hydroxychloroquine
- Non-steroidal antiphlogistic drugs compared to placebo or no non-steroidal antiphlogistic drugs
- Steroids compared to placebo or no steroids
- Take phytotherapeutic drugs into consideration in descriptive review (frankincense, Curcumin, Artemisia annua, Samento, Banderol)

Strong consensus: 13/13

*c) Patient-relevant endpoints (outcomes) (PIC**O**)*:

- Neurological state (general)
- Neurological state (specific)
  - Facial palsy
  - Hearing disorder
  - Visual disorder
  - Paresis of the extremities
  - Spinal symptoms
  - Dysesthesia/paraesthesia
  - Dizziness
- Scales
  - Quality of life (SF36)
  - Cognition (CVLT, TMT)
  - Depression (BDI)
  - Pain (SF36)

- Fatigue (SF36)
- Sleep disorders
- Ability to work/earning capacity %
- Degree of disability

Endpoints should generally be measured using validated scales (the above scales are considered examples)
Strong consensus: 13/13

Utilisation of existing guidelines on the topic (Dersch et al. 2015b)

To find relevant guidelines, a literature survey was carried out in the electronic database MEDLINE (via Ovid) and in the databases of four guideline networks (National Guideline Clearinghouse (www.guideline.gov), International Guideline Library of the Guidelines International Network (www.g-i-n.net/library/interational-guidelines-library), the National Institute for Health and Care Excellence (NICE, www.nice.org.uk/guidance/published?type=guidelines), and the Association of Scientific Medical Societies (AWMF, www.awmf.org/leitlinien/leitlinien-suche.html). All guidelines published in these databases between 1999 and 2014 and published in German or English have been included (see Appendix for search strategy).

First a literature survey was carried out of the relevant guidelines. A total of 177 guidelines were found, of which only 8 met the inclusion criteria. Six guidelines were issued by scientific societies; the remaining two guidelines were issued by self-help organisations and patient associations.

The "Appraisal of Guidelines for Research and Evaluation II" questionnaire (AGREE II) was used to assess and rate the methodological quality of the guidelines (Brouwers et al. 2010). Assessments were carried out in a total of 6 domains based on predefined assessment criteria (scope of application, participation of interest groups, stringency of guideline development, clarity of design, applicability and editorial independence). These domains were used to calculate an overall percentage rating (%). A guideline receiving an overall rating of <50% is considered to be of low methodological quality (Bouwmeester et al. 2009; Haran et al. 2014).

The domain "Methodological precision of guideline development" is also relevant, as methodological aspects of evidence-based research (systematic literature survey, selection of literature) play a particularly important role in this area. A guideline receiving a rating of <50% in this domain was also considered to be of low methodological quality.

A total of 177 entries were screened in the various databases. After excluding irrelevant entries, 8 guidelines remained. Their full texts were evaluated using the AGREE II tool.

An assessment of the non-excluded guidelines was independently carried out by two experts; their evaluations are presented in Table 1.

Three of these guidelines had an overall rating ≥50%. None of the guidelines included under the aspect "methodological precision" had a value ≥50%. Because the quality of the included guidelines was questionable at best, recommendations from these guidelines were not adopted without further review and our own literature survey was conducted.

Appendix 1: MEDLINE (OVID) search strategy for guidelines

1. exp Lyme Disease/
2. lyme*.mp.
3. exp Borrelia burgdorferi Group/
4. borrel*.mp.
5. 1 or 2 or 3 or 4
6. exp practice guideline/
7. Health Planning Guidelines/
8. guideline*.ti.
9. (practice adj3 parameter*).ti,ab.
10. clinical protocols/
11. guidance.ti,ab.
12. care pathway*.ti,ab.
13. critical pathway/
14. (clinical adj3 pathway*).ti,ab.
15. algorithms/
16. consensus development conference.pt.
17. consensus development conference nih.pt.
18. 6 or 7 or 8 or 9 or 10 or 11 or 12 or 13 or 14 or 15 or 16 or 17
19. 5 and 18

1. Search strategy on guideline websites:
2. All search hits with “lyme” or “borrel*” were screened.

Table 1: Evaluation of guidelines according to AGREE II

Guideline	Scope	Participation of interest groups	Stringency of the guideline development	Clarity of design	Applica-bility	Editorial indepen-dence	Overall rating
BIA 2010	0.53	0.11	0.09	0.81	0.08	0	0.33
DBG 2010	0.33	0.28	0.10	0.53	0	0.17	0.25
DGN 2012	0.28	0.11	0.17	0.64	0.13	0.17	0.33
EFNS 2010	0.47	0.17	0.23	0.81	0.04	0.08	0.58
DGPI 1999	0.33	0.14	0.10	0.69	0.06	0	0.25
IDSA 2006	0.61	0.5	0.22	0.86	0.02	0.17	0.5
ILADS 2004	0.56	0.36	0.18	0.22	0.04	0.13	0.42
AAN 2007	0.5	0.31	0.37	0.64	0	0.54	0.5

Systematic literature survey (Dersch et al. 2015a)

A systematic literature survey was conducted in order to assess the pharmacological processes for treating neuroborreliosis. This was followed by an evaluation and summary of existing literature.

The search strategy and methodology of this systematic review were reviewed and published in advance as part of a peer review process (Dersch et al. 2014). The literature was to be summarised and evaluated separately for adults and children.

Diagnoses had to be made on the basis of internationally agreed case definitions (see above). Studies had to report data on drug therapy for patients with neuroborreliosis and include a control group.

A search for existing literature was conducted in three literature databases: MEDLINE (via Ovid), EMBASE (via Scopus) and the Cochrane Central Register of Controlled Trials.

The search strategy for the respective literature databases is listed in Appendix 2.

Appendix 2. Medline (OVID) Search Strategy

1. exp Lyme Disease/
2. lyme*.mp.
3. neuroborreliosis.mp.
4. borreli*.mp.
5. exp Borrelia/
6. (erythem* adj2 migran*).mp.
7. or/1-6
8. exp Brain/
9. brain*.mp.
10. mening*.mp.
11. spinal*.mp.
12. exp Nervous System Diseases/
13. encephal*.mp.
14. radiculi*.mp.
15. radiculo*.mp.
16. Facial Paralysis/
17. facial pal*.mp.
18. facial par*.mp.
19. Myelitis/
20. myel*.mp.
21. (nervous system adj5 dis*).mp.
22. neur*.mp.
23. polyneur*.mp.
24. polyradicul*.mp.
25. mononeur*.mp.
26. (nerve adj5 damage*).mp.
27. (nerve adj5 involvement).mp.
28. bannwarth*.mp.
29. vasculitis/
30. exp vasculitis, central nervous system/
31. vasculiti*.mp.
32. cranial nerve*.mp.
33. or/8-32
34. 7 and 33

SCOPUS Search Strategy

1. TITLE-ABS-KEY(lyme*) OR TITLE-ABS-KEY(neuroborreliosis) OR TITLE-ABS-KEY(borreli*) OR TITLE-ABS-KEY(erythema migrans)
2. TITLE-ABS-KEY(brain*) OR TITLE-ABS-KEY(mening*) OR TITLE-ABS-KEY(spinal*) OR TITLE-ABS-KEY(encephal*) OR TITLE-ABS-KEY(radiculi*) OR TITLE-ABS-KEY(radiculo*) OR TITLE-ABS-KEY(facial pal*) OR TITLE-ABS-KEY(facial par*) OR TITLE-ABS-KEY(myel*) OR TITLE-ABS-KEY(nervous system dis*) OR TITLE-ABS-KEY(neur*) OR TITLE-ABS-KEY(polyneur*) OR TITLE-ABS-KEY(polyradicul*) OR TITLE-ABS-KEY(mononeur*) OR TITLE-ABS-KEY(nerve AND damage*) OR TITLE-ABS-KEY(nerve AND involve*) OR TITLE-ABS-KEY(bannwarth*) OR TITLE-ABS-KEY(vasculiti*) OR TITLE-ABS-KEY(cranial nerve*)
3. 1 AND 2

Cochrane CENTRAL Search Strategy

1. MeSH descriptor: [Borrelia] explode all trees
2. MeSH descriptor: [Lyme Disease] explode all trees
3. *borreli*
4. erythem* near/2 migran*
5. lyme*
6. 1 OR 2 OR 3 OR 4 OR 5

Treating neuroborreliosis in adults (Dersch et al. 2015a)

Selecting evidence

A survey of the literature found a total of 5,779 entries after duplicates were removed. Irrelevant entries were eliminated by screening the titles and abstracts of each entry. This left 119 texts that were examined in their entirety. A further 86 entries were excluded on the basis of being irrelevant. Of the remaining 33 studies, 17 studies had only one treatment arm so no data could be extracted for statistical comparisons. A total of 16 studies had two or more treatment arms and thus data could be extracted for a meta-analysis. Of these 16 trials, eight were randomised controlled trials (RCTs). Figure 1 is a flow chart showing the studies that were included (PRISMA statement). The characteristics of the RCTs are shown in Table 2.

Figure 1: Flow Chart of Included Studies

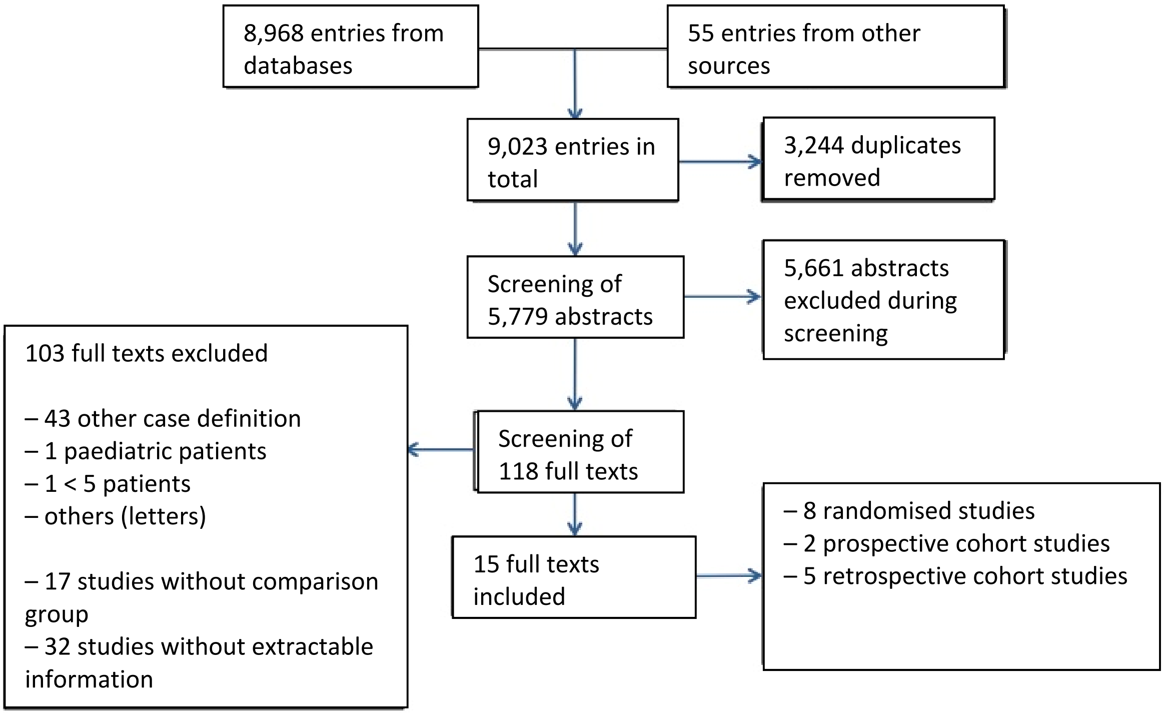

Table 2: Study characteristics

Study	Participants	Case definition	Interventions	Treatment duration
Ljostad 2008 [17]	102	Definite (n=71)<br>Possible (n=31)	Ceftriaxone 2g (n=48)<br>vs.<br>Doxycycline 200 mg (n=54)	14 days
Oksi 1998 [21]	60	Possible	Cefixime 200 mg + probenecid 500 mg (n=30)<br>vs.<br>Amoxicillin 500 mg + probenecid 500mg (n=30)	100 days
Karlsson 1994 [15]	54	Probable	Penicillin G 12g/day (n=23)<br>vs.<br>Doxycycline 200 mg/day (n=31)	14 days
Pfister 1991 [24]	33	Probable	Ceftriaxone 2g/day (n=17)<br>vs.<br>Cefotaxime 8g/day (n=16)	10 days
Hassler 1990 [12]	135	Possible	Penicillin G 20 MioU/day (n=44)<br>vs.<br>Cefotaxime 6g/day (n=49)	10 days
Kohlhepp 1989 [16]	75	Possible	Penicillin G 20 MioU/day (n=36)<br>vs.<br>Doxycycline 100mg/day (200mg on day 1) (n=39)	10 days
Pfister 1989 [23]	21	Possible	Cefotaxime 6g/day (n=11)<br>vs.<br>Penicillin G 20 MioU/day (n=10)	10 days
Pfister 1988 [22]	21	Possible	Penicillin/doxycycline + methylprednisolone 60mg/day (n=11) vs. Penicillin/doxycycline + placebo (n=10)	7 days

Evaluation of the evidence

The quality of the individual RCTs was investigated and evaluated using the risk-of-bias tool from the Cochrane Collaboration (www.handbook.cochrane.org). The quality of the non-randomised studies (cohort studies) was measured using the ACROBAT-NRSI tool of the Cochrane Collaboration (www.riskofbias.info). The entire body of evidence was evaluated using the GRADE methodology (Grading of Recommendations Assessment, Development and Evaluation) (Balshem et al. 2011). The data extraction and risk of bias were carried out by two independent experts. For a meta-analysis of the existing studies, pooled effect estimates were calculated for the treatment effects using a "fixed effects model" based on the Mantel-Haenszel method. There were no studies on the treatment of neuroborreliosis in which antibiotic treatment was compared to a placebo.

Creation of evidence tables

The GRADE methodology was used by two experts to independently evaluate the quality of the evidence with regard to the individual comparisons. The evaluation of the individual comparisons is summarised in evidence tables (Tables 3–5).

Table 3: GRADE evidence table comparing beta lactam antibiotics with doxycycline in the treatment of adults with neuroborreliosis

Quality assessment							Number of patients		Effect estimate	Quality
**Number of studies**	**Study design**	**Risk of bias**	**Inconsistency**	**Indirectness**	**Imprecision**	**Other**	**Beta lactam**	**Doxycycline**	**Relatives risk (95% CI)**	
Residual neurological symptoms (after 4–12 months)										
3	RCTs	severe[1]	low	low	severe[2]	none	59/105 (56.2%)	53/124 (42.7%)	**RR 1.27** (0.98–1.63)	⊕⊕◯◯ LOW
Residual neurological symptoms (after 12 or more months)										
3	RCTs	severe[1]	low	low	severe[3]	none	33/98 (33.7%)	37/113 (32.7%)	**RR 0.98** (0.68–1.42)	⊕⊕◯◯ LOW
Side effects										
3	RCTs	severe[1]	low	low	severe[3]	none	24/80 (30.0%)	30/87 (34.5%)	**RR 0.82** (0.54–1.25)	⊕⊕◯◯ LOW

1. Two unblinded studies, concerns regarding the allocation and selective reporting
2. Small groups, the ‘optimal information size’ was not reached, wide confidence interval
3. The ‘optimal information size’ was not reached, wide confidence interval

Table 4: GRADE evidence table comparing penicillin with cefotaxime in the treatment of adults with neuroborreliosis

Quality Assessment							Number of patients		Effect estimate	Quality
**Number of studies**	**Study design**	**Risk of bias**	**Inconsistency**	**Indirectness**	**Imprecision**	**Other**	**Penicillin**	**Cefotaxime**	**Relative risk (95% CI)**	
Residual neurological symptoms (after 4 or more months)										
2	RCTs	severe[1]	low	low	severe[2]	none	26/54 (48.1%)	16/60 (26.7%)	**RR 1.81** (1.1–2.97)	⊕⊕◯◯ LOW
Side effects										
2	RCTs	severe[1]	none[3]	low	severe[2]	none	20/79 (25.3%)	37/80 (46.3%)	**RR 0.54** (0.35–0.83)	⊕⊕◯◯ LOW

1. No blinding, concerns regarding the allocation and selective reporting
2. Small group size, the ‘optimal information size’ was not reached
3. One study reported no side effects in both intervention arms, effect estimate therefore only stems from one study. Inconsistency can therefore not be ruled out.

Table 5: GRADE evidence table comparing combination therapy with antibiotic monotherapy in the treatment of adults with neuroborreliosis

Quality assessment							Number of patients		Effect estimate	Quality
**Number of studies**	**Study design**	**Risk of bias**	**Inconsistency**	**Indirectness**	**Imprecision**	**Other**	**Combination**	**Monotherapy**	**Relative risk (95% CI)**	
Residual neurological symptoms										
2	Observational studies	very severe[1]	low	none	severe[3]	none	2/8 (25.0%)	4/10 (40.0%)	No meta-analysis	⊕◯◯◯ VERY LOW

1. Critical risk of bias (interventions not clearly described, baseline confounding, no blinding), meta-analysis is therefore not justified
2. Low heterogeneity (various interventions, various treatment durations), relevance for effect estimate unclear
3. The ‘optimal information size’ was not reached, wide confidence intervals

Treating neuroborreliosis in children (Dersch et al. 2016a)

The body of evidence on treating neuroborreliosis in childhood was also compiled from clinical studies and evaluated in a systematic review. The methodology is based on the systematic review mentioned above on treating neuroborreliosis in adulthood (Dersch et al. 2014).

Systematic literature survey

Inclusion and exclusion criteria were defined and published in advance (Dersch et al. 2014). The patients in the individual studies had to be <18 years old. The diagnosis had to be transparent in the individual studies. There are no established case definitions for diagnosing neuroborreliosis in childhood. Therefore, the diagnostic criteria of neuroborreliosis in adulthood were used as inclusion criteria for the individual studies in the systematic review (cf. guideline Section 3.11). For a meta-analysis of the existing studies, pooled effect estimates were calculated for the treatment effects using a "fixed effects model" based the Mantel-Haenszel method.

A survey of the literature identified a total of 5,779 entries after duplicates were removed. Irrelevant entries were eliminated by screening the titles and abstracts of each entry. This left 44 texts that were examined in their entirety. A further 38 entries were excluded on the basis of being irrelevant. A total of six studies met the inclusion criteria including two RCTs, a prospective cohort study and three retrospective cohort studies. Figure 2 is a flow chart showing the included studies (PRISMA statement). The study characteristics of the included studies are shown in Table 6.

Figure 2: Included studies (PRISMA statement)

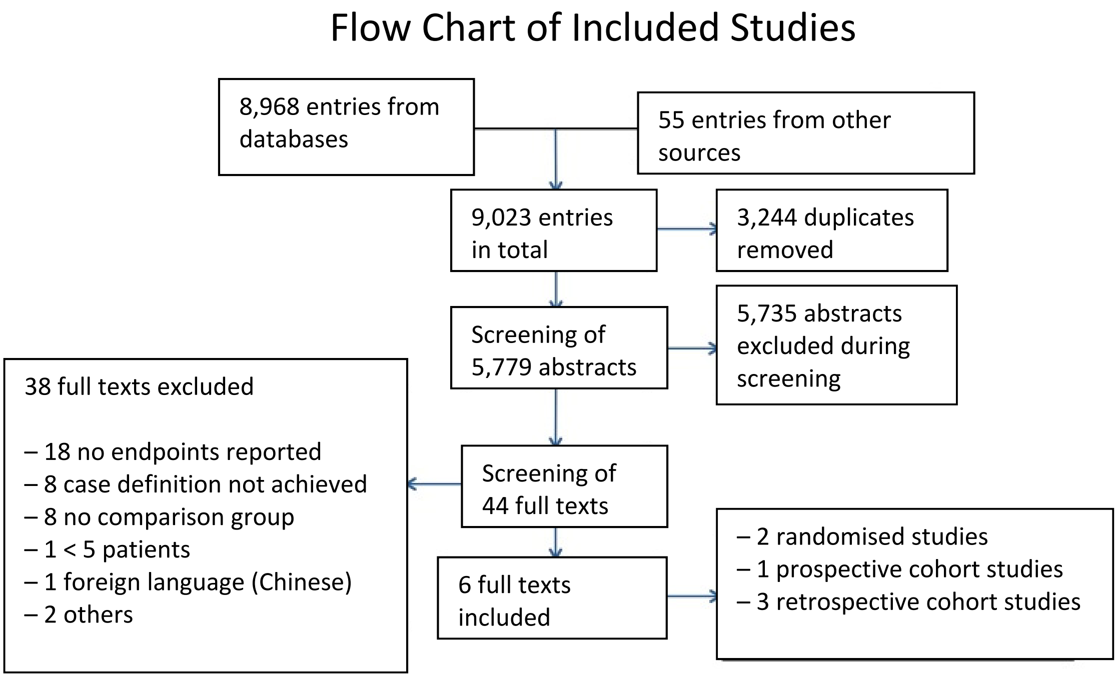

Selecting evidence

The selected studies and the results of the evidence analysis are shown in Table 6 (see below) and in Section 5.5 of the guideline text.

Table 6: Study characteristics of the included studies

Study	Participants	Case definition	Intervention	Treatment duration
**RCTs**				
Millner 1995 [18]	41	Possible	Penicillin G 300000–375000 IU/kg<br>Ceftriaxone 100mg/kg<br>Group size not reported	14 days
Müllegger 1991 [19]	23	Possible	Penicillin G 400000–500000 IU/kg (n=11)<br>Ceftriaxone 75–93 mg/kg (n=12)	14 days
**Prospective cohort studies**				
Jörbeck 1987 [13]	9	Possible	Penicillin G 150 mg/kg (n=8)<br>Cefuroxime 4.5g (n=1)	10–19 days
**Retrospective cohort studies**				
Thorstrand 2002 [28]	203	Probable	Ceftriaxone 100mg/kg, maximum 2g (n=109)<br>Penicillin 100mg/kg (n=53)<br>Doxycycline 4mg/kg, maximum 200mg (n=22)<br>Cefotaxime 100mg/kg (n=19)	10 days
Bingham 1995 [2]	19	Possible	Ceftriaxone, amoxicillin, erythromycin, penicillin, doxycycline, steroids, acyclovir or no treatment. Doses not reported.	14–30 days
Skowronek-Bala 2008 [26]	9	Possible	Ceftazidime + doxycycline (n=5), amoxicillin + doxy-cycline (n=1), ceftazidime + amoxicillin (n=1), doxycycline (n=1), ceftazidime (n=1)	3–6 weeks

Assessment of the evidence

The quality of the individual studies was examined and evaluated using the risk of bias tool from the Cochrane Collaboration (www.handbook.cochrane.org). The entire body of evidence was analysed using the GRADE methodology (Grading of Recommendations Assessment, Development and Evaluation) (Balshem et al. 2011). Two experts independently extracted the data and assessed the risk of bias.

Creation of evidence tables

The assessment of the individual comparisons is summarised in evidence tables (Table 7–9).

Table 7: GRADE evidence table comparing beta-lactam with doxycycline in the treatment of children with neuroborreliosis

Quality assessment						Number of patients		Effect estimate	Quality
**Number of studies**	**Study design**	**Risk of bias**	**Inconsistency**	**Indirectness**	**Imprecision**	**Beta lactam**	**Doxycycline**	**Relative risk (95% CI)**	
Residual neurological symptoms at the last reported point in time									
3	Observational studies	very severe[1]	low	severe[2]	severe[3]	15/195 (7.7%)	3/25 (12.0%)	No meta-analysis	⊕◯◯◯ VERY LOW

1. Baseline confounding, selected patients, no blinding, unclear description of interventions, meta-analysis therefore not justified
2. Heterogeneous interventions, interventions not clearly described
3. The ‘optimal information size’ was not reached, wide confidence intervals

Table 8: GRADE evidence table comparing penicillin with ceftriaxone in the treatment of children with neuroborreliosis

**Quality assessment**						**Number of patients**		**Effect estimate**	**Quality**
**Number of studies**	**Study design**	**Risk of bias**	**Inconsistency**	**Indirectness**	**Imprecision**	**Penicillin**	**Ceftriaxone**	**Relative risk (95% CI)**	
Residual neurological symptoms at the last reported point in time									
1	RCTs	severe[4]	low	low	very severe[5]	0/11 (0,0%)	0/12 (0,0%)	Not determinable	⊕◯◯◯ VERY LOW
Residual neurological symptoms at the last reported point in time									
2	Observational studies	very severe[1]	low	severe[2]	severe[3]	2/55 (3,6%)	4/28 (14,3%)	No meta-analysis	⊕◯◯◯ VERY LOW

1. Baseline confounding, selected patients, no blinding, insufficient description of interventions
2. Heterogeneous interventions, interventions not clearly described
3. Small group size, the ‘optimal information size’ was not reached
4. No blinding, randomisation and allocation not clearly described, selective reporting cannot be ruled out
5. Very small group size, the ‘optimal information size’ was not reached

Table 9: GRADE evidence table comparing combination therapy with antibiotic monotherapy in the treatment of with neuroborreliosis

Quality assessment						Number of patients		Effect estimate	Quality
**Number of studies**	**Study design**	**Risk of bias**	**Inconsistency**	**Indirectness**	**Imprecision**	**Combination**	**Monotherapy**	**Relative risk (95% CI)**	
Residual neurological symptoms at the last reported point in time									
1	Observational studies	very severe[1]	low	low[3]	low[2]	1/7 (14.3%)	2/2 (100.0%)	**RR 4,44** (0.96–20.50)	⊕◯◯◯ VERY LOW

1. Critical risk of bias, baseline confounding, selected patients, absence of blinding, imprecise description of interventions
2. Small group size, the ‘optimal information size’ was not reached
3. Heterogenous interventions

Prognosis of neuroborreliosis (Dersch et al. 2016b)

A systematic review investigated the prevalence and spectrum of residual symptoms following neuroborreliosis with the aim of making evidence-based statements on the prognosis and progression of neuroborreliosis (Dersch et al. 2016b). Residual symptoms refer here to neurological symptoms that were present before therapy as disease symptoms and which remain after therapy.

Systematic literature survey
A literature survey was conducted in three electronic databases (MEDLINE, EMBASE and Central) using a previously published, broad-based search strategy (Dersch et al. 2014). The search strategy was the same as in the literature surveys described above for the treatment of neuroborreliosis, however it also contained studies that did not include a control group (studies with only one treatment arm). The diagnosis had to be made on the basis of internationally established case definitions (cf. Section 3.11. of the guideline).

Selecting evidence
A survey of the literature found a total of 5,779 entries after duplicates were removed. Irrelevant entries were eliminated by screening the titles and abstracts of each entry. This left 118 texts that were then examined in their entirety. A further 74 entries were excluded on the basis of being irrelevant. Of the remaining 44 studies, 38 studies contained data on the prevalence of residual symptoms and 31 studies contained data on the spectrum of the residual symptoms. A flow chart showing the included studies (PRISMA statement) is depicted in Figure 3.

Data on residual symptoms were extracted from the individual studies as reported by the original authors. The data on the spectrum of residual symptoms were combined into categories to allow a meaningful comparison (e.g. data on “facial nerve palsy” and “aducens nerve palsy” were placed into the category “cranial nerve palsy”). It was also recorded how the diagnosis of neuroborreliosis was made in the individual studies. Thus, the patient cohorts of the individual studies were categorised based on the case definitions of neuroborreliosis.

Figure 3: Included studies on residual symptoms following neuroborreliosis

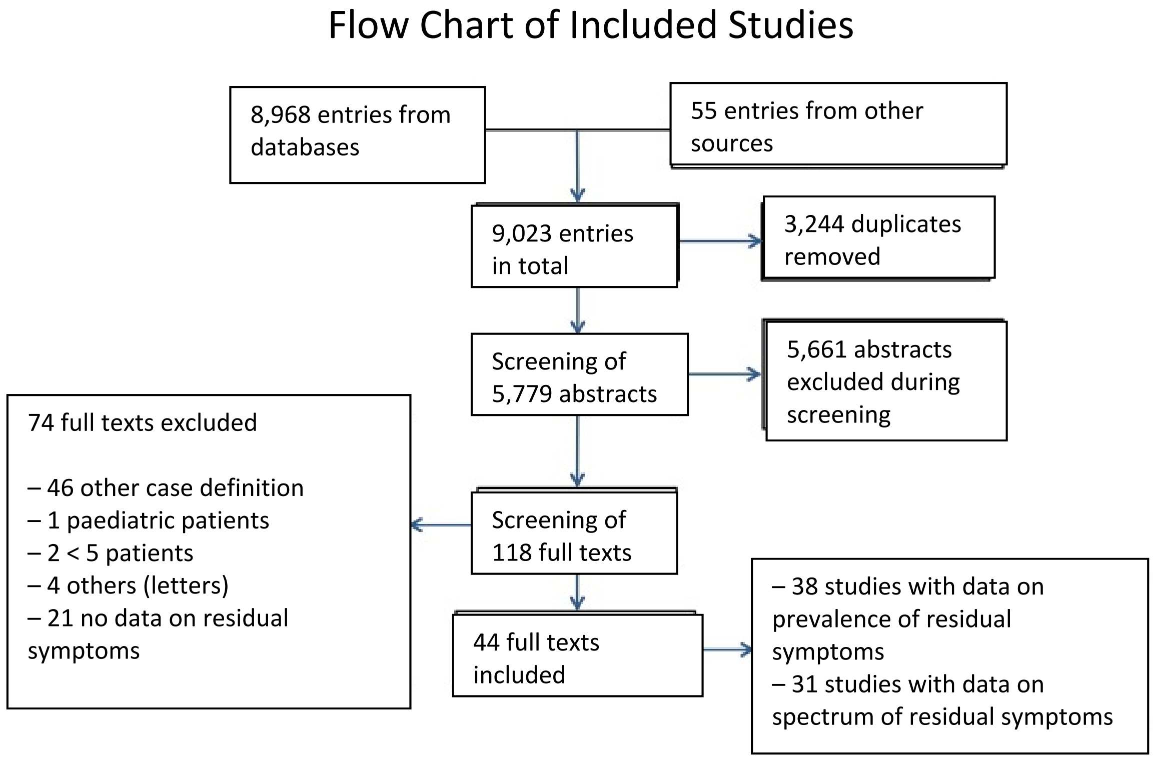

Prevalence of residual symptoms

The prevalence of residual symptoms across all available studies was compiled in a meta-analysis. Since the heterogeneity of study populations was assumed to be high, a “random effects model” was used to calculate the meta-analysis for the prevalence of residual symptoms.

The meta-analysis showed a prevalence of residual symptoms after treatment of 28% (95% CI 23–34%) across all studies. The prevalence of residual symptoms differed depending on the case definition used. In studies that included patients without a diagnosis confirmed by CSF testing (“possible neuroborreliosis”), residual symptoms were statistically significantly more frequent than in studies that included patients with a neuroborreliosis diagnosis confirmed by CSF testing (“probable” and “confirmed” neuroborreliosis) (31% vs. 24%, p=0.0048).

Spectrum of the residual symptoms

The spectrum of residual symptoms was reported for a total of 687 patients in studies where the diagnosis was confirmed by CSF testing and for 624 patients in studies without confirmation by CSF testing. The results of the review are presented in Section 4.1. of the guideline.

Formulation of the recommendations and structured consensus building

Formal consensus building: process and implementation

An initial draft of the guideline was developed by Prof. S. Rauer following consensus on the key issues and based on the results of the systematic literature survey. The draft was agreed on in the expert group using a modified Delphi procedure and subsequently brought to a vote in the consensus group using the nominal group technique. Four consensus conferences were held that were independently moderated by the AWMF.

The nominal group technique contained the following steps:

- Presentation of the statements/recommendations requiring consensus
- Opportunity to pose questions to the author and moderator
- Silent note: which recommendation/recommendation grading do you not agree with?
- Formulation of alternatives, if necessary amendments
- Recording of the statements in a single round robin and
- Summary of the comments by the moderator
- Pre-vote on every recommendation and all alternatives, determination of need for discussion
- Debate/discussion, development of suggested solutions
- Final vote
- Repetition of the steps for every recommendation leading to a determination of the strength of the consensus ("consensus":>75 % agreement, "strong consensus": >95% agreement in relation to the number of participants with voting rights)
- Representation of special votes was possible (also for consensus/strong consensus). Special votes were admitted upon request when grounds were specified.
- The minutes of the meeting are filed in the guideline administration office (Prof. Dr. S. Rauer).

Table 10: Participants and voting authorisation at the consensus conference (location: Frankfurt am Main)

Name (FG/Organisation)	Authorised to vote	11/2/2014	7/12/2015	9/3/2016	13/6/2016	9/2/2017
Kopp AWMF (moderator)	No	+	–*[1]	+	+	+
Rauer DGN	No	+	+	+	+	+
Dersch Cochrane/DGN	No	+	+	+	+	+
Kastenbauer DGN	Yes	–	+	+	+	+
Hofmann DDG	Yes	+	+*[3]	+*[3]	+	+*[3]
Fingerle DGHM	Yes	+	–	+	+	+
Huppertz DFKJ/DGPI	Yes	–	+	+	–	–
Hunfeld DGKL, INSTAND	Yes	+	+	+	+	+
Krause DGRh	Yes	–	–	+	–	–
Ruf DGI	Yes	–	–	+	–	+
Bechter DGPPN	Yes	–	+	+	–	–
Berghoff DBG	Yes	–*[2]	+*[5]	+	+	+
Dahlem OnLyme Akt.	Yes	+	+*[4]	+	+	+
Fischer BFBD	Yes	+	+	+	+	+
Freitag DEGAM	Yes	+	+	+	–	+
Goßrau DGSS	Yes	+	+	+	+	–
Groß PEG	Yes	–	–	+	+	–
Müller DGHNOKHC	Yes	+	–	–	+	+
Pauschinger DGK	Yes	–	–	–	–	+
Rieger DEGAUM	Yes	–	–	–	–	–
Schäfert DGPM/DKPM	Yes	–	+	–	+	+*[6]
Schmedt BZK	Yes	–	–	–	–	+
Thurau DOG	Yes	–	–	+	+	–
Wallich DGI	Yes	+	+	–	+	–
Wilking RKI	Yes	–	+	–	–	–

+ = Present
– = Absent
*[1] Represented by: PD Dr. Helmut Sitter, Marburg
*[2] Mandate holder for the DBG at the time: Dr. Kurt Müller, present
*[3] Gabriel Torbahn, Cochrane/DDG advisory role, also present
*[4] Frank Neubert, OnLyme-Aktion, also present as a guest
*[5] Dr. Ortwin Zais, DBG, also present as a guest
*[6] PD Dr. Jonas Tesarz, also present, mandate holder for the DGPM/DKPM starting 1/4/2017

Consideration of benefits, side effects, relevant outcomes

The systematic review found that there is no reliable data on placebo-controlled treatment (Dersch et al. 2015a). At the same time, there are analysable studies comparing the efficacy and side effects of different classes of antibiotics (Dersch et al. 2015a). These are described in Section 5 of the guideline. The relevant studies are summarised in Appendixes 3, 4 and 5 of the guideline (Attachment 1). Appendix 8 of the guideline (Attachment 1) presents an evaluation of the evidence of these studies using the GRADE methodology.

Formulation of the recommendations and the grading of evidence and/or recommendations

In infectiology the microbiological detection of pathogens by culture, microscopy or PCR is the diagnostic gold standard. Accepted case definitions are used in the diagnosis of neuroborreliosis (cf. Section 3.11. of the guideline) since there is no reliable gold standard because pathogen detection in cerebrospinal fluid has a very low sensitivity rate (10–30%). Thus, for methodological reasons, controlled studies on diagnostic testing procedures can only be conducted to a very limited degree.

Evidence grading is provided in the background for all **treatment recommendations** based on the systematic reviews (Dersch et al. 2015a; Dersch et al. 2014; Dersch et al. 2016a). The presentation of evidence is based on the classification of the British guideline NICE-SCIE.

<u>Evidence grading: studies on therapy interventions</u>

Ia Evidence from a meta-analysis of at least three randomised controlled trials (RCTs)

Ib Evidence from at least one randomised controlled trial or a meta-analysis of fewer than three RCTs

IIa Evidence from at least one methodologically sound controlled study without randomisation

IIb Evidence from at least one methodologically sound, quasi-experimental descriptive study

III Evidence from a methodologically sound, non-experimental observational study, such as comparative studies, correlational studies and case studies

IV Evidence from reports by expert committees or expert opinions and/or the clinical experience of recognised authorities

<u>Uniform formulations are used in order to standardise the guideline recommendations. The following grading applies:</u>

Strong recommendation: "shall" ↑↑

Recommendation: "should" ↑

Open recommendation: "can be considered" ↔

Recommendation against an intervention: "should not" ↓

Strong recommendations against an intervention: "shall not" ↓↓

The grading of the recommendations was determined within the framework of formal consensus conferences. In addition to the quality of the underlying evidence, the following criteria were explicitly taken into account:

- Consistency in the study results, directness of evidence, precision of the effect estimates (see GRADE profile)
- Clinical relevance of the endpoints (outcomes) and effect sizes
- Risk-benefit ratio
- Legal considerations (approval status)
- Patient preferences
- Feasibility in actual medical care situation

On the basis of the above-mentioned consensus aspects, a higher or lower recommendation grading was issued in individual cases despite the level of evidence.

Approval by the boards of the publishing medical societies and organisations

The boards of the following medical societies and organisations have approved the guideline:

German Society of Neurology (DGN)
German Dermatology Society (DDG)
German College of General Practitioners and Family Physicians (DEGAM)
German Society for Occupational and Environmental Medicine (DGAUM)
German Society of Oto-Rhino-Laryngology, Head and Neck Surgery (DGHNOKHC)
German Society for Hygiene and Microbiology (DGHM)
German Society for Immunology (DGfI)
German Society of Infectious Diseases (DGI)
German Society of Cardiology and Cardiovascular Research (DGK)
German Society of Paediatrics and Adolescent Medicine (DGKJ)
The German United Society of Clinical Chemistry and Laboratory Medicine (DGKL) and INSTAND e.V.
German Society of Paediatric Infectology (DGPI)
The German Association of Psychiatry, Psychotherapy and Psychosomatics (DGPPN)
German Society of Psychosomatic Medicine and Medical Psychotherapy (DGPM) and the German College of Psychosomatic Medicine (DKPM)
German Society of Rheumatology (DGRh)
German Pain Society (DGSS)
German Ophthalmological Society (DOG)
Paul Ehrlich Society for Chemotherapy (PEG)
Robert Koch Institute

The following organisations have not approved the guideline:
German Borreliosis Society (DBG)
Action Alliance Against Tick-Borne Infections Germany (OnLyme-Aktion)
Borreliosis and FSME Association Germany (BFBD)
German Federal Association for Tick-Borne Diseases (BZK)

4. Editorial independence

Financing of the guideline

The main costs for the preparation of this guideline were generated from the one-year work of Dr. Rick Dersch at the German Cochrane Centre in Freiburg from 1 March 2014 to 28 February 2015. During this time Dr. Dersch was directly employed as a research associate at the Cochrane Centre Freiburg and was subordinate only to his superiors at the Cochrane Centre and bound by their instructions. His post was financed through external funding (approx. € 60,000) which was available for scientific purposes from the Department of Neurology at the University Medical Centre Freiburg. The five consensus conferences (room rental, catering, moderation fee) were financed by the DGN (€ 10,000). Travel costs to the consensus conferences were borne by the participants or their organisations. The drafting of the manuscript, work on the manuscript and participation in the consensus conferences were not remunerated. There was no financial contribution over and above the financing mentioned here.

Stating and countering potential conflicts of interest

Explanation and review of interests

The potential conflicts of interest were documented in a structured AWMF form by all persons working on the guideline (members of the steering committee, expert group, consensus group). The potential conflicts of interest were assessed by a panel of expert reviewers appointed by the DGN who worked anonymously, pursued the highest degree of objectivity, were committed to confidentiality and had declared their own interests with respect to the DGN. This evaluation is summarised in a table in an appendix to this report (Attachment 3).

Statement by the DGN experts – panels on assessing conflicts of interest:

The fact that the guideline coordinator Prof. Dr. S. Rauer is co-founder and co-owner of ravo Diagnostika GmbH Freiburg was seen from the start as a conflict of interest. The company develops, produces and markets serological tests for determining Borrelia-specific antibodies as part of routine diagnostic testing. For this reason, S. Rauer was generally not entitled to vote as part of the consensus process. The DGN's vote was cast by PD Dr Stephan Kastenbauer, who was appointed deputy coordinator for this task by the DGN.

Prof. A. Krause, member of the steering committee, declared several interests that did not relate to the topic of this guideline. However, there was not sufficient transparency with regard to the interdependencies and ramifications (subsidiaries) of the pharmaceutical companies involved. Thus, an unconscious bias in the decisions, e.g. with regard to antibiotic treatment, cannot be ruled out. Prof. A. Krause did not participate in voting on pharmacological treatment, in particular, antibiotic therapy.

Formal reasons for conflicts of interest:
Dr. Walter Berghoff, German Borreliosis Society (DBG), (member of the consensus group): Imprecise information stated on the interest form, for example, no income was declared from topic-related expert services provided to courts etc.

Ute Fischer, Borreliosis and FSME Association Germany (BFBD), (member of the consensus group): non-fiction author, particularly the Borreliosis Yearbook series. Imprecise information stated on the interest form, for example, no income was declared from publications.

The risk of bias through potential conflicts of interest were countered through:
- Interdisciplinary, pluralistic composition of the guideline group with the involvement of representatives with different viewpoints
- Systematic survey and assessment of the evidence by the German Cochrane Centre
- Structured consensus building moderated by an independent guideline advisor from the AWMF

Overall assessment of those involved

The group of authors comprises 33 members, seven of whom are in the steering group. According to the interest criteria, 28 members (five in the steering group) are free of conflicts of interest or only have minor topic-related conflicts of interest. This means that the criterion of having 50% members without conflicts of interest is met by the group as a whole and by the steering group.

Conflicts of interest cannot be ruled out for three members; in the case of two members, as a result of insufficient information given, or doubts about the completeness of the declaration form; in the case of an author (member of the steering group), non-participation in the voting on antibiotic treatment is deemed appropriate.
There are potentially serious conflicts of interest for two members (both in the steering group, including the lead coordinator). Their possible influence is neutralised 1) through twice as many unencumbered members of the steering group, 2) through a second coordinator, 3) through abstention of voting by the lead coordinator, 4) through an overwhelming majority of members of the author group without conflicts of interest.
In summary, the measures to limit possible conflicts of interest are deemed sufficient to ensure the independence of the decision-making used to prepare this guideline according to the criteria of the DGN and AMWF. The group of authors is well-balanced.

5. Distribution and implementation

Concept for distribution and implementation

Websites of the AWMF and DGN; translation into English and publication in an international journal with focus on evidence-based medicine; presentation of the guideline at congresses.

Supporting materials for applying the guideline

Underlying literature from the evidence process: (Dersch et al. 2015a; Dersch et al. 2014; Dersch et al. 2016a; Dersch et al. 2016b; Dersch et al. 2015b)

Discussion of possible organisational and/or financial barriers to applying the guideline recommendations

Applying the guideline recommendations

Since the recommended diagnostic testing and treatment can be performed both on an in-patient and out-patient basis depending on the nature of the symptoms, and the recommended antibiotics can be administered both orally and intravenously, few organisational problems are likely to arise when implementing the recommendations. As the recommended antibiotics are available in generic form, cost bearers should have no issues in applying the guideline.

6. Validity period and updating procedures

The guideline is valid for 3 years from the publication date (12 April 2018); 6 months before the expiry date, a literature survey shall be conducted with respect to existing systematic reviews and systematically evaluated for the subsequent period.
The correspondingly updated manuscript will be discussed as part of a new consensus procedure, and the key recommendations will be reviewed with respect to their relevance to the current situation.
Prof. Dr. S. Rauer and Dr. R. Dersch are responsible for updating the guideline in consultation with the DGN Guideline Commission.

This guideline refers to the diagnosis and treatment of neurological manifestations of Lyme borreliosis in children and adults. In future, it is to be integrated as module 2 into the planned interdisciplinary S3 guideline "Lyme Borreliosis – Diagnosis and Treatment, AWMF register no. 013-080".

Literature

1. Balshem H, Helfand M, Schunemann HJ, Oxman AD, Kunz R, Brozek J, Vist GE, Falck-Ytter Y, Meerpohl J, Norris S, Guyatt GH. GRADE guidelines: 3. Rating the quality of evidence. J Clin Epidemiol. 2011;64:401-6.
2. Bingham 1995. Neurologic manifestations in children with Lyme disease. Pediatrics. 1995 Dec;96(6):1053-6.
3. Bouwmeester W, van Ernst A, van Tulder M. Quality of low back pain guidelines improved. Spine (Phila Pa 1976). 2009;34:2562-7.
4. Brouwers MC, Kho ME, Browman GP, Burgers JS, Cluzeau F, Feder G, Fervers B, Graham ID, Grimshaw J, Hanna SE, Littlejohns P, Makarski J, Zitzelsberger L. AGREE II: advancing guideline development, reporting, and evaluation in health care. Prev Med. 2010;51:421-4.
5. Dersch R, Freitag MH, Schmidt S, Sommer H, Rauer S, Meerpohl JJ. Efficacy and safety of pharmacological treatments for acute Lyme neuroborreliosis – a systematic review. Eur J Neurol. 2015a;22:1249-59.
6. Dersch R, Freitag MH, Schmidt S, Sommer H, Rucker G, Rauer S, Meerpohl JJ. Efficacy and safety of pharmacological treatments for neuroborreliosis – protocol for a systematic review. Syst Rev. 2014;3:117.
7. Dersch R, Hottenrott T, Schmidt S, Sommer H, Huppertz HI, Rauer S, Meerpohl JJ. Efficacy and safety of pharmacological treatments for Lyme neuroborreliosis in children: a systematic review. BMC Neurol. 2016a;16:189.
8. Dersch R, Sommer H, Rauer S, Meerpohl JJ. Prevalence and spectrum of residual symptoms in Lyme neuroborreliosis after pharmacological treatment: a systematic review. J Neurol. 2016b;263:17-24.
9. Dersch R, Toews I, Sommer H, Rauer S, Meerpohl JJ. Methodological quality of guidelines for management of Lyme neuroborreliosis. BMC Neurol. 2015b:15, 242.
10. Halperin JJ, Shapiro ED, Logigian E, Belman AL, Dotevall L, Wormser GP, Krupp L, Gronseth G, Bever CT Jr. Practice parameter: treatment of nervous system Lyme disease (an evidence-based review): report of the Quality Standards Subcommittee of the American Academy of Neurology. Neurology. 2007;69:91-102.
11. Haran C. vanDriel M, Mitchell BL, Brodribb WE. Clinical guidelines for postpartum women and infants in primary care – a systematic review. BMC Pregnancy Childbirth. 2014;14:51.
12. Hassler D, Zöller L, Haude M, Hufnagel HD, Heinrich F, Sonntag HG. Cefotaxime versus penicillin in the late stage of Lyme disease – prospective, randomized therapeutic study. Infection. 1990 Jan-Feb;18(1):16-20. DOI: 10.1007/BF01644175
13. Jörbeck HJ, Gustafsson PM, Lind HC, Stiernstedt GT. Tick-borne Borrelia-meningitis in children. An outbreak in the Kalmar area during the summer of 1984. Acta Paediatr Scand. 1987 Mar;76(2):228-33.
14. Kaiser R. Neuroborreliosis [Review] [111 refs]. J Neurol 1998;245:247-55.

15. Karlsson M, Hammers-Berggren S, Lindquist L, Stiernstedt G, Svenungsson B. Comparison of intravenous penicillin G and oral doxycycline for treatment of Lyme neuroborreliosis. Neurology. 1994 Jul;44(7):1203-7. DOI: 10.1212/wnl.44.7.1203
16. Kohlhepp W, Oschmann P, Mertens HG. Treatment of Lyme borreliosis. Randomized comparison of doxycycline and penicillin G. J Neurol. 1989 Dec;236(8):464-9.
17. Ljostad U, Skogvoll E, Eikeland R, Midgard R, Skarpaas T, Berg A, Mygland A. Oral doxycycline versus intravenous ceftriaxone for European Lyme neuroborreliosis: a multicentre, non-inferiority, double-blind, randomised trial. Lancet Neurol. 2008 Aug;7(8):690-5. DOI: 10.1016/S1474-4422(08)70119-4.
18. Millner M. Kinderneurologische Manifestationen der Lyme-Borreliose. [Neurologic manifestations of Lyme borreliosis in children]. Wien Med Wochenschr. 1995;145(7-8):178-82.
19. Müllegger RR, Millner MM, Stanek G, Spork KD. Penicillin G sodium and ceftriaxone in the treatment of neuroborreliosis in children – a prospective study. Infection. 1991 Jul-Aug;19(4):279-83.
20. Mygland A, Ljostad U, Fingerle V, Rupprecht T, Schmutzhard E, Steiner I; European Federation of Neurological Societies. EFNS guidelines on the diagnosis and management of European Lyme neuroborreliosis. Eur J Neurol. 2010;17:8-16.
21. Oksi J, Nikoskelainen J, Viljanen MK. Comparison of oral cefixime and intravenous ceftriaxone followed by oral amoxicillin in disseminated Lyme borreliosis. Eur J Clin Microbiol Infect Dis. 1998 Oct;17(10):715-9.
22. Pfister HW, Einhäupl KM, Franz P, Garner C. Corticosteroids for radicular pain in Bannwarth's syndrome: A double-blind, randomized, placebo-controlled trial. Ann NY Acad Sci. 1988;539:485-7.
23. Pfister HW, Preac-Mursic V, Wilske B, Einhäupl KM. Cefotaxime vs penicillin G for acute neurologic manifestations in Lyme borreliosis. A prospective randomized study. Arch Neurol. 1989 Nov;46(11):1190-4. DOI: 10.1001/archneur.1989.00520470044025
24. Pfister HW, Preac-Mursic V, Wilske B, Schielke E, Sörgel F, Einhäupl KM. Randomized comparison of ceftriaxone and cefotaxime in Lyme neuroborreliosis. J Infect Dis. 1991 Feb;163(2):311-8. DOI: 10.1093/infdis/163.2.311
25. Rauer S, Kaiser R, Kölmel HW, Pfister HW, Schmutzhard E, Sturzenegger M, Wilske B. Neuroborreliose. In: Diener HC, Weimar C, editors. Leitlinien für Diagnostik und Therapie in der Neurologie. Stuttgart, New York: Georg Thieme; 2012. S. 513-22.
26. Skowronek-Bala B, Wesolowska E, Gergont A, Kacinski M. [Neuroborreliosis with motoric disturbations in the developmental age]. Przegl Lek. 2008;65(11):810-2.
27. Stanek G, Fingerle V, Hunfeld KP, Jaulhac B, Kaiser R, Krause A, Kristoferitsch W, O'Connell S, Ornstein K, Strle F, Gray J. Lyme borreliosis: clinical case definitions for diagnosis and management in Europe. Clin Microbiol Infect. 2011;17:69-79.
28. Thorstrand C. Belfrage E, Bennet R, Malmborg P, Eriksson M. Successful treatment of neuroborreliosis with ten day regimens. Pediatr Infect Dis J. 2002 Dec;21(12):1142-5.
