# Supplementary material for: Guidelines for diagnosis and treatment in neurology – Lyme neuroborreliosis
Source: Ger Med Sci. 2020 Feb 27;18:Doc03. doi: 10.3205/000279 (PMC7174852; doi:10.3205/000279)
Supplement: Appendix [file GMS-18-03-s-003.pdf]

Attachment 3: Appendix

Numbers in brackets refer to reference list in the article.

Appendix 1

Feder's criteria for "presumptive chronic Lyme disease"

Feder et al. have described 4 clinical categories to which patients with presumptive "chronic Lyme borreliosis" can be assigned [142]:

(1) Symptoms of an unknown cause without evidence of infection with *Borrelia burgdorferi*. In this category a combination of non-specific complaints is assumed to be a strong indication of "chronic Lyme borreliosis" [243]. However, the non-specific symptoms are found in about 10% of the normal US population regardless of whether the region is endemic to Lyme borreliosis or not [169], [170].

(2) Symptoms of a known, well-defined disease without evidence of infection with *Borrelia burgdorferi*. Here, the original diagnosis is presumed to be false (e.g. multiple sclerosis).

(3) Symptoms of an unknown cause and serology results that are positive for Lyme borreliosis, but no objective clinical findings of Lyme borreliosis now or in the past.

(4) Post-treatment Lyme disease syndrome (PTLDS). Cf. Section 4.3 and Appendix 2.

Appendix 2

The Infectious Diseases Society of America (IDSA) recommended the following diagnostic criteria for PTLDS in 2006 [27]

(1) Previous, confirmed Lyme borreliosis (according to CDC criteria) and regression or stabilisation of objective symptoms of Lyme borreliosis under a generally accepted antibiotic treatment regimen.

(2) Onset of subjective symptoms (fatigue, extensive musculoskeletal pain, cognitive disorders) within 6 months after diagnosis of Lyme borreliosis and persistence of symptoms (or chronic recurrent onset) for at least 6 months after completion of antibiotic treatment.

(3) The subjective symptoms lead to a relative impairment of daily activities.

(4) Exclusion criteria:

- Active, untreated co-infection
- Objective findings during physical examination or neuropsychological testing that explain the symptoms
- Subjective symptoms that were present before the Lyme borreliosis
- Another underlying disease that explains the symptoms (e.g. morbid obesity, sleep apnoea, narcolepsy, autoimmune diseases, drug side effects, (insufficiently treated or decompensated) cardiopulmonary diseases, endocrine diseases, malignant diseases within the last 2 years, liver diseases, depressive disorders, bipolar diseases, delusional disorders, dementia, eating disorders, drug or alcohol abuse within the last 2 years
- Laboratory and/or imaging findings that could explain the symptoms (e.g. BSG>50mm/h, abnormal serum levels for thyroid hormone, total protein, immunoglobulins, liver values, calcium, phosphorus, urea, electrolytes, creatinine. Abnormal urine test results.

Appendix 3

Table 6

Absolute results and forms of manifestation from the 8 RCTs and 8 cohort studies (modified according to [4])

Study (RCT)	Case definition	Manifestation	Neurological residual symptoms after 3–12 months	
			Doxycycline	Beta lactam antibiotics
**Ljostad 2008 [226]**	Definite (n=71)<br>Possible (n=31)	Early manifestations (Bannwarth's syndrome, cranial nerve paresis, radiculopathies) n=97 (95%)<br>Late manifestations (myelopathy, ACA with paraesthesia, encephalopathy) n=5 (5%)	28/54 (52%)	32/48 (66%) (Ceftriaxone)
**Karlsson 1994 [221]**	Probable	Not differentiable, predominantly early manifestations	6/31 (19%)	4/21 (19%) (Penicillin G)
**Kohlhepp 1989 [246]**	Possible	Early manifestations (radiculopathy, meningitis, cranial nerve paresis)	19/39 (49%)	23/36 (63%) (Penicillin G)
**Study (RCT)**	**Case definition**	**Manifestations**	**Neurological residual symptoms after 12 months**	
			**Doxycycline**	**Beta lactam antibiotics**
**Ljostad 2008 [226]**	Confirmed (n=71)<br>Possible (n=31)	See above	22/44 (50%)	19/41 (46%)
**Karlsson 1994 [221]**	Probable	See above	3/30 (10%)	3/21 (14%)
**Kohlhepp 1989 [246]**	Possible	See above	12/39 (30%)	11/36 (30%)

Study (RCT)	Case definition	Manifestations	Neurological residual symptoms after >3 months	
			Cefotaxime	Penicillin G
**Hassler 1990 [229]**	Possible	Early manifestations (radiculopathies)	14/49 (28%)	24/44 (54%)
**Pfister 1989 [228]**	3/21 Possible<br>19/21 Patients confirmed/ probable	Early manifestations (Bannwarth's syndrome, meningitis)	2/11 (18%)	2/10 (20%)
**RCT without relevant data for a defined comparison**				
**Study (RCT)**	**Case definition**	**Manifestations**		
**Oksi 1988**	Possible	Not differentiable, predominantly early manifestations		
**Pfister 1988 [245]**	3/21 Possible<br>18/21 Probable	Early manifestations (Bannwarth's syndrome)		
**Pfister 1991 [227]**	6/30 Possible<br>24/30 Confirmed/probable<br>24/30 Confirmed /probable	Early manifestations (Bannwarth's syndrome)		

Attachment 3 to: *Rauer S, Kastenbauer S, Hofmann H, Fingerle V, Huppertz HI, Hunfeld KP, Krause A, Ruf B, Dersch R, Consensus group. Guidelines for diagnosis and treatment in neurology – Lyme neuroborreliosis. GMS Ger Med Sci. 2020;18:Doc03. DOI: 10.3205/000279, URN: urn:nbn:de:0183-0002795*
Online available from: https://www.egms.de/en/journals/gms/2020-18/000279.shtml

Appendix 4

Table 7

Frequency of side effects from 6 RCTs (modified according to [4])

Beta lactam vs. doxycycline					
**Design**	**Studies**	**Beta lactam group**	**Doxycycline group**	**Side effects beta lactam**	**Side effects doxycycline**
**RCTs**	2	79	88	32 (40%)	25 (28%)
**NRS**	2	47	75	1 (2%)	5 (7%)
**Penicillin vs. cefotaxime**					
**Design**	**Studies**	**Cefotaxime group**	**Penicillin G group**	**Side effects cefotaxime**	**Side effects penicillin G**
**RCTs**	2	80	79	39 (49%)	22 (28%)

Appendix 5

Table 8

Side effects from comparison studies (modified according to [4])

Individual side effects		
**Beta lactam vs doxycycline**	**Beta lactam**	**Doxycycline**
**Ljostad, 2008 [226]**	**n=56**	**n=57**
**All AE**	26	57
**SAE**	3 (cholecystitis/stomatitis/allergy)	1 (duodenal ulcer)
**Other (not broken down by therapy)**	diarrhoea n=17, nausea n=3, nausea + diarrhoea n=2, constipation n=9, exanthema n=3	
**Karlsson 1994 [221]**	**n=23**	**n=31**
**AE**	3 (dizziness n=1, thrombophlebitis n=2)	4 (exanthema n=2, diarrhoea n=2)
**Berglund 2002 [164]**	**n=18**	**n=39**
**AE/SAE**	0	0
**Borg 2005 [247]**	**n=29**	**n=36**
**AE**	1 (leucopenia)	5 (GI issues n=3, phototoxicity n=2)
**Penicillin vs. cefotaxime**	**Penicillin**	**Cefotaxime**
**Hassler 1990 [229]**	**n=69**	**n=69**
**AE**	20 (diarrhoea n=6, Herxheimer's reaction n=14)	37 (diarrhoea n=9, Herxheimer's reaction n=28)
**SAE**	n=2 (colitis, shock)	n=2 (colitis, allergic reaction)
**Pfister 1989 [228]**	**n=10**	**n=11**
**AE**	0	0

Appendix 6

Information for patients following a tick bite (from DDG S2k-LL Cutaneous Lyme Borreliosis; AWMF register no. 013/044) [1]

1. Remove the tick as quickly as possible. Special tick tweezers or tick cards are the best way to do this.
   Pull or push the tick slowly and patiently out of the skin – without twisting or pre-treating it with oil or glue. Avoid squishing the body.
   If some of the suction organ remains in the skin (often misinterpreted as the "head"), you can remove it with a sterile needle or a curette, or have it removed by a physician. If the suction organ remains in the skin, there is no danger of the Borrelia being transferred.
2. Carefully examine your body and especially the heads of children for more ticks.
3. Observe the skin near the site of the bite for 6 weeks. A redness that appears directly after the bite as a result of the tick's saliva will disappear within several days. If the redness reappears or if the original redness enlarges to ≥5 cm, be sure to consult a doctor. This can be a sign of erythema migrans (migrating rash), the early manifestation of Lyme disease.
4. When there is a typical migrating rash near the site of the bite, it should be treated with antibiotics, preferably with doxycycline (for children aged 9 and up) or amoxicillin, even when no blood test has been carried out or if no antibodies have been detected.
5. The dissemination of the Borrelia through the blood – even without a reddening of the skin – is noticeable by a flu-like feeling without respiratory symptoms. This may be a precursor to organ disease, e.g. of the joints or the nervous system. Consult a physician who will decide whether or not a blood screening for Borrelia antibodies is necessary.
6. Early-stage Lyme disease can be completely cured when antibiotic treatment is carried out in line with the guidelines. This also prevents late manifestations.
7. It is not necessary to examine the tick for Borrelia since a positive detection does not confirm whether the Borrelia were indeed transferred to the skin and whether, in the case of a transmission, this will lead to an infection. A negative result does not exclude the possibility of a transmission.
8. Only a small proportion of the people infected with Borrelia become ill! This is why a prophylactic treatment with oral antibiotics is not recommended.

Appendix 7

Frequency of clinical manifestations for neuroborreliosis from 3 studies

Table 9

Frequency of clinical manifestations for neuroborreliosis (Kaiser 2004 [42])

Clinical manifestation	Acute (early) neuroborreliosis N=86	Chronic (late) neuroborreliosis N=15
Radiculitis spinalis<br>– Isolated radiculitis spinalis<br>– Radiculitis spinalis et cranialis<br>– Isolated radiculitis cranialis	– 73.2%<br>– 38.4%<br>– 34.9%<br>– 20.9%	
Radiculitis cranialis<br>– VII<br>– II<br>– III<br>– VI	– 55.8%<br>– 51.2%<br>– 1.2%<br>– 1.2%<br>– 2.2%	
**Myeloradiculitis**	3.5%	
**Cerebral vasculitis**	1.2%	
**Myositis**	1.2%	
**Encephalomyelitis**		100%

Table 10

Frequency of clinical manifestations for neuroborreliosis (Hansen & Lebech 1992 [41])

Clinical manifestation at the time of diagnosis	Acute (early) neuroborreliosis N=176	Late neuroborreliosis N=11
**Lymphocytic meningoradiculitis with mononeuritis (Bannwarth's syndrome)**	61%	
**Facial paresis, unilateral**	37%	
**Facial paresis, bilateral**	17%	
**Palsy, VI cranial nerve**	5%	
**Painful lymphocytic meningoradicultis (without paresis)**	24.6%	
**Subacute lymphocytic meningitis (without pain, without paresis)**	4.8%	
**Myelo-meningoradiculitis**	3.7%	
**Chronic lymphocytic meningitis (with paresis, duration of disease >6 months)**		1.6%
**Chronic progressive encephalomyelitis**		4.3%

Table 11

Frequency of clinical manifestations for neuroborreliosis (Oschmann et al. 1998 [47])

Symptoms related to neuroborreliosis	N=330
**Paresis, peripheral**	45%
**Paresis, central**	9%
**Sensory disorder, peripheral**	44%
**Sensory disorder, central**	4%
**Cranial nerve palsy, facial nerve**	39%
**Cranial nerve palsy, other**	8%
**Bladder paralysis**	5%
**Organic brain syndrome**	3%
**Parkinson syndrome**	7%
**Cerebellar ataxia**	2%
**Stroke**	1.2%
**Myositis**	0.3%

Appendix 8

Table 12

GRADE ranking of studies on antibiotics (modified according to [4])

Quality assessment								Effects		
**Number of studies**	**Design**	**Risk of bias**	**Inconsis-tency**	**Indirectness**	**Imprecision**	**Number of patients**		**RR (95% CI)**	**Absolute**	**Quality**
**Beta lactam vs. doxycycline**						**Beta lactam**	**Doxycycline**			
**Neurological symptoms after >4 months**										
**3**	RCTs	Relevant[1]	No relevant inconsis-tencies	No relevant indirectness	Relevant[2]	59/105 (56.2%)	53/124 (42.7%)	RR 1.27 (0.98–1.63)	115 more per 1000 (from 9 fewer to 269 more)	Early manifestations: ++-- LOW<br><br>Late manifestations: +--- VERY LOW (additional indirectness)
**Side effects**										
**2**	RCTs	Relevant[1]	No relevant inconsis-tencies	No relevant indirectness	Relevant[2]	24/80 (30%)	30/87 (34.5%)	RR 0.82 (0.54–1.25)	62 fewer per 1000 (from 159 fewer to 86 more)	Early manifestations: ++-- LOW<br><br>Late manifestations: +--- VERY LOW (additional indirectness)

**Neurological symptoms after >12 months**										
**3**	RCTs	Relevant[1]	No relevant inconsis-tencies	No relevant indirectness	Relevant[2]	33/98 (33.7%)	37/113 (32.7%)	RR 0.98 (0.68–1.42)	7 fewer per 1000 (from 105 fewer to 138 more)	Early manifestations: ++-- LOW<br><br>Late manifestations: +--- VERY LOW (additional indirectness)
[1]two unblinded studies, concerns regarding treatment allocation and selective reporting<br>[2]limited number of cases										
**Cefotaxime vs. penicillin**						**Penicillin**	**Cefotaxime**			
**Neurological symptoms after >4 months**										
**2**	RCTs	Relevant[1]	No relevant inconsis-tencies	No relevant indirectness	Relevant[2]	26/54 (48.1%)	16/60 (26.7%)	RR 1.81 (1.1–2.97)	216 more per 1000 (from 27 more to 525 more)	Early manifestations: ++-- LOW<br><br>Late manifestations: +--- VERY LOW (additional indirectness)
**Side effects**										
**2**	RCTs	Relevant[1]	Relevant[3]	No relevant indirectness	Relevant[2]	22/79 (27.8%)	39/80 (48.8%)	RR 0.56 (0.38–0.84)	215 fewer per 1000 (from 78 fewer to 32 fewer)	+--- VERY LOW
[1]high risk of bias<br>[2]limited number of cases<br>[3]no findings were reported in one study										

Combination of multiple antibiotics vs. single substance						Single substance	Combination			
**Neurological symptoms**										
**2**	Observa-tional study	Very serious[1]	No relevant inconsis-tencies	Relevant[2]	Relevant[3]	4/10 (40%)	2/8 (25%)	No pooling	No pooling	+---<br>VERY LOW
[1]high risk of bias<br>[2]various types of interventions, different treatment periods<br>[3]limited number of cases										
**Antibiotic treatment vs. no treatment**						**Treatment**	**No treatment**			
**Neurological symptoms**										
**3**	Observa-tional studies	Very serious[1]	No relevant inconsis-tencies	No relevant indirectness	Relevant[2]	30/94 (31.9%)	31/79 (39.2%)	No pooling	No pooling	+---<br>VERY LOW
[1]high risk of bias<br>[2]limited number of cases										

Appendix 9

Table 13

Important differential diagnoses of Lyme neuroborreliosis

Neurological manifestation	Differential diagnoses	Clinical characteristics, lab findings
**Facial paresis**	Idiopathic facial paresis	– No CSF pleocytosis<br>– Differentiation can be difficult in the early stage – especially with children
	Zoster oticus (Ramsay Hunt syndrome)	– Blisters on the outer ear canal (can be discrete or absent) and/or in the mouth<br>– Pain close to the ear<br>– Frequent hyperacusis and taste disorders<br>– Virus detection from blisters and/or CSF<br>– VZV AI
	Polyradiculitis cranialis (Miller Fisher syndrome)	– Rarely unilateral, barrier disorder<br>– No CSF pleocytosis<br>– GQ1b antibody<br>– Involvement of further cranial nerves
	Traumatic facial paresis	– Patient history<br>– Imaging
	Facial paresis with tumorous processes	– Cerebellopontine angle tumour<br>– Parotid gland tumour<br>– Meningeosis neoplastica: patient history (gradual progression)<br>– CSF and imaging

Neurological manifestation	Differential diagnoses	Clinical characteristics, lab findings
	Mastoiditis, otitis media	– ENT results<br>– Imaging
	Bacterial meningitis of another aetiology (incl. tuberculosis meningitis)	– CSF<br>– Pathogen detection in CSF
	Sarcoidosis (Heerfordt syndrome)	– Facial swelling with parotid gland<br>– Uveitis<br>– Often bilateral facial paresis<br>– Imaging<br>– Serum marker for sarcoidosis
	Melkersson-Rosenthal syndrome	– Relapsing course<br>– Geographic tongue<br>– Facial swelling
**Mono/polyradiculitis (Bannwarth's syndrome)**	Mono/polyradiculitis from other pathogens:<br>VZV, EBV, HSV, CMV (latter with immunosuppression)	– Pathogen detection in CSF
	Root compression from slipped disc, facet syndrome, sacroiliac joint syndrome, piriformis syndrome	– Symptoms depend on strain<br>– Can be mechanically triggered locally (or trigger points)<br>– Spinal imaging
	Spinal tumour (e.g. neurinoma, ependymoma); meningeosis neoplastica	– Gradual progression<br>– Imaging
	Spondylodiscitis, spinal/epidural abscess	– Can be mechanically triggered locally<br>– Inflammation parameters<br>– Imaging

Neurological manifestation	Differential diagnoses	Clinical characteristics, lab findings
**Meningitis**	Chronic meningitis (caused by pathogen, not immunodeficiency):<br>– Mycobacterium tuberculosis<br>– Treponema pallidum<br>– Mollaret meningitis (HSV2)<br>– Parameningeal focus of infection (sinusitis, mastoiditis, otitis)<br>– HSV 1 and 2<br>– Lymphocytic chorioretinitis<br>– Enteroviruses<br>– VZV (seldom)	Microbiological testing for pathogen in CSF
	Chronic meningitis (caused by pathogen, with immunodeficiency):<br>– HIV<br>– Mycobacterium tuberculosis<br>– CMV<br>– Cryptococcus neoformans<br>– Candida spp.<br>– Toxoplasma gondii	
	Chronic meningitis (not caused by pathogen):<br>– Meningeosis neoplastica<br>– M. Behcet<br>– Collagenosis<br>– Sarcoidosis<br>– Migraines with CSF pleocytosis<br>– Idiopathic steroid responsive chronic meningitis<br>– Drug-induced meningitis<br>– Leptomeningeal involvement in isolated CNS angiitis	– Microbiological differentiation of pathogen-induced cause in CSF and, if necessary, serum<br>– Cerebrospinal fluid cytology<br>– Autoimmune serology<br>– Internal-rheumatological clarification<br>– Drug history
**Myelitis**	Chronic myelitis (not caused by pathogen):	– Microbiological differentiation of pathogen-induced cause in CSF and, if necessary, serum<br>– Spinal MRI

Neurological manifestation	Differential diagnoses	Clinical characteristics, lab findings
	– Chronic progressive multiple sclerosis<br>– Neuromyelitis optica (NMO) and neuromyelitis optica spectrum disorders (NMOSD)<br>– Collagenosis<br>– Paraneoplastic<br>– After vaccination (extremely rare)	– Autoimmune serology<br>– Internal-rheumatological co-assessment<br>– Vaccination history
	Myelitis (caused by pathogen):<br>– Mycobacterium tub.<br>– Treponema pallidum<br>– HSV, VZV, enteroviruses<br><br>If immunodeficiency is present:<br>– HIV, CMV, JCV<br><br>Parainfectious with:<br>– Mycoplasma pneumoniae<br>– HSV-2, VZV, CMV, EBV, adenovirus, ECHO, mumps	– Microbiological differentiation of pathogen-induced cause in CSF and, if necessary, serum<br>– Spinal MRI
	Chronic myelopathy:<br>– Spinal canal stenosis<br>– Funicular myelosis (vitamin B12 deficiency)<br>– Degenerative diseases (ALS, spastic spinal paralysis)<br>– Spinal dural arteriovenous fistulas<br>– Radiation myelopathy<br>– Adrenoleukodystrophy<br>– Hepatic myelopathy<br>– Copper deficiency myelopathy<br>– HIV myelopathy<br>– Spinal tumours<br>– Alcoholic myelopathy	– Spinal imaging (MRI, CT, myelography, angiography)<br>– Extended lab testing<br>– Internal clarification<br>– CSF for differentiation from an infectiological cause<br>– Electrophysiology (EPs)

Participating medical societies and organisations

Steering group

Leading:
Prof. Dr. med. Sebastian Rauer – coordinator
with collaboration from Dr. med. Rick Dersch
German Society of Neurology (DGN)

PD Dr. med. Stephan Kastenbauer – deputy coordinator
German Society of Neurology

Prof. Dr. med. Heidelore Hofmann – coordinator
German Dermatology Society (DDG)

Dr. med. Volker Fingerle
German Society for Hygiene and Microbiology (DGHM)

Prof. Dr. med. Hans-Iko Huppertz
German Society of Paediatrics and Adolescent Medicine (DGKJ) and
German Society of Paediatric Infectiology (DGPI)

Prof. Dr. med. Klaus-Peter Hunfeld
The German United Society of Clinical Chemistry and Laboratory Medicine (DGKL) and INSTAND e.V.

Prof. Dr. med. Andreas Krause
German Society of Rheumatology (DGRh)

Prof. Dr. med. Bernhard Ruf
German Society of Infectious Diseases (DGI)

Advisory group of experts

Prof. Dr. R. Kaiser, Clinic for Neurology, Helios Hospital Pforzheim
Prof. Dr. H. W. Kölmel, former Clinic for Neurology, Helios Hospital Erfurt
Prof. Dr. H. W. Pfister, Neurological Clinic, Ludwig Maximilians University Munich

For the Austria Society of Neurology
Prof. Dr. E. Schmutzhard, University Hospital for Neurology – NICU, Medical University of Innsbruck, Austria

For the Swiss Neurological Society
Prof. Dr. M. Sturzenegger, University Clinic for Neurology, Inselspital, University of Bern, Switzerland

Consensus group

(Steering group is a component of the consensus group)

Prof. Dr. med. Karl Bechter
The German Association of Psychiatry, Psychotherapy and Psychosomatics (DGPPN)

PD Dr. med. Walter Berghoff
German Borreliosis Society (DBG)

Ursula Dahlem
Action Alliance Against Tick-Borne Infections Germany (OnLyme-Aktion)

Ute Fischer
Borreliosis and FSME Association Germany (BFBD)

Prof. Dr. med. Michael H. Freitag
German College of General Practitioners and Family Physicians (DEGAM)

PD Dr. med. Gudrun Gossrau
German Pain Society (DGSS)

Prof. Dr. med. Gerd Gross

Paul Ehrlich Society for Chemotherapy (PEG)

Prof. Dr. med. Rainer Müller
German Society of Oto-Rhino-Laryngology, Head and Neck Surgery

Prof. Dr. med. Mathias Pauschinger
German Society of Cardiology and Cardiovascular Research (DGK)

Prof. Dr. med. Monika A. Rieger
German Society for Occupational and Environmental Medicine (DGAUM)

Prof. Dr. med. Rainer Schäfert (mandate until 31/3/2017)
Dr. Jonas Tesarz (mandate starting 1/4/2017)
German Society of Psychosomatic Medicine and Medical Psychotherapy (DGPM) and the German College of Psychosomatic Medicine (DKPM)

Christel Schmedt
German Federal Association for Tick-Borne Diseases (BZK)

Prof. Dr. med. Stephan Thurau
German Ophthalmological Society (DOG)

Prof. Dr. rer. nat. Reinhard Wallich
German Society for Immunology (DGfI)

Dr. Hendrik Wilking
Robert Koch Institute (RKI)

Moderation

Prof. Dr. med. Ina B. Kopp
AWMF Institute for Medical Knowledge Management

The boards of the following medical societies and organisations have approved this guideline:
German Society of Neurology (DGN)
German Dermatology Society (DDG)
German College of General Practitioners and Family Physicians (DEGAM)
German Society for Occupational and Environmental Medicine (DGAUM)
German Society of Oto-Rhino-Laryngology, Head and Neck Surgery (DGHNOKHC)
German Society for Hygiene and Microbiology (DGHM)
German Society for Immunology (DGfI)
German Society of Infectious Diseases (DGI)
German Society of Cardiology and Cardiovascular Research (DGK)
German Society of Paediatrics and Adolescent Medicine (DGKJ)
The German United Society of Clinical Chemistry and Laboratory Medicine (DGKL) und INSTAND e.V.
German Society of Paediatric Infectiology (DGPI)
The German Association of Psychiatry, Psychotherapy and Psychosomatics (DGPPN)
German Society of Psychosomatic Medicine and Medical Psychotherapy (DGPM) and the German College of Psychosomatic Medicine (DKPM)
German Society of Rheumatology (DGRh)
German Pain Society (DGSS)
German Ophthalmological Society (DOG)
Paul Ehrlich Society for Chemotherapy (PEG)
Robert Koch Institute

The following organisations have not approved the guideline:
German Borreliosis Society (DBG)
Action Alliance Against Tick-Borne Infections Germany (OnLyme-Aktion)
Borreliosis and FSME Association Germany (BFBD)
German Federal Association for Tick-Borne Diseases (BZK)
