# Supplementary material for: Guidelines for diagnosis and treatment in neurology – Lyme neuroborreliosis
Source: Ger Med Sci. 2020 Feb 27;18:Doc03. doi: 10.3205/000279 (PMC7174852; doi:10.3205/000279)
Supplement: Conflict of interest [file GMS-18-03-s-004.pdf]

Attachment 4: Conflict of interest

Guideline: Neuroborreliosis

Guideline coordinators: Sebastian Rauer and Stefan Kastenbauer

AWMF register no.: 030/071

Date: September 2017

The original, fully completed declaration of interest forms are filed with the guideline coordinator/Guideline Editorial Office (EO). For reasons of transparency, all potential interests, even if they are not connected to the topic of the guideline, must be indicated. Members are listed in alphabetical order.

	Advisory or expert activities	Member of a scientific advisory board	Lecturing and training activities	Authorship/ co-authorship	Research projects/ clinical studies	Ownership interests in healthcare sector	Membership/role in interest groups	Focus of scientific or clinical activities, publications	Key involvement in education and training institutes	Personal ties *	Employer	**Assessment in relation to the guideline topic, regulatory measures if necessary**
**Karl Bechter**	no	German Borreliosis Society	no	no	no	no	Member of various scientific societies (including DGPPN)			no	Retired (Günzburg Regional Hospital)	No conflicts of interest
**Walter Berghoff**	no	no	no	no	no	no	no	no	no	no	Medical practitioner in private practice	No declaration of income for known topic-related expert activities despite request
**Ursula Dahlem**	no	no	no	no	no	no	OnLyme-Aktion.org (member, chair)	no	no	no	OnLyme-Aktion.org, action alliance against tick-borne diseases	No conflicts of interest

**Rick Dersch**	no	no	no	no	Cochrane Germany	no	DGN (member), DGLN	Neuroinfectology, neuroimmunology, neurological emergency and intensive care, CSF diagnostic testing; publications on treatment and disease progression of neuroborreliosis	no	no	University Hospital Freiburg	No conflicts of interest
**Volker Fingerle**		no				no		Epidemiology, diagnostic testing borreliosis; advising patients/colleagues		no	Bayerisches LGL	Limited conflicts at most (fees), independence remains unaffected even as member of the steering group
**Ute Fischer**	Borreliosis patients, Borreliosis and FSME Association Germany	no	Borreliosis and FSME Association Germany	Borreliosis and FSME Association Germany	no	Of the written word	Borreliosis and FSME Association Germany, German Borreliosis Society, German Federation of Journalists, Correktiv Abgeordneten Watch	None	Seminars for borreliosis consultants at the Borreliosis and FSME Association Germany	no	Borreliosis and FSME Association Germany, patient-organisation, federal association	Does not explain the exact extent of direct financial interests, for example income from publications
**Michael Freitag**	no	DAK-Gesundheit	no	no	no	no	Member of DEGAM, member of the standing guideline committee	Borreliosis, diabetes, nursing home residents; general medicine/primary care	no	no	Carl von Ossietzky University Oldenburg	No conflicts of interest

**Gudrun Gossau**	no	no	Astellas, Allergan, Grünenthal, ATI, Genzyme, Lilly	no	Allergan	no	DGSS, DMKG, DGN, IHS	Pain therapy, chronic pain, smell and pain, headaches, neuropathic pain, chronic pain, headaches in children	DMKG, regional representative, training courses: headaches, neuropathic pain, instruction at TU Dresden: lectures Q14 pain medicine, headaches	no	University Hospital Dresden, Inter-disciplinary Pain Centre	Limited, topic-related interests at most, however not critical in relation to the guideline
**Gerd Gross**	no	Sanofi, Pasteur MSD GSK		no	no	no	German Dermatology Society (DDG), Working Group for Dermatological Infectology (ADI) German Society for Allergology and Clinical immunology (DGAKI) German Society of Infectious Diseases Working Group for Dermatological Histology (ADH) European Academy of Dermatology and Venereology (EADV) International Union against Sexually Transmitted Infections (IUSTI)	HPV infections, HPV-assoc. lesions (preneoph, invasive Tks), sex. transmitted infections; changes to skin and mucous membrane, ges. dermatology and venerology (incl. Borrelia infections)	no	no	Emeritus, (State of Mecklenburg Western-Pomerania University Dermatology Clinic Rostock)	No conflicts of interest

**Heidelore Hofmann**	Arbitration board for medical liability issues, professional associations	no	Scientific conventions, training courses for the state medical associations, medical conferences	Various	Viramed Co.	no	no	Lyme borreliosis; infectious diseases	no	no	Clinic and Polyclinic of Dermatology and Allergology, University Hospital Rechts der Isar, Technical University of Munich	No conflicts of interest
**Klaus-Peter Hunfeld**	no	no	Siemens, Roche	no	no	no	DGHM, BÄMI, INSTAND, DGKL, PEG, IGLD	Medical microbiology, Lyme borreliosis, molecular detection methods, sepsis, laboratory medicine, medicine, microbiology	no	no	Krankenhaus Nordwest GmbH Frankfurt/ Main	Limited topic-related interest, expertise in laboratory diagnostic testing outweighs potential interests
**Hans-Iko Huppertz**	no	Novartis	Pfizer	no	Novartis, Pfizer, Roche, Abbvie	no	Chairman of the Society of Paediatrics and Adolescent Medicine for Northern Germany, German Academy for Paediatrics and Adolescent Medicine	Paediatric rheumatology and infectology; paediatrics and adolescent medicine	no	no	Gesundheit Nord Klinikum Bremen-Mitte	No conflicts of interest
**Reinhard Kaiser**	LG, SG, Arbitration board for medical liability issues	DGN GL on neuroborrelios is	DGN annual conference	no	no	no	DGN (member)	FSME; general neurology	Instruction of students as part of the teaching hospital at the University of Heidelberg	no	Helios Clinic Pforzheim	No conflicts of interest
**Stefan Kastenbauer (co-coordinator)**	no	no	no	no	no	no	DGN, BVDN	Neuroinfectology, general neurology in practice	no	no	Private practice, self-employed	No conflicts of interest

**Wolfgang Kölmel**	Guideline group, courts	DMSG	Lectures at DGN	no	no	no	DMSG**,** DGN	Inflammatory diseases of the CNS, neuro-ophthalmolo-gy	Fresenius Institute for speech therapy	no	Private practice	No conflicts of interest
**Ina B. Kopp, AWMF (moderator)**	German Accreditation Body (DAkkS), Ludwig Boltzmann Society	AQUA Institute, Agency for Quality in Medicine (ÄZQ)	German Veterinary Medical Society (DVG), German Association of Oral Implantology (DGI), Austrian Society of Dermatology and Venereology (ÖGDV), German Society of Internal Medicine (DGIM), Hessen State Chamber of Physicians (LÄK Hessen), German Association of Senior Hospital Physicians (VLK), Stiftung Gesundheitswissen Hessen State Chamber of Physicians (LÄK Hessen), Academy of Public Health Services	Schattauer Verlag	German Cancer Aid (DKH), Agency for Quality in Medicine (ÄZQ), German Research Foundation (DFG)	no	Expanded planning group for the National Health Care Guidelines Programme of the German Medical Association, National Association of Statutory Health Insurance Physicians and AWMF (member), steering committee for the Oncology Guidelines Programme of the German Cancer Society, German Cancer Aid and AWMF (member), steering committee of the Cooperative Network for Quality Assurance through Clinical Cancer Registries (member), standing committee for AWMF guidelines (deputy chair), Guidelines International Network, German Network for Evidence-Based Medicine, scientific	no	Seminars on AWMF guidelines for guideline developers and curriculum guideline consultants, advanced courses on AWMF guidelines for guideline consultants, workshop for the oncology guideline programme	no	Working group of the Association of Scientific Medical Societies (AWMF)	No conflicts of interest

							advisory board for Cross-sectoral Quality in Health Care (SQG) of the AQUA Institute (member), advisory board for the National Health Care Guidelines Programme of the German Medical Association, National Association of Statutory Health Insurance Physicians and AWMF (member), Advisory board for the Institute for Quality Assurance and Transparency in Healthcare (IQTIG)					
**Andreas Krause**	Abbvie, Celgene, Janssen, MSD, Novartis, Pfizer, Roche, Sanofi	Abbvie, Celgene, Janssen, MSD, Novartis, Pfizer, Roche, Sanofi	Abbvie, BMS, Celgene, Janssen, Medac, MSD, Med update, Novartis, Pfizer, Roche, Sanofi, UCB	Roche, Journal Arthritis+ Rheuma, Journal Aktuelle Rheumatolo gie	Abbvie, Amgen, BMS, Celtrion, GSK, Janssen, MSD, Novartis, Pfizer, Roche, Sandoz	no	Advisory board of the German Society for Rheumatology, Association of Rheumatological Acute Hospitals, Professional Association of German Rheumatologists, German Society for Internal Medicine, German Rheumatism League, German Association Morbus Bechterew	Rheumatoid arthritis, Spondyloarthritis, pulmonary involvement, infection-associated arthritis	no	no	Immanuel Krankenhaus Berlin GmbH	Numerous potential interests with regard to antibiotic therapies; no voting on issues relating to pharmacological, particularly, antibiotic treatment

**Rainer Müller**	no	no	no	no	no	no	ART commission at RKI, German Society of Oto-Rhino-Laryngology, Head and Neck Surgery	Laryngology, phoniatry	no	no	Med. Faculty of TU Dresden	No conflicts of interest
**Matthias Pauschinger**	no	no	no	no	no	no	no	no	no	no	Nuremberg Hospital, Medical Clinic 8 – Cardiology Department, University Hospital of Paracelsus Medical Private University	No conflicts of interest
**Hans-Walter Pfister**	no	no	no	no	no	no	DGN, DGNI, PEG	Inflammatory CNS diseases; intensive care medicine	no	no	LMU University Clinic Grosshadern, Department of Neurology	No conflicts of interest
**Sebastian Rauer (coordinator)**	LG, SG, BG, insurances	Genzyme, Biogen, Novartis	Biogen GmbH, Sanofi Genzyme, Merck Senoro, Novartis, MK+S, Teva, Diverse clinics/institutes (no industry)	Thieme Verlag: Neurologie Compact	Biogen GmbH, Novartis, Teva, Roche, Genzyme, DGN	50% ownership of the company ravo Diagnosti-ka Freiburg GmbH	Member of the DGN, member of the DGLN	Neuroimmunology, neuroinfectiology (neuroborreliosis, multiple sclerosis, paraneoplastic neurological syndrome, neuroinfectiology)	no	no	Freiburg University Hospital – Department of Neurology	No voting mandate due to specific interests

**Monika Rieger**	SVLFG	no	no	no	no	no	Board member of the German Society for Occupational and Environmental Medicine (DGAUM), Medical Advisory Board for Occupational Diseases BMAS	Until 2009: occupational biological stress, occupational zoonoses, since 2009: occupational health care research, occupational musculoskeletal and psychomental stresses and strains	As a board member of DGAUM: participation in the programme design of training courses, design of training courses as head of institute	no	State of Baden-Württemberg/ University Hospital Tübingen	No conflicts of interest
**Bernhard R. Ruf**	SLÄK	RKI	EKK	Textbook	Terevance	no	DGI	Infectious diseases and tropical medicine	Infectious diseases and tropical medicine	no	Klinikum St. Georg gGmbH	No conflicts of interest
**Rainer Schäfert (CH)**	Occasional expert activities for courts	no	Instructor at Heidelberg Institute for Psychotherapy, Network for Advanced Training in General Medicine Baden-Württemberg, instructor for patient forum for stomach and intestinal disorders at the German Society for Neurogastroenterology and motility (DGNM)	Textbook: Psychosomatik und Verhaltensmedizin, textbook: Uexküll Psychosomatische Medizin, textbook: Allgemeinmedizin Klimm, Peters-Klimm	Examining patients with high levels of somatic symptoms of distress in China, Chinese-German Symposium: "Multiple distressing somatic symptoms in China and Germany"	no	German Society of Psychosomatic Medicine and Medical Psychotherapy (DGPM)/ Psychosomatics and Psychotherapy, The German College of Psychosomatic Medicine (DKPM) /Psychosomatics and Psychotherapy	Somatoform disorders, functional gastrointestinal disorders, group therapy; psychosomatics in somatic medicine, initially assistant physician, then senior physician, later chief physician and clinical professor for psychosomatics	no	no	University Hospital of Basel	No conflicts of interest
**Christel Schmedt**	no	no	no	no	no	no	no	no	no	no	German Federal Association for Tick-Borne Diseases (BZK)	No conflicts of interest
**Erich Schmutzhard (A)**	no	Hypothermia	no	no	PI: NOSTRA Study, steering comm: NEWTON Study	no	no	no	no	no	Medical Univ. Innsbruck	No conflicts of interest

**Helmut Sitter (moderator)**	no	no	no	no	no	no	no	Methodology clinical studies, meta-analyses, guidelines	no	no	Phillips Universität Marburg	No conflicts of interest
**Klaus Stark**	no	no	no	no	no	no	no	Infection epidemiology, zoonoses etc.	no	no	Robert Koch-Institut	No conflicts of interest
**Mathias Sturzenegger (CH)**	no	no	Association of Swiss General Practitioners	no	no	no	Board member of the Swiss Headache Society, board member of the Swiss Parkinson Society, board member of the Annemarie Opprecht Foundation	Cerebrovascular diseases, headaches; acute care ward University Clinic for Neurology	no	no	University Clinic for Neurology, Inselspital, University of Bern	No conflicts of interest
**Jonas Tesarz**	no	no	Working group EMDR in pain therapy, German Society for Manual Medicine/ Association for Muscle Pain	Practical manual: EMDR in der Schmerz-therapie KlettCotta Verlag, chapter: Somato-forme Störungen Springer	BMBF	no	German Society of Psychosomatic Medicine and Medical Psychotherapy (DGPM)/ Psychosomatics and Psychotherapy, German Society for Manual Medicine/ Manual Medicine and Chirotherapy, Association for Muscle Pain/ Scientific Aspects of Muscle Pain, Professional Association for Users of the Psychotherapeutic Method, Eye Movement Desensitization and Reprocessing (EMDRIA)	Chronic pain and functional physical complaints, physical activity and pain, psychosocial factors and chronic back pain, EMDR in pain therapy; internal medicine, psychosomatics, consultation service	Working group: EMDR in pain therapy	no	Clinic for General Internal and Psychosomatic Medicine/ Heidelberg University Hospital	No conflicts of interest

**Stephan Thurau**	no	European Medicines Agency (EMA), Allergan, Panoptes GmbH Wien, Santen Inc., AbbVie	Allergan, AbbVie, Santen, Novartis	no	Santen Inc., AbbVie, Panoptes GmbH Wien	Patent: systemic therapeutic agent for autoimmune, non-infectious uveitis	Uveitis Department of the German Ophthalmological Society, German Uveitis Working Group Patient Group	Autoimmune diseases of the eye	no	no	University Eye Hospital of the University of Munich	No conflicts of interest
**Reinhard Wallich**	no	no	no	no	no	no	Member of the German Society for Immunology	Escape mechanisms of Borrelia, development of vaccines against Lyme borreliosis	no	no	Retired	No conflicts of interest
**Hendrik Wilking**	no	European Center for Disease Control	Charite Berlin	no	no	no	no	Epidemiological studies on the incidence of infections in the German population	no	no	Robert Koch Institute	No conflicts of interest

* to an authorised representative of a company in the health sector, as a partner or first-degree relative
